# The new rank-based concentration index: Further analysis and properties

**DOI:** 10.1371/journal.pone.0343034

**Published:** 2026-02-24

**Authors:** Tarald O. Kvålseth

**Affiliations:** Department of Mechanical Engineering and Department of Industrial & Systems Engineering, University of Minnesota, Minneapolis, Minnesota, United States of America; Air University, PAKISTAN

## Abstract

Additional properties and generalizations are explored for a recently introduced concentration index *C*_*K*_. The *C*_*K*_ is based on both the distribution of a set of proportions (probabilities) as well as their ranks. The *C*_*K*_ is closely related to and proposed as a preferred alternative to the widely used *Q* that equals the sum of quadratic terms (proportions). Besides the use of *C*_*K*_ and *Q* as measures of market or industry concentration, with the proportions being market shares, *C*_*K*_ or its potential transformations can be used as alternative measures in a variety of real measurement situations for which *Q* has been applied. The extended analysis of *C*_*K*_ includes the proof that *C*_*K*_ is a convex function, which makes it capable of decomposition analysis. The sensitivity and transfer effect of *C*_*K*_ due to changes in the distribution of the proportions is studied. Derivation is given for the so-called numbers equivalent of *C*_*K*_ and for its probability interpretation. Generalizations of *C*_*K*_ are considered for changing the relative emphasis of the component proportions. Randomly generated distributions exemplify the limited effect on *C*_*K*_ from excluding the smallest proportions that are often unavailable in real situations. Numerical comparisons between *C*_*K*_ and other concentration indices are presented for a wide variety of firms or industries. A statistical inference procedure is presented for appropriate situations.

## 1 Introduction

The importance of measuring concentration, especially market or industry concentration, is evidenced by the number and variety of measures or indices that have been proposed over the years (e.g., [1]). Those measures have all been defined in terms of proportions or probabilities $p_{\text{1}},...,p_{n}$, or market shares in the case of market (industry) concentration, with $p_{i}\geq \text{0}$ for i=1,...,n and $\sum_{i=\text{1}}^{n}p_{i}=\text{1}$ (or 100%). The most popular one is simply the following sum of quadratic terms:


$$Q(P_{n})=\sum_{i=\text{1}}^{n}p_{i}^{\text{2}}.$$ (1)

As a measure of market concentration, for example, it is known as the Herfindahl-Hirschman index after Herfindahl [2] and Hirschman [3].

However, since those various proposed indices, including (1), lack an important property, the *value-validity property*, an alternative index based on the ranked components of the distribution $P_{n}=\left(p_{\text{1}},...,p_{n}\right)$ has recently been introduced by Kvålseth [1] as follows:


$$C_{K}(P_{n})=\sum_{i=\text{1}}^{n}p_{\left[i\right]}/i,\;p_{\left[\text{1}\right]}\geq p_{\left[\text{2}\right]}\geq \cdots \geq p_{\left[n\right]}.$$ (2)

That is, with the $p_{i}$’s arranged in descending order (tied or equal $p_{i}$’s may be arranged in any order), $C_{K}\left(P_{n}\right)$ is simply defined as the largest $p_{i}$ divided by 1, plus the second largest $p_{i}$ divided by 2, etc. Thus, $C_{K}\left(P_{n}\right)$ is seen to be simply the weighted mean reciprocal rank, i.e., the reciprocal of the ranks 1,...,*n* weighted with the respective ranked $p_{\left[i\right]}$’s.

This index in (2) was first briefly introduced by Kvålseth [4] as a general measure of homogeneity for categorical data. A particular form of the expression in (2) with $p_{\text{1}}=\cdots =p_{n}=\text{1}/n$ and referred to as the *mean reciprocal rank* is also being used for information retrieval and ranking systems (e.g., [5,6]). Besides being a weighted mean of the reciprocal ranks, the $C_{K}(P_{n})$ in (2) can also be interpreted in terms of a statistical expectation as the expected reciprocal rank of a randomly chosen observation. Thus, in terms of a random variable *X* that can take on values 1, 1/2, ..., 1/n with the respective ordered probabilities $p_{\left[\text{1}\right]},p_{\left[\text{2}\right]},...,p_{\left[n\right]}$, $C_{K}(P_{n})$ becomes the expected value of *X*.

While $C_{K}(P_{n})$ in (2) was primarily introduced as a measure of market concentration, the versatility of $Q(P_{n})$ in (1) also extends to $C_{K}(P_{n})$ because of a close approximate functional relationship between the two indices [1]. Such variety of applications include $Q(P_{n})$ as a measure of biological species concentration [7], coincidence in cryptology [8], political consensus (e.g., [9]), and accounting harmonization and standardization (e.g., [10]). The complement 1 *− Q*(*P*_*n*_) has been used as a measure of biological diversity (e.g., [11, Ch. 4]), qualitative variation ([12, pp. 70–71]), linguistic diversity ([13,14]), ethnic fractionalization ([15]), political fractionalization ([16, Ch. 2]), quadratic entropy ([17,18, pp. 174–176]; [19,20]). Other functions of *Q*(*P*_*n*_) include 1*/Q*(*P*_*n*_) as a measure of diversity (e.g., 11, Ch. 4]) and *−*log*Q*(*P*_*n*_) as both the *collision entropy* [21] and as a measure of biological diversity ([22, p. 311]). Thus, being an approximate function of $Q(P_{n})$, but having the advantage of the value-validity property, the $C_{K}(P_{n})$ in (2) may have other potential applications besides as a measure of market concentration.

With the recent introduction of $C_{K}(P_{n})$, its various properties were defined and discussed [1]. Those properties included such generally well-known characteristics as *continuity*, *symmetry*, *zero-indifference* (adding one or more zero-probability components does not affect $C_{K}(P_{n}))$, *Schur-convexity*, and *value validity*. With emphasis on market (industry) concentration, the $C_{K}(P_{n})$ was compared with other frequently used concentration indices using both randomly generated data and real market-share data. The $C_{K}(P_{n})$ was also considered in terms of economic theory and market competition, leading to merger implications equivalent to those based on the Herfindahl-Hirschman index [2,3]. Since these properties, discussions, and results were given in a readily available open-access publication [1], there is no need for a repetition.

Rather, the purpose of the present paper is to identify and prove additional properties with relevant and important implications. One such property is the convexity of $C_{K}(P_{n})$, which permits subsystem decompositions. A simple probability interpretation of $C_{K}(P_{n})$ is defined from its functional relationship with $Q(P_{n})$. Expressions will be derived for computing the lower bound on $C_{K}(P_{n})$ and also the so-called numbers equivalent. The transfer and elasticity properties of $C_{K}(P_{n})$ will be considered as will be its potential parameterized generalizations. Randomly generated distributions $P_{n}=\left(p_{\text{1}},...,p_{n}\right)$ will be used to demonstrate the effect of ignoring the smallest $p_{i}$’s, which are often excluded from real data. Real market-share data for a variety of firms (industries) are used to compare the types of numerical values taken on by $C_{K}$ and other concentration indices. A statistical inference procedure will also be derived and exemplified.

## 2 Properties of ${𝐂}_{𝐊}$

Before discussing the properties of $C_{K}$, a point should be made about the notation used throughout this paper. Thus, with any concentration index *C* being a function of the distribution $P_{n}=\left(p_{\text{1}},...,p_{n}\right)$, it would be mathematically most correct to use $C(P_{n})$ to denote the value of the index (function) *C*. However, as a matter of simplicity and convenience and when there is no chance of ambiguity, *C* will be used to denote both the index and its value for $P_{n}$.

In the introductory paper on $C_{K}$ in (2), various properties discussed can be concisely outlined as follows ([1]):

(P1) $C_{K}$ is simple, comprehensible, and meaningfully interpretable;

(P2) $C_{K}$ takes on its extreme values for the distributions


$$P_{n}^{\text{0}}=(\text{1}/n,...,\text{1}/n),\;P_{n}^{\text{1}}=(\text{1},\;\text{0},\;\text{0},...,\text{0}).$$ (3)

with


$$C_{K}(P_{n}^{\text{0}})=\frac{\text{1}}{n}\sum_{i=\text{1}}^{n}\text{1}/i,\;C_{K}(P_{n}^{\text{1}})=\text{1};$$ (4)

(P3) *C*_*K*_ is (permutation) symmetric with respect to *p*_1_*,..., p*_*n*_;

(P4) *C*_*K*_ is zero indifferent (expansible), i.e., $C_{K}(p_{\text{1}},...,p_{n,}\text{0},...,\text{0})=C_{K}(p_{\text{1}},...,p_{n})$;

(P5) *C*_*K*_ is strictly Schur-convex;

(P6) *C*_*K*_ has the value-validity property.

### 2.1 Convexity of ${𝐂}_{𝐊}$

The fact that $C_{K}$ is strictly Schur-convex (Property (P5)) does not imply that $C_{K}$ is convex, which is a stronger requirement. In order to prove the convexity of $C_{K}$, a more general formulation will be used and of which $C_{K}$ is a particular member.

Thus, consider a general class of concentration measures defined as


$$C(P_{n},W_{n})=\sum_{i=\text{1}}^{n}w_{\left[i\right]}p_{\left[i\right]},\;w_{\left[\text{1}\right]}\geq w_{\left[\text{2}\right]}\geq \cdots \geq w_{\left[n\right]}$$ (5)

where $w_{\left[i\right]}$ and $p_{\left[i\right]}$ are both arranged in descending order for i=1,...,n. Then, for any ordered distributions $P_{\left[n\right]}=\left(p_{\left[\text{1}\right]},...,p_{\left[n\right]}\right)$ and $Q_{\left[n\right]}=\left(q_{\left[\text{1}\right]},...,q_{\left[n\right]}\right)$ and for any constant α∈[0, 1], it follows immediately from the definition in (5) that


$$\begin{array}{c} C\left[\alpha P_{\left[n\right]}+(\text{1}-\alpha )Q_{\left[n\right]},W_{\left[n\right]}\right]=\alpha C\left(P_{\left[n\right]},W_{\left[n\right]}\right)+(\text{1}-\alpha )C(Q_{\left[n\right]},W_{\left[n\right]})\\ \;\;\;\;\;\;\;\;\;\;\;\;\;=\alpha C(P_{n},W_{n})+(\text{1}-\alpha )C(Q_{n},W_{n}) \end{array}$$ (6)

where the last equality follows from the (permutation)symmetry of *C*, i.e., the value of *C* is invariant with respect to any permutations of the unordered $p_{i}$’s and $W_{\left[n\right]}$ is always ordered (ranked) as $P_{\left[n\right]}$. Next, as an immediate consequence of the definition of majorization ([23, p. 8]),


$$\alpha P_{n}+(\text{1}-\alpha )Q_{n}≺\alpha P_{\left[n\right]}+(\text{1}-\alpha )Q_{\left[n\right]}$$ (7)

where the symbol ≺ means that the left side of (7) is majorized by the right side. Then, from (7) and the fact that the type of expression as in (5) is (strictly) Schur-convex [23, pp 160, 639],


$$C\left[\alpha P_{n}+(\text{1}-\alpha )Q_{n},W_{n}\right]\leq C\left[\alpha P_{\left[n\right]}+(\text{1}-\alpha )Q_{\left[n\right]},W_{\left[n\right]}\right].$$ (8)

Finally, from (6) and (8),


$$C\left[\alpha P_{n}+(\text{1}-\alpha )Q_{n},W_{n}\right]\leq \alpha C(P_{n},W_{n})+(\text{1}-\alpha )C(Q_{n},W_{n})$$ (9)

which completes the proof that *C* in (5) is a convex function of $p_{\text{1}},...,p_{n}$. With $C_{K}$ in (2) being a particular member of *C* in (5), with $w_{\left[i\right]}=\text{1}/i$ for all *i*, this result proves that $C_{K}$ is convex.

Note that *C* in (5) is convex, but not strictly convex. Had *C* been strictly convex, then the inequality in (9) would have been strict and *C* in (5) and hence $C_{K}$ in (2) could not have complied with the value-validity requirement [1]. With $P_{n}$ and $Q_{n}$ in (6)-(9) replaced with $P_{n}^{\text{1}}$ and $P_{n}^{\text{0}}$ in (3), respectively, the value-validity property requires that


$$C\left[\alpha P_{n}^{\text{1}}+(\text{1}-\alpha )P_{n}^{\text{0}},W_{n}\right]=\;\alpha C(P_{n}^{\text{1}},W_{n})+(\text{1}-\alpha )C(P_{n}^{\text{0}}),\;\text{0}\leq \alpha \leq \text{1}$$ (10)

which is clearly satisfied by *C* in (5).

### 2.2 Comment on ${𝐂}_{𝐊}({𝐏}_{𝐧}^{0})$

The $\sum_{i=\text{1}}^{n}\text{1}/i$ component of $C_{K}(P_{n}^{\text{0}})$ in (4) is recognized as the *n*-th *harmonic number* and is of considerable mathematical interest. It is also well known that the logarithmic expression log(n+1/2)+0.5772, with 0.5772 being the *Euler’s constant* (to 4 decimal places), converges quite rapidly to $\sum_{i=\text{1}}^{n}\text{1}/i$ with increasing *n*. Therefore, an approximate expression for $C_{K}(P_{n}^{\text{0}})$ can be defined as


$$C_{K}(P_{n}^{\text{0}})\approx \left[\text{log}(n+\text{1}/\text{2})+\text{0}.\text{5}\text{7}\text{7}\text{2}\right]/n.$$ (11)

This approximation is adequate for all practical purposes. In fact, it is found to be correct to at least 3 decimal places when n>5.

It could, of course, be argued that although $C_{K}(P_{n}^{\text{0}})$ is mathematically interesting and easily computable from (11), a more convenient and intuitively reasonable lower bound on a concentration index would be 1/*n* (as in the case of *Q* in (1)). In order to be defined over the interval [1/*n*, 1], $C_{K}$ could be transformed as follows:


$$C_{K}^{•}(P_{n})=\text{1}-\left(\frac{n-\text{1}}{n}\right)\left(\frac{\text{1}-C_{K}(P_{n})}{\text{1}-C_{K}(P_{n}^{\text{0}})}\right).$$ (12)

Note, however, that the transformed index in (12) lacks the zero-indifference property (P4). Also, the true *n* may not necessarily be known in all real situations.

### 2.3 Numbers equivalent of ${𝐂}_{𝐊}$

Some prefer that a concentration measure should be of a so-called *number equivalent* or *effective number* form (e.g., [24]). For any distribution $P_{n}={(p}_{\text{1}},...,p_{n})$, the numbers equivalent $NC_{K}$ of $C_{K}$ in (2) can most concisely be defined by the approximate expression


$$C_{K}(P_{n})\approx C_{K}(\text{1}/NC_{K},...,\text{1}/NC_{K})$$ (13)

where $NC_{K}$ is the nearest integer that makes this approximation as accurate as possible. For any given $C_{K}(P_{n})$, the value of $NC_{K}$ in (13) could be determined by means of a search procedure or by trial and error based on the $C_{K}(P_{n}^{\text{0}})$ expression in (4) or (11).

An alternative approach to obtain $NC_{K}$ would be to explore some potential approximate functional relationship between $C_{K}(P_{n}^{\text{0}})$ in (4) and *n*. Based on exploratory graphical analysis and statistical regression analysis, with parameter estimates rounded off to convenient fractions, the following fitted model has been derived:


$$\hat{n}=\left(\frac{\text{1}\text{3}}{\text{5}}\right){\left(\frac{\text{1}}{C_{K}(P_{n}^{\text{0}})}-\frac{\text{1}}{\text{2}}\right)}^{\text{5}/\text{4}}.$$ (14)

For the fitted model in (14), with the 25 data points for *n* = 2,4,6,...,50, the coefficient of determination $R^{\text{2}}$, when properly computed [25], is found to be

$R^{\text{2}}=\text{1}-\sum {\left(\;n-\hat{n}\right)}^{\text{2}}/\sum {\left(n-\overset{―}{n}\right)}^{\text{2}}=\text{0}.\text{9}\text{9}\text{9}\text{7}$. When the predicted $\hat{n}$ is rounded off to the nearest integer, it is found that $\hat{n}=n$ for *n* = 1,2,...,50.

The expressions in (13)-(14) can then be used to determine the numbers equivalent $NC_{K}$ as


$$\;NC_{K}=\left(\frac{\text{1}\text{3}}{\text{5}}\right){\left(\frac{\text{1}}{C_{k}(P_{n})}-\frac{\text{1}}{\text{2}}\right)}^{\text{5}/\text{4}}$$ (15)

for any given distribution $P_{n}={(p}_{\text{1}},...,p_{n})$. It needs to be emphasized, however, that $NC_{K}$ being a non-linear function of $C_{K}(P_{n})$ does not meet the value-validity condition in (10) with $w_{\left[i\right]}=\text{1}/i$ for i=1,...,n. Nevertheless, $NC_{K}$ does provide an alternative interesting interpretation of $C_{K}(P_{n})$. For example, consider a market with 30 firms and $C_{K}(P_{\text{3}\text{0}})=\text{0}.\text{4}\text{5}$ for which $NC_{K}=\text{5}.\text{1}\text{3}\text{ or }\text{5}$ from (15), which means that this 0.45 concentration is equivalent to that of a market with 5 firms of equal size (market share).

### 2.4 Probability interpretation of ${𝐂}_{𝐊}$

An important result from the original paper on $C_{K}$ in (2) is the close functional relationship between $C_{K}$ and the quadratic measure *Q* in (1) [1]. Specifically, in terms of natural (base-*e*) logarithms and an exponential term $e^{\text{3}}$, it was established that


$$C_{K}(P_{n})\approx \left(\frac{\text{1}}{\text{3}}\right)\text{log}\left[\left(e^{\text{3}}-\text{1}\right)Q(P_{n})+\text{1}\right]$$ (16)

with a high degree of accuracy $\left(R^{\text{2}}=\text{0}.\text{9}\text{9}\right)$. Since *Q* lacks the value-validity property, (16) can be used as a transformation into $C_{K}$ that does have this property.

The approximate relationship in (16) can be inverted into


$$Q(P_{n})\approx \frac{e^{{\text{3}C}_{K}(P_{n})}-\text{1}}{e^{\text{3}}-\text{1}}$$ (17)

as a good approximation. This expression provides $C_{K}$ with another intuitively appealing interpretation: the probability that two randomly chosen observations belong to the same category. In the preceding market concentration example with $C_{K}(P_{\text{3}\text{0}})=\text{0}.\text{4}\text{5}$, $Q(P_{\text{3}\text{0}})\approx \text{0}.\text{1}\text{5}$ from (17). This result means that if two products are chosen at random from within a market, the probability is about 0.15 that they were both produced by the same firm.

### 2.5 Sensitivity and transfer of ${𝐂}_{𝐊}$

A potentially interesting characteristic of $C_{K}$ is its sensitivity to the individual components and the form of the distribution $P_{n}=(p_{\text{1}},...,p_{n}$ or of its rank ordered form $P_{\left[n\right]}=(p_{\left[\text{1}\right]},...,p_{\left[n\right]}).$ This can simply be done by taking partial derivatives of the relative terms $p_{\left[i\right]}=n_{\left[i\right]}/N$ for *i* = 1,...,*n* and $N=\sum_{i=\text{1}}^{n}n_{\left[i\right]}$ and by treating each $n_{\left[i\right]}$ as a continuous variable for mathematical purpose. Thus, the sensitivity of $C_{K}$ to a small change in $n_{\left[i\right]}$, with all other $n_{\left[j\right]}$ kept fixed, can be defined as


$$\frac{\partial C_{K}(P_{\left[n\right]})}{\partial n_{\left[i\right]}}=N^{-\text{1}}(\text{1}/i-C_{K}(P_{\left[n\right]})),\;i=\text{1},...,n.$$ (18)

With the interest being the change in $C_{K}$, irrespective of being an increase or decrease, the absolute value $\left|\partial C_{K}/\partial n_{\left[i\right]}\right|$ from (18) provides some clear indications of the sensitivity of $C_{K}$ to small changes in $P_{\left[n\right]}$. A most striking overall observation would seem to be that *C* is most sensitive to changes in the distribution $P_{\left[n\right]}$ towards its upper and lower ends. That is, this sensitivity increases with decreasing *i* for $i<\text{1}/C_{K}$ and with increasing *i* for $i>\text{1}/C_{K}$. For specific components, $C_{K}$ is particularly sensitive to changes in the extreme components $p_{\left[\text{1}\right]}$ and $p_{\left[n\right]}$.

A related characteristic of $C_{K}$ is the so-called *transfer*, i.e., the effect on $C_{K}$ when transferring a small amount ∈ from a smaller $p_{\left[j\right]}$ to a larger $p_{\left[i\right]}$. Such a transfer will cause the value of $C_{K}$ to increase as a consequence of the Schur-convexity of $C_{K}$ (Property (P5)) [23, Ch. 1]. With the restriction that $\in \leq p_{\left[j\right]}$ and $\in \leq (p_{\left[i\right]}-p_{\left[j\right]})$, the *transfer effect* on $C_{K}$ may be defined as


$$\begin{array}{c} TC_{Kij}=\underset{\in \to \text{0}}{\text{lim}}\left\{\frac{\partial }{\partial \in }\left[\frac{C_{K}(p_{\left[\text{1}\right]},...,p_{\left[i\right]}+\in ,...,p_{\left[j\right]}-\in ,...,p_{\left[n\right]})-C_{K}(P_{\left[n\right]})}{C_{K}(P_{\left[n\right]})}\right]\right\}\\ =\frac{\text{1}}{C_{K}(P_{\left[n\right]})}\left(\frac{\text{1}}{i}-\frac{\text{1}}{j}\right),\;i<j \end{array}$$ (19)

which is a similar form of definition to that used by Cowell [26, pp. 57, 154–156] for measures of inequality, except for the relative (versus absolute) difference used in (19). This expression shows that the relative effect on $C_{K}$ from a small transfer from $p_{\left[j\right]}$ to $p_{\left[i\right]}$ depends on their rank difference, but not explicitly on their values. The extreme effect occurs with a transfer from $p_{\left[n\right]}$ to $p_{\left[\text{1}\right]}$.

It may be of interest to compare these sensitivity and transfer effects of $C_{K}$ with those corresponding to *Q* in (1). Therefore, when the equivalent expressions to (18) and (19) are applied to *Q*, the following formulations are obtained:


$$\frac{\partial Q(P_{\left[n\right]})}{\partial n_{\left[i\right]}}=\text{2}N^{-\text{1}}\left(\frac{n_{\left[i\right]}}{N}-Q(P_{\left[n\right]})\right);\;TQ_{ij}=\frac{\text{2}(p_{\left[i\right]}-p_{\left[j\right]})}{Q(P_{\left[n\right]})},\;i<j.$$ (20)

The general results from (18)-(20) show that the form of the sensitivity to changes in $P_{\left[n\right]}$ and the transfer effects are comparable for the two measures, with the difference that those of $C_{K}$ are determined by the ranks while those of *Q* depend on the ranked $p_{\left[i\right]}$‘s.

## 3 m-Category $C_{Km}$

It is clear from the definition of $C_{K}$ in (2) that when a number of $p_{i}$’s are very small, ignoring those from the computation of $C_{K}$ only marginally affects the value of $C_{K}$. In practice, this characteristic of $C_{K}$ is in effect an advantage since reported data often ignore very small $p_{i}$’s or group them into an “all others” category. It is therefore worth determining more closely the effect of such exclusion on the value of $C_{K}$.

Therefore, expressing $C_{K}$ as


$$C_{K}(P_{n})=\sum_{i=\text{1}}^{m}p_{\left[i\right]}/i\;+\sum_{i=m+\text{1}}^{n}p_{\left[i\right]}/i\;=C_{Km}(P_{n})+E_{m}(P_{n})$$ (21)

the concern is basically with the size of the “error” term $E_{m}(P_{n})$. From majorization theory [23, Ch. 1], the following majorization applies:


$$\left(\frac{\sum_{i=m+\text{1}}^{n}p_{\left[i\right]}}{n-m},...,\frac{\sum_{i=m+\text{1}}^{n}p_{\left[i\right]}}{n-m}\right)≺\left(p_{\left[m+\text{1}\right]},...,p_{\left[n\right]}\right)≺\left(\sum_{i=m+\text{1}}^{n}p_{\left[i\right]},\text{0},...,\text{0}\right)$$ (22)

so that from (22) and the Schur-convexity of $E_{m}$ in (21), the following inequality is obtained:


$$\left(\frac{\sum_{i=m+\text{1}}^{n}p_{\left[i\right]}}{n-m}\right)\sum_{i=m+\text{1}}^{n}\frac{\text{1}}{i}\leq E_{m}(P_{n})\leq \frac{\sum_{i=m+\text{1}}^{n}p_{\left[i\right]}}{m+\text{1}}.$$ (23)

It is clear from (23), especially from the upper bound, that little information is lost by disregarding the smallest $p_{i}$’s if *m* is not small.

The extent of $E_{m}$ in (23) can also be examined empirically by using various distributions $P_{n}=\left(p_{\text{1}},...,p_{n}\right)$. Thus, a random sample of such distributions was generated using the computer algorithm described in [1] in which *n* and each $p_{\left[i\right]}$ were generated as random numbers within specified intervals. Specifically, for each randomly generated integer *n,* each $p_{\left[i\right]}\;(i=\text{1},...,n)$ was generated as a random number (to the desired decimals) in decreasing order within the following intervals:


$$\begin{array}{c} \frac{\text{1}}{n}\leq p_{\left[\text{1}\right]}\leq \text{1}\\ \frac{\text{1}-\sum_{j=\text{1}}^{i-\text{1}}p_{\left[j\right]}}{n-(i-\text{1})}\leq p_{\left[i\right]}\underset{\text{min}}{\leq }\left\{p_{\left[i-\text{1}\right]},\text{1}-\sum_{j=\text{1}}^{i-\text{1}}p_{\left[j\right]}\right\}\text{ for }i=\text{2},\ldots ,n-\text{1}\\ p_{\left[n\right]}=\text{1}-\sum_{j=\text{1}}^{n-\text{1}}p_{\left[j\right]}. \end{array}$$

A total of 30 such distributions were randomly generated for n∈(10,  50]  and, for each distribution, computations were made for the values of $C_{K}$ and $C_{Km}$ in (21) for the chosen *m* = 5 and 10 as given in Table 1.

**Table 1:** Values of $C_{K}$ in (2) and $C_{Km}$ in (21) for n=5 and 10 from randomly generated $P_{n}=(p_{1},...,p_{n})$ and 10<n≤50.

Data set	n	${𝐂}_{\text{K}}$	${𝐂}_{\text{K5}}$	${𝐂}_{\text{K10}}$
1	16	0.57	0.55	0.56
2	42	0.28	0.25	0.27
3	21	0.19	0.12	0.15
4	14	0.46	0.43	0.45
5	14	0.94	0.94	0.94
6	33	0.38	0.35	0.36
7	20	0.79	0.78	0.79
8	21	0.26	0.20	0.23
9	34	0.46	0.43	0.45
10	48	0.29	0.25	0.26
11	35	0.78	0.77	0.77
12	17	0.72	0.70	0.71
13	29	0.89	0.89	0.89
14	16	0.24	0.17	0.21
15	42	0.47	0.45	0.46
16	25	0.28	0.21	0.23
17	32	0.69	0.67	0.68
18	47	0.25	0.21	0.22
19	14	0.88	0.88	0.88
20	25	0.27	0.22	0.24
21	22	0.29	0.23	0.26
22	15	0.66	0.66	0.66
23	36	0.16	0.12	0.13
24	15	0.77	0.76	0.77
25	16	0.61	0.58	0.60
26	30	0.87	0.87	0.87
27	15	0.70	0.68	0.69
28	17	0.43	0.38	0.41
29	15	0.57	0.55	0.56
30	32	0.29	0.24	0.26

As could reasonably be expected, it is apparent from Table 1 that a substantial amount of information may be lost when using a value of *m* as small as *m* = 5. For example, for the error term $E_{m}(P_{n})$ in (21), it is seen that $E_{\text{5}}(P_{n})\geq \text{0}.\text{0}\text{6}$ for Data Sets 3, 8, 14, 16, and 21, with a mean error value of 0.030 for the 30 data sets. By comparison, for *m* = 10, Table 1 shows that the errors are substantially lower and values of $C_{K}$ and $C_{K\text{1}\text{0}}$ are generally quite comparable (except for, say, Data Sets 3 and 16). The linear regression of $C_{K}$ on $C_{K\text{1}\text{0}}$ is found to be ${\hat{C}}_{K}=\text{0}.\text{0}\text{4}+\text{0}.\text{9}\text{5}C_{K\text{1}\text{0}}$ with $R^{\text{2}}=\text{0}.\text{9}\text{9}\text{9}\text{3}$, indicating the considerable agreement between the two indices.

A conservative conclusion from these results would be as follows: utilize all $p_{\text{1}},...,p_{n}$ when computing the value of $C_{K}$, but if some of the smaller $p_{i}$’s are ignored, the effect on $C_{K}$ is likely to be rather negligible.

## 4 Generalizations of $C_{K}$

The $C_{K}$ in (2) could be potentially generalized in a number of different ways by introducing some additional parameter α. One such generalization would be the following α-order weighted mean of the reciprocal ranks:


$${\mathrm{\backslash [}}_{\alpha }C_{K}\left(P_{n}\right)={\left[\sum_{i=\text{1}}^{n}p_{\left[i\right]}{\left(\frac{\text{1}}{i}\right)}^{\alpha }\right]}^{\text{1}/\alpha },\;-\infty <\alpha <\infty$$ (24)

of which $C_{K}$ is the particular member α=1. Another rather obvious generalization would be


$$C_{K\alpha }(P_{n})=\sum_{i=\text{1}}^{n}p_{\left[i\right]}/i^{\alpha },\;\alpha >\text{0}$$ (25)

with $C_{K}$ being the member α=1. Note that $C_{K\alpha }$ is also a member of the concentration class in (5) with $(\text{1}/i{)}^{\alpha }=w_{\left[i\right]}$ for i=1,...,n.

**Fig 1:**
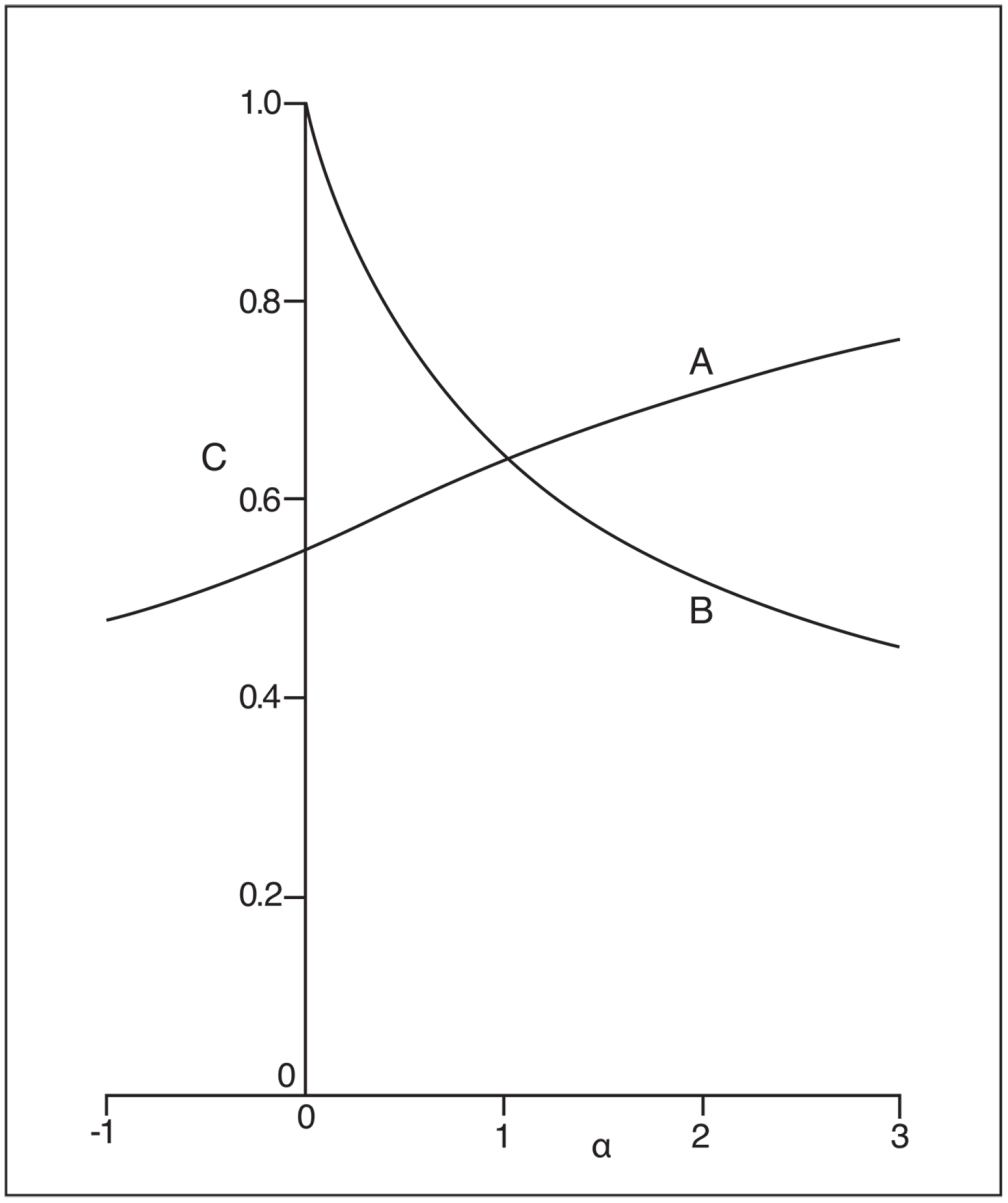
Example of $\backslash {(}_{\alpha }C_{K}(P_{n})$ in (24) (Curve A) and ${𝐂}_{K\alpha }(P_{n})$ in (25) (Curve B) as functions of the parameter α for the distribution *P*_*5*_ = (0.40, 0.30, 0.15, 0.10, 0.05).

From a property of generalized means (e.g., [27, Ch. III]), the index (family) in (24) is strictly increasing in α, whereas the parameterized index in (25) is seen to be decreasing in α for any given $P_{n}=(p_{\text{1}},...,p_{n})$. An example of these two generalized indices as functions of the parameter α for the distribution $P_{\text{5}}=(\text{0}.\text{4}\text{0},\;\text{0}.\text{3}\text{0},\;\text{0}.\text{1}\text{5},\;\text{0}.\text{1}\text{0},\;\text{0}.\text{0}\text{5})$ is given in Fig 1. As α→∞, $\backslash {(}_{\alpha }C_{K}\to \text{1}\;$ and $C_{K\alpha }\to p_{\left[\text{1}\right]}$. The two curves cross at α=1 when $\backslash {(}_{\text{1}}C_{K}=C_{K\text{1}}=C_{K}$ in (2).

The effect on these two families of indices from changing α is basically one of changing the weights or emphasis given to the different $p_{\left[i\right]}$‘s. While increasing α places increasing weights on the larger $p_{\left[i\right]}$’s for $\backslash {(}_{\alpha }C_{K}$ in (24), the effect on $C_{K\alpha }$ in (25) is the reverse. The value-validity condition in (10) can be seen to be satisfied by $C_{K\alpha }$ for all α>0, but only for α=1 in the case of $\backslash {(}_{\alpha }C_{K}$ when $\backslash {(}_{\text{1}}C_{K}=C_{K}$ in (2).

In spite of the flexibility offered by these two families of potential concentration indices, there seems to be no compelling reason to prefer any particular alternative over $C_{K}$ in (2) as a single measure. Using such curves as in Fig 1 to provide comparison between the concentration for two distributions $P_{n}$ and $Q_{m}$ is restricted by by the fact that the two curves may potentially cross such that $C_{K\alpha }(P_{n})=C_{K\alpha }(Q_{m})$ for some α and similarly for $\backslash {(}_{\alpha }C_{K}$. Nevertheless, such generalized formulations may provide some useful information in real applications. In the case of Fig 1, for example, with $C_{K}=C_{K\text{1}}{=}_{\text{1}}C_{K}$ being a compromise single concentration measure, both curves show precisely how changing emphasis on the larger or smaller component $p_{\left[i\right]}$’s affect the concentration measurement.

## 5 Statistical inferences about $C_{K}$

In situations when $p_{\text{1}},\;p_{\text{2}},\;...,p_{n}$ are multinominal random sample probabilities with $p_{i}=n_{i}/N$ for sample size $N=\sum_{i=\text{1}}^{n}n_{i}$, it may be of interest to make statistical inferences about *C*_*K*_ in (2), especially confidence-interval construction. That is, if *P*_*n*_ = (*p*_1_*,.., p*_*n*_) is the sample probability distribution and Π_*n*_ = (*π*_1_*,..., π*_*n*_) is the corresponding population distribution, one may want to make inferences about the population index *C*_*K*_(Π_n_). Besides resampling methods such as bootstrap and jackknife, such statistical inferences can be done by means of the *delta method*. The delta method is a useful and powerful results of statistical limit theory that is widely discussed in textbooks on categorical data (e.g., [28, Ch. 16], [29, Ch. 14]).

Concisely stated, it follows from the delta method applied to $C_{K}$ that the following convergence-in-distribution holds:


$$\begin{array}{c} \sqrt{N}\left[C_{K}(P_{n})-C_{K}(\Pi_{n})\right]\overset{d}{\to }\text{Normal}(\text{0},\;{\;\sigma }_{CK}^{\text{2}}) \end{array}$$ (26)

so that for a large multinomial sample of size *N*, the estimator *C*_*K*_(*P*_*n*_) is approximately normal with mean $C_{K}(\Pi_{n})$ and variance $\sigma_{CK}^{\text{2}}/N$. The accuracy of this asymptotic result depends, of course, on the sample size *N.* By taking the partial derivatives of $C_{K}(\Pi_{n})$ with respect to $\pi_{i}$ and then substituting those with the corresponding sample estimates $p_{i}$ for i=1, ...,n, the estimated variance in (26) becomes


$${\hat{\sigma }}_{CK}^{\text{2}}=\sum_{i=\text{1}}^{n}p_{\left[i\right]}{\left(\frac{\partial C_{K}(P_{n})}{\partial p_{\left[i\right]}}\right)}^{\text{2}}-{\left(\sum_{i=\text{1}}^{n}p_{\left[i\right]}\frac{\partial C_{K}(P_{n})}{\partial p_{\left[i\right]}}\right)}^{\text{2}}.$$ (27)

From the definition of $C_{K}(P_{n})$ in (2), the expression in (27) becomes


$${\hat{\sigma }}_{CK}^{\text{2}}=\sum_{i=\text{1}}^{n}p_{\left[i\right]}/i^{\text{2}}-C_{K}^{\text{2}}(P_{n}).$$ (28)

Instead of performing the statistical inferences directly on *C*_*K*_(Π_*n*_), it is preferable to use the following logarithmic transformation and its inverse:


$$L(P_{n})=\text{log}\left(\frac{C_{K}(P_{n})}{\text{1}-C_{K}(P_{n})}\right),\;C_{K}(P_{n})=\frac{\text{exp}\left[L(P_{n})\right]}{\text{1}+\text{exp}\left[L{(P}_{n})\right]}$$ (29)

since this transformation provides a more rapid convergence to normality and ensures that a confidence internal will always fall inside the [0*,* 1]-interval (e.g., [28, pp. 70, 618]; [30, p. 106]). The estimated variance of *L*(*P*_*n*_) in (29) becomes


$${\hat{\sigma }}_{L}^{\text{2}}={\left(\frac{dL(P_{n})}{dC_{K}(P_{n})}\right)}^{\text{2}}{\hat{\sigma }}_{CK}^{\text{2}}=\frac{{\hat{\sigma }}_{CK}^{\text{2}}}{C_{K}^{\text{2}}(P_{n}){\left[\text{1}-C_{K}(P_{n})\right]}^{\text{2}}}.$$ (30)

An approximate confidence interval (CI) for $L(\Pi_{n})$ then becomes


$$\text{1}\text{0}\text{0}(\text{1}-\alpha )\%CI\text{ for }L(\Pi_{n}):\;L(P_{n})\pm z_{\alpha /\text{2}}{\hat{\sigma }}_{L}/\sqrt{N}$$ (31)

where $z_{\alpha /\text{2}}$ is the standard normal quartile (e.g., $z_{\alpha /\text{2}}=\text{1}.\text{9}\text{6}$ for α=0.05 and for 95% confidence). The corresponding *CI* for *C*_*K*_(Π_*n*_) is then obtained by applying the inverse transformation in (29) to each side of the interval in (31).

As a numerical example, let $P_{\text{4}}=\left(\frac{\text{5}\text{0}}{\text{1}\text{0}\text{0}},\frac{\text{3}\text{0}}{\text{1}\text{0}\text{0}},\frac{\text{1}\text{9}}{\text{1}\text{0}\text{0}},\frac{\text{1}}{\text{1}\text{0}\text{0}}\right)$ be a sample distribution based on a sample size N=100. With $C_{K}(P_{\text{4}})=\text{0}.\text{7}\text{1}\text{6}\text{7}$, it follows from (28) that ${\hat{\sigma }}_{CK}^{\text{2}}=\text{0}.\text{5}\text{9}\text{6}\text{7}-{\left(\text{0}.\text{7}\text{1}\text{6}\text{7}\right)}^{\text{2}}=\text{0}.\text{0}\text{8}\text{3}\text{0}$ so that, from (30), ${\hat{\sigma }}_{L}^{\text{2}}=\text{2}.\text{0}\text{1}\text{3}\text{3}$. Then, with $L(P_{n})=\text{0}.\text{9}\text{2}\text{8}\text{2}$ from (29), a 95% confidence interval for L($\Pi_{n})$ from (31) becomes $\text{0}.\text{9}\text{2}\text{8}\text{2}\pm \text{1}.\text{9}\text{6}\sqrt{\text{2}.\text{0}\text{1}\text{3}\text{3}/\text{1}\text{0}\text{0}}$, or [0.6501, 1.2063]. Then, by applying the inverse transformation in (29), a 95% confidence interval for *C*_*K*_(*Π*_*n*_) becomes [0*.*66*,* 0*.*77].

## 6 Discussion

### 6.1 Illustrative example

As a real example of the computation of the new index $C_{K}$ defined in (2) and its interpretations, consider the results of the national elections in Norway in 2025. By including only the parties that received at least one percent of the votes (i.e., excluding 13 parties), the following percentage votes were obtained: 28.0, 23.8, 14.6, 5.6, 5.6, 5.3, 4.7, 4.2, 3.7 (for a total of 95.5%). Since these results are given in descending order with $p_{\left[\text{1}\right]}=\;\text{2}\text{8}.\text{0},\;p_{\left[\text{2}\right]}=\;\text{2}\text{3}.\text{8}$, etc., the value of $C_{K}$ becomes


$$\;C_{K}=\text{28}.\text{0}+\text{23}.\text{8}/\text{2}+\cdots +\text{5}.\text{6}/\text{4}+\text{5}.\text{6}/\text{5}+\cdots +\text{3}.\text{7}/\text{9}=\text{49}.\text{8}\%$$

or, in terms of proportions instead of percentages, $C_{K}=\text{0}.\text{5}\text{0}$. Note that for the two tied results of 5.6%, they are divided by consecutive ranks (rather than their mean). As a limiting case, had all 9 parties’ votes been tied, then $C_{K}\equiv (\text{1}/\text{9})+(\text{1}/\text{9})/\text{2}+\cdots +(\text{1}/\text{9})/\text{9}=\text{0}.\text{3}\text{1}$ or 31%. By comparison, a slightly larger concentration value than that of Norway’s election with $C_{K}=\text{0}.\text{5}\text{0}$ is obtained for the 2025 general election in the Czech Republic with $C_{K}=\text{0}.\text{5}\text{6}$ (56%) for 7 parties.

As a measure of political dominance (vote concentration), how can the $C_{K}=\text{0}.\text{5}\text{0}$ (50%) be interpreted in terms of the extent of such dominance? Since $C_{K}$ belongs to the interval (0, 1], $C_{K}=\text{0}.\text{5}$ would seem to imply a political dominance that is neither high nor low. However, had the votes of the top three parties been combined, the resulting value of $C_{K}=\text{0}.\text{7}\text{4}$ (74.2%) could arguably be interpreted as a high degree of political dominance.

In terms of a meaningful interpretation, what does the election result $C_{K}=\text{0}.\text{5}\text{0}$ actually mean? The answer lies in (17). That is, with $C_{K}\left(P_{\text{9}}\right)=\text{0}.\text{5}\text{0}$ in (17), Q≈0.18. This means that the probability is about 0.18 that two randomly chosen individuals voted for the same party. By comparison, the corresponding value of $Q(P_{n})$ in (1) applied directly to the above voting results, gives $Q(P_{\text{9}})=\text{0}.\text{1}\text{7}$.

As another meaningful interpretation of the election result $C_{K}=\text{0}.\text{5}\text{0}$, consider the numbers equivalent defined in (15). Then, with $C_{K}(P_{\text{9}})=\text{0}.\text{5}\text{0}$ in (15), it is found that ${NC}_{K}=\text{4}.\text{3}\text{2}$ or about 4. This result means that the political dominance of $C_{K}=\text{0}.\text{5}\text{0}$ would be the same as if Norway had had about 4 different political parties with equal party support.

### 6.2 Empirical comparison with other indices

Among the various alternative concentration indices with similar properties that have been proposed over the years [1], their numerical values may differ greatly for the same data sets. Consequently, the results and conclusions from any data analysis can depend strongly on the index being used. What sets the new index $C_{K}$ apart from other concentration indices is the so-called value-validity property (Property (P6) stated above). This property imposes a condition specifically on the numerical values taken on by a concentration index to ensure that those values can be justified as providing realistic, true, or valid representations of the concentration characteristic or attribute [1].

Therefore, it is of interest to compare values of $C_{K}$ with those of other indices for the same data sets. Consider, for example, the following indices:


$${CR}_{\text{4}}=\sum_{i=\text{1}}^{\text{4}}p_{\left[i\right]},\;HHI=\sum_{i=\text{1}}^{n}p_{i}^{\text{2}},\;RHT={\left(\text{2}\sum_{i=\text{1}}^{n}{ip}_{\left[i\right]}-\text{1}\right)}^{-\text{1}},\;DI_{\text{2}}=n{\left(\sum_{i=\text{1}}^{n}p_{i}^{\text{2}}\right)}^{\text{2}}$$ (32)

where ${CR}_{\text{4}}$ is the well-known 4-firm concentration ratio, *HHI* is the popular *Herfindahl-Hirschman* index [2, 3], *RHT* is the index by Rosenbluth [31] and Hall and Tideman [32], and ${DI}_{\text{2}}$ is a member of a parameterized family of indices by Davies [33]. For this comparative analysis, real market-share data were used as in [1] for a variety of different types of firms or industries. The results are summarized in Table 2.

**Table 2:** Values of $C_{K}$ in (2) and other concentration indices in (32) for a sample of market-share data from various types of markets or industries.

Example	*n*	${𝐂}_{K}$	${𝐂𝐑}_{\text{4}}$	*HHI*	*RHT*	${𝐃𝐈}_{\text{2}}$	Source	Market type
1	16	0.37	0.50	0.10	0.10	0.16	[34]	Airline travel
2	16	0.42	0.60	0.12	0.12	0.23	[34]	Airline travel
3	8	0.47	0.75	0.16	0.26	0.20	[35]	U.S. distilled liquor
4	10	0.45	0.64	0.14	0.15	0.20	[36]	Paints, coatings
5	10	0.38	0.52	0.11	0.13	0.12	[37]	Pharmaceuticals
6	15	0.39	0.54	0.10	0.13	0.15	[38]	Insurance companies
7	12	0.52	0.69	0.18	0.20	0.39	[39]	Weapons exporters
8	30	0.26	0.34	0.05	0.06	0.08	[40]	Car sales, Britain
9	12	0.39	0.60	0.12	0.14	0.17	[41]	Auto mfrs., U.S.
10	8	0.49	0.77	0.18	0.19	0.26	[36]	Craft beer, U.S.
11	9	0.46	0.75	0.16	0.18	0.23	[36]	Running shoe sales
12	5	0.97	1.00	0.91	0.88	4.14	[42]	Search eng., Norway
13	4	0.71	1.00	0.36	0.39	0.52	[43]	Comm. water heaters
14	3	0.90	1.00	0.70	0.67	1.47	[44]	Microprocessors
15	10	0.51	0.72	0.17	0.17	0.29	[36]	Top charter airlines
16	5	0.58	0.79	0.24	0.46	0.29	[45]	Global cigarettes, 2019
17	10	0.52	0.68	0.18	0.17	0.32	[36]	Farm mach., equip.
18	20	0.32	0.48	0.08	0.09	0.13	[46]	Global car sales
19	10	0.40	0.60	0.12	0.13	0.14	[36]	Top airlines, world
20	8	0.70	0.83	0.35	0.27	0.98	[47]	Consumer products

These results show clearly how the values of various indices can differ substantially for the same sets of data. These indices range in potential value from 0 to 1, except for ${DI}_{\text{2}}$ which can range from 0 to *n*, between the two extreme distributions in (3). Values of $C_{K}$ are seen to be consistently larger than those of *HHI* and *RHT* and frequently larger than those of ${DI}_{\text{2}}$ (except for examples 12, 14, and 20) even though the potential range of values is greatest for ${DI}_{\text{2}}$. As expected from its definition in (32), ${CR}_{\text{4}}$ values are seen to be consistently greater than those of $C_{K}$, *HHI*, and *RHT*.

For each index, the results in Table 2 show that the market (industry) with the highest market concentration was that of the leading search engines in Norway (in 2020) (Example 12) while the lowest concentration occurred for the best-selling cars in Britain (in 2018) (Example 8). Although each of these indices provide the same rank order for these two extreme cases, other order (“larger than”) comparisons vary considerably between the indices. In the case of $C_{K}$ and *RHT*, for example, $C_{K}$ shows greater concentration for airline travel (number of flights per weekday between London and New York by 30 different carriers in 2000) (Example 2) than for global pharmaceutical products (Example 5) while this order is reversed when using *RHT*. Note that these two indices are the only rank-based ones. Similarly, when comparing $C_{K}$ and *HHI*, for instance, reverse order occurs between Examples 5 and 6 and between Examples 10 and 15.

Even though $C_{K}$ is highly correlated with the other indices, with Pearson’s correlation coefficient r=0.92, 0.95, 0.93, and 0.82 between $C_{K}$ and ${CR}_{\text{4}}$, *HHI*, *RHT*, and ${DI}_{\text{2}}$, respectively, for the data in Table 2, it is clear from these exemplary data that different indices can provide substantially different and contradictory assessment of concentration. Although these data are based on the market shares $p_{\text{1}},...,p_{n}$ of a wide variety of markets or industries, similar results can be expected of any other real situations or applications involving some distribution $P_{n}=(p_{\text{1}},...,p_{n})$. In spite of the fact that many proposed concentration indices share several of the above properties (P1)-(P6), as well as the convexity property, they lack one important property: the value-validity property (P6), which only $C_{K}$ has.

## 7 Conclusion

When considering the results derived in this paper together with those previously reported [1], one conclusion would seem to be clear: $C_{K}$ has the various types of properties required of an appropriate concentration measure. One of the interesting features of $C_{K}$ is its close functional relationship to the quadratic index *Q* in (1), implying that the various applications of *Q* or its functions can also be considered for $C_{K}$.

There is, however, an important difference between $C_{K}$ and *Q*: $C_{K}$ has the value-validity property (Property(P6)), but *Q* does not since it cannot satisfy the equivalent of the equality part of (10) (with *C* = *Q* and $w_{i}=p_{i}$, i=1,...,n) because its convexity is strict. The value-validity property is considered to be necessary in order to make true and reliable difference comparisons for the concentration characteristic. The validity of such comparisons is essential for determining trend information such as changes in concentration over time periods. Without the value-validity property, different indices can produce widely differing results and conclusions as demonstrated by using real market-share data for a variety of markets (industries). In that analysis, the popular Herfindahl-Hirschman index *HHI* in (32) equals *Q* in (1).

The parameterized generalizations in (24)-(25) do provide for some potentially interesting assessment of the effect on the concentration values caused by varying the relative weight or emphasis assigned to the ordered distribution components ${\text{p}}_{\left[\text{1}\right]},...,{\text{p}}_{\left[\text{n}\right]}$. However, as a choice for a single concentration index, there would seem to be no particular reason for a preference other than ${\text{C}}_{\text{K}}$.

## Supporting information

S1 TableUnderlying data distributions $P_{n}$.(PDF)
