# Supplementary material for: The new rank-based concentration index: Further analysis and properties
Source: PLoS One. 2026 Feb 24;21(2):e0343034. doi: 10.1371/journal.pone.0343034 (PMC12931799; doi:10.1371/journal.pone.0343034)
Supplement: S1 Table — Underlying data distributions Pn. (PDF) [file pone.0343034.s001.pdf]

Underlying data distributions P_n

FOR RANDOM NUMBER N = 4 P1...PN IS

P 1= 0.585945
P 2= 0.177765
P 3= 0.120537
P 4= 0.115753

FOR RANDOM NUMBER N = 16 P1...PN IS

P 1= 0.422202
P 2= 0.144241
P 3= 0.126920
P 4= 2.80345E-02
P 5= 2.52608E-02
P 6= 2.38377E-02
P 7= 2.34925E-02
P 8= 2.32748E-02
P 9= 2.31592E-02
P 10= 2.28518E-02
P 11= 2.28202E-02
P 12= 2.28107E-02
P 13= 2.27744E-02
P 14= 2.27739E-02
P 15= 2.27739E-02
P 16= 2.27730E-02

FOR RANDOM NUMBER N = 57 P1...PN IS

P 1= 3.73975E-02
P 2= 2.46508E-02
P 3= 1.78421E-02
P 4= 1.72057E-02
P 5= 1.70922E-02
P 6= 1.70438E-02
P 7= 1.70375E-02
P 8= 1.70374E-02
P 9= 1.70368E-02
P 10= 1.70361E-02
P 11= 1.70360E-02
P 12= 1.70352E-02
P 13= 1.70349E-02
P 14= 1.70348E-02
P 15= 1.70346E-02
P 16= 1.70344E-02

P 17= 1.70344E-02
P 18= 1.70344E-02
P 19= 1.70344E-02
P 20= 1.70344E-02
P 21= 1.70344E-02
P 22= 1.70344E-02
P 23= 1.70344E-02
P 24= 1.70344E-02
P 25= 1.70344E-02
P 26= 1.70344E-02
P 27= 1.70344E-02
P 28= 1.70344E-02
P 29= 1.70344E-02
P 30= 1.70344E-02
P 31= 1.70344E-02
P 32= 1.70344E-02
P 33= 1.70344E-02
P 34= 1.70344E-02
P 35= 1.70344E-02
P 36= 1.70344E-02
P 37= 1.70344E-02
P 38= 1.70344E-02
P 39= 1.70344E-02
P 40= 1.70344E-02
P 41= 1.70344E-02
P 42= 1.70344E-02
P 43= 1.70344E-02
P 44= 1.70344E-02
P 45= 1.70344E-02
P 46= 1.70344E-02
P 47= 1.70344E-02
P 48= 1.70344E-02
P 49= 1.70344E-02
P 50= 1.70344E-02
P 51= 1.70344E-02
P 52= 1.70344E-02
P 53= 1.70344E-02
P 54= 1.70344E-02
P 55= 1.70344E-02
P 56= 1.70344E-02
P 57= 1.70344E-02

FOR RANDOM NUMBER N = 94 P1...PN IS

P 1= 1.42644E-02
P 2= 1.26416E-02

P 3= 1.19530E-02
P 4= 1.13587E-02
P 5= 1.06787E-02
P 6= 1.06497E-02
P 7= 1.06472E-02
P 8= 1.05979E-02
P 9= 1.05886E-02
P 10= 1.05499E-02
P 11= 1.05487E-02
P 12= 1.05485E-02
P 13= 1.05485E-02
P 14= 1.05485E-02
P 15= 1.05485E-02
P 16= 1.05485E-02
P 17= 1.05485E-02
P 18= 1.05485E-02
P 19= 1.05485E-02
P 20= 1.05485E-02
P 21= 1.05485E-02
P 22= 1.05485E-02
P 23= 1.05485E-02
P 24= 1.05485E-02
P 25= 1.05485E-02
P 26= 1.05485E-02
P 27= 1.05485E-02
P 28= 1.05485E-02
P 29= 1.05485E-02
P 30= 1.05485E-02
P 31= 1.05485E-02
P 32= 1.05485E-02
P 33= 1.05485E-02
P 34= 1.05485E-02
P 35= 1.05485E-02
P 36= 1.05485E-02
P 37= 1.05485E-02
P 38= 1.05485E-02
P 39= 1.05485E-02
P 40= 1.05485E-02
P 41= 1.05485E-02
P 42= 1.05485E-02
P 43= 1.05485E-02
P 44= 1.05485E-02
P 45= 1.05485E-02
P 46= 1.05485E-02
P 47= 1.05485E-02
P 48= 1.05485E-02

P 49= 1.05485E-02
P 50= 1.05485E-02
P 51= 1.05485E-02
P 52= 1.05485E-02
P 53= 1.05485E-02
P 54= 1.05485E-02
P 55= 1.05485E-02
P 56= 1.05485E-02
P 57= 1.05485E-02
P 58= 1.05485E-02
P 59= 1.05484E-02
P 60= 1.05484E-02
P 61= 1.05484E-02
P 62= 1.05484E-02
P 63= 1.05484E-02
P 64= 1.05484E-02
P 65= 1.05484E-02
P 66= 1.05484E-02
P 67= 1.05484E-02
P 68= 1.05484E-02
P 69= 1.05484E-02
P 70= 1.05484E-02
P 71= 1.05484E-02
P 72= 1.05484E-02
P 73= 1.05484E-02
P 74= 1.05484E-02
P 75= 1.05484E-02
P 76= 1.05484E-02
P 77= 1.05484E-02
P 78= 1.05484E-02
P 79= 1.05484E-02
P 80= 1.05484E-02
P 81= 1.05484E-02
P 82= 1.05484E-02
P 83= 1.05484E-02
P 84= 1.05484E-02
P 85= 1.05484E-02
P 86= 1.05484E-02
P 87= 1.05484E-02
P 88= 1.05484E-02
P 89= 1.05484E-02
P 90= 1.05484E-02
P 91= 1.05484E-02
P 92= 1.05484E-02
P 93= 1.05484E-02
P 94= 1.05484E-02

FOR RANDOM NUMBER N = 95 P1...PN IS

P 1= 2.16930E-02
P 2= 2.04488E-02
P 3= 1.42240E-02
P 4= 1.16203E-02
P 5= 1.06512E-02
P 6= 1.05671E-02
P 7= 1.02590E-02
P 8= 1.02440E-02
P 9= 1.02371E-02
P 10= 1.02370E-02
P 11= 1.02348E-02
P 12= 1.02333E-02
P 13= 1.02332E-02
P 14= 1.02332E-02
P 15= 1.02331E-02
P 16= 1.02331E-02
P 17= 1.02331E-02
P 18= 1.02331E-02
P 19= 1.02331E-02
P 20= 1.02331E-02
P 21= 1.02331E-02
P 22= 1.02331E-02
P 23= 1.02331E-02
P 24= 1.02331E-02
P 25= 1.02331E-02
P 26= 1.02331E-02
P 27= 1.02331E-02
P 28= 1.02331E-02
P 29= 1.02331E-02
P 30= 1.02331E-02
P 31= 1.02331E-02
P 32= 1.02331E-02
P 33= 1.02331E-02
P 34= 1.02331E-02
P 35= 1.02331E-02
P 36= 1.02331E-02
P 37= 1.02331E-02
P 38= 1.02331E-02
P 39= 1.02331E-02
P 40= 1.02331E-02
P 41= 1.02331E-02
P 42= 1.02331E-02
P 43= 1.02331E-02

P 44= 1.02331E-02
P 45= 1.02331E-02
P 46= 1.02331E-02
P 47= 1.02331E-02
P 48= 1.02331E-02
P 49= 1.02331E-02
P 50= 1.02331E-02
P 51= 1.02331E-02
P 52= 1.02331E-02
P 53= 1.02331E-02
P 54= 1.02331E-02
P 55= 1.02331E-02
P 56= 1.02331E-02
P 57= 1.02331E-02
P 58= 1.02331E-02
P 59= 1.02331E-02
P 60= 1.02331E-02
P 61= 1.02331E-02
P 62= 1.02331E-02
P 63= 1.02331E-02
P 64= 1.02331E-02
P 65= 1.02331E-02
P 66= 1.02331E-02
P 67= 1.02331E-02
P 68= 1.02331E-02
P 69= 1.02331E-02
P 70= 1.02331E-02
P 71= 1.02331E-02
P 72= 1.02331E-02
P 73= 1.02331E-02
P 74= 1.02331E-02
P 75= 1.02331E-02
P 76= 1.02331E-02
P 77= 1.02331E-02
P 78= 1.02331E-02
P 79= 1.02331E-02
P 80= 1.02331E-02
P 81= 1.02331E-02
P 82= 1.02331E-02
P 83= 1.02331E-02
P 84= 1.02331E-02
P 85= 1.02331E-02
P 86= 1.02331E-02
P 87= 1.02331E-02
P 88= 1.02331E-02
P 89= 1.02331E-02

P 90= 1.02331E-02
P 91= 1.02331E-02
P 92= 1.02331E-02
P 93= 1.02331E-02
P 94= 1.02331E-02
P 95= 1.02332E-02

FOR RANDOM NUMBER N = 74 P1...PN IS

P 1= 1.01838E-01
P 2= 7.55673E-02
P 3= 4.30418E-02
P 4= 1.25112E-02
P 5= 1.14401E-02
P 6= 1.10503E-02
P 7= 1.10176E-02
P 8= 1.09944E-02
P 9= 1.09865E-02
P 10= 1.09475E-02
P 11= 1.09472E-02
P 12= 1.09470E-02
P 13= 1.09470E-02
P 14= 1.09470E-02
P 15= 1.09470E-02
P 16= 1.09470E-02
P 17= 1.09470E-02
P 18= 1.09470E-02
P 19= 1.09470E-02
P 20= 1.09470E-02
P 21= 1.09470E-02
P 22= 1.09470E-02
P 23= 1.09470E-02
P 24= 1.09470E-02
P 25= 1.09470E-02
P 26= 1.09470E-02
P 27= 1.09470E-02
P 28= 1.09470E-02
P 29= 1.09470E-02
P 30= 1.09470E-02
P 31= 1.09470E-02
P 32= 1.09470E-02
P 33= 1.09470E-02
P 34= 1.09470E-02
P 35= 1.09470E-02
P 36= 1.09470E-02
P 37= 1.09470E-02

P 38= 1.09470E-02
P 39= 1.09470E-02
P 40= 1.09470E-02
P 41= 1.09470E-02
P 42= 1.09470E-02
P 43= 1.09470E-02
P 44= 1.09470E-02
P 45= 1.09470E-02
P 46= 1.09470E-02
P 47= 1.09470E-02
P 48= 1.09470E-02
P 49= 1.09470E-02
P 50= 1.09470E-02
P 51= 1.09470E-02
P 52= 1.09470E-02
P 53= 1.09470E-02
P 54= 1.09470E-02
P 55= 1.09470E-02
P 56= 1.09470E-02
P 57= 1.09470E-02
P 58= 1.09470E-02
P 59= 1.09470E-02
P 60= 1.09470E-02
P 61= 1.09470E-02
P 62= 1.09470E-02
P 63= 1.09470E-02
P 64= 1.09470E-02
P 65= 1.09470E-02
P 66= 1.09470E-02
P 67= 1.09470E-02
P 68= 1.09470E-02
P 69= 1.09470E-02
P 70= 1.09470E-02
P 71= 1.09470E-02
P 72= 1.09470E-02
P 73= 1.09469E-02
P 74= 1.09469E-02

FOR RANDOM NUMBER N = 53 P1...PN IS

P 1= 5.65010E-02
P 2= 4.99140E-02
P 3= 2.76768E-02
P 4= 1.93769E-02
P 5= 1.77594E-02
P 6= 1.76805E-02

P 7= 1.72590E-02
P 8= 1.72575E-02
P 9= 1.72574E-02
P 10= 1.72574E-02
P 11= 1.72573E-02
P 12= 1.72572E-02
P 13= 1.72572E-02
P 14= 1.72572E-02
P 15= 1.72572E-02
P 16= 1.72572E-02
P 17= 1.72572E-02
P 18= 1.72572E-02
P 19= 1.72572E-02
P 20= 1.72572E-02
P 21= 1.72572E-02
P 22= 1.72572E-02
P 23= 1.72572E-02
P 24= 1.72572E-02
P 25= 1.72572E-02
P 26= 1.72572E-02
P 27= 1.72572E-02
P 28= 1.72572E-02
P 29= 1.72572E-02
P 30= 1.72572E-02
P 31= 1.72572E-02
P 32= 1.72572E-02
P 33= 1.72572E-02
P 34= 1.72572E-02
P 35= 1.72572E-02
P 36= 1.72572E-02
P 37= 1.72572E-02
P 38= 1.72572E-02
P 39= 1.72572E-02
P 40= 1.72572E-02
P 41= 1.72572E-02
P 42= 1.72572E-02
P 43= 1.72572E-02
P 44= 1.72572E-02
P 45= 1.72572E-02
P 46= 1.72572E-02
P 47= 1.72572E-02
P 48= 1.72572E-02
P 49= 1.72572E-02
P 50= 1.72572E-02
P 51= 1.72572E-02
P 52= 1.72572E-02

P 53= 1.72572E-02

FOR RANDOM NUMBER N = 87 P1...PN IS

P 1= 0.145458
P 2= 2.21831E-02
P 3= 1.37726E-02
P 4= 1.28589E-02
P 5= 9.87722E-03
P 6= 9.74205E-03
P 7= 9.74081E-03
P 8= 9.72443E-03
P 9= 9.71690E-03
P 10= 9.70668E-03
P 11= 9.70559E-03
P 12= 9.70481E-03
P 13= 9.70433E-03
P 14= 9.70421E-03
P 15= 9.70421E-03
P 16= 9.70421E-03
P 17= 9.70417E-03
P 18= 9.70413E-03
P 19= 9.70412E-03
P 20= 9.70412E-03
P 21= 9.70412E-03
P 22= 9.70412E-03
P 23= 9.70411E-03
P 24= 9.70411E-03
P 25= 9.70411E-03
P 26= 9.70411E-03
P 27= 9.70411E-03
P 28= 9.70411E-03
P 29= 9.70411E-03
P 30= 9.70411E-03
P 31= 9.70411E-03
P 32= 9.70411E-03
P 33= 9.70411E-03
P 34= 9.70411E-03
P 35= 9.70411E-03
P 36= 9.70411E-03
P 37= 9.70411E-03
P 38= 9.70411E-03
P 39= 9.70411E-03
P 40= 9.70411E-03
P 41= 9.70411E-03
P 42= 9.70411E-03

P 43= 9.70411E-03
P 44= 9.70411E-03
P 45= 9.70411E-03
P 46= 9.70411E-03
P 47= 9.70411E-03
P 48= 9.70411E-03
P 49= 9.70411E-03
P 50= 9.70411E-03
P 51= 9.70411E-03
P 52= 9.70411E-03
P 53= 9.70411E-03
P 54= 9.70411E-03
P 55= 9.70411E-03
P 56= 9.70411E-03
P 57= 9.70411E-03
P 58= 9.70411E-03
P 59= 9.70411E-03
P 60= 9.70411E-03
P 61= 9.70411E-03
P 62= 9.70411E-03
P 63= 9.70412E-03
P 64= 9.70412E-03
P 65= 9.70412E-03
P 66= 9.70412E-03
P 67= 9.70412E-03
P 68= 9.70412E-03
P 69= 9.70412E-03
P 70= 9.70412E-03
P 71= 9.70412E-03
P 72= 9.70412E-03
P 73= 9.70412E-03
P 74= 9.70412E-03
P 75= 9.70412E-03
P 76= 9.70412E-03
P 77= 9.70412E-03
P 78= 9.70412E-03
P 79= 9.70412E-03
P 80= 9.70412E-03
P 81= 9.70412E-03
P 82= 9.70412E-03
P 83= 9.70412E-03
P 84= 9.70413E-03
P 85= 9.70413E-03
P 86= 9.70414E-03
P 87= 9.70415E-03

FOR RANDOM NUMBER N = 42 P1...PN IS

P 1= 0.140679
P 2= 0.137035
P 3= 6.09931E-02
P 4= 4.19151E-02
P 5= 2.97490E-02
P 6= 1.77875E-02
P 7= 1.63063E-02
P 8= 1.58773E-02
P 9= 1.58763E-02
P 10= 1.58752E-02
P 11= 1.58724E-02
P 12= 1.58723E-02
P 13= 1.58722E-02
P 14= 1.58721E-02
P 15= 1.58721E-02
P 16= 1.58720E-02
P 17= 1.58720E-02
P 18= 1.58720E-02
P 19= 1.58720E-02
P 20= 1.58720E-02
P 21= 1.58720E-02
P 22= 1.58720E-02
P 23= 1.58720E-02
P 24= 1.58720E-02
P 25= 1.58720E-02
P 26= 1.58720E-02
P 27= 1.58720E-02
P 28= 1.58720E-02
P 29= 1.58720E-02
P 30= 1.58720E-02
P 31= 1.58720E-02
P 32= 1.58720E-02
P 33= 1.58720E-02
P 34= 1.58720E-02
P 35= 1.58720E-02
P 36= 1.58720E-02
P 37= 1.58720E-02
P 38= 1.58720E-02
P 39= 1.58720E-02
P 40= 1.58720E-02
P 41= 1.58720E-02
P 42= 1.58720E-02

FOR RANDOM NUMBER N = 89 P1...PN IS

P 1= 5.08135E-02
P 2= 2.02120E-02
P 3= 1.13020E-02
P 4= 1.08717E-02
P 5= 1.07296E-02
P 6= 1.07008E-02
P 7= 1.06879E-02
P 8= 1.06766E-02
P 9= 1.06749E-02
P 10= 1.06713E-02
P 11= 1.06691E-02
P 12= 1.06673E-02
P 13= 1.06667E-02
P 14= 1.06666E-02
P 15= 1.06666E-02
P 16= 1.06665E-02
P 17= 1.06665E-02
P 18= 1.06665E-02
P 19= 1.06665E-02
P 20= 1.06665E-02
P 21= 1.06665E-02
P 22= 1.06665E-02
P 23= 1.06665E-02
P 24= 1.06665E-02
P 25= 1.06665E-02
P 26= 1.06665E-02
P 27= 1.06665E-02
P 28= 1.06665E-02
P 29= 1.06665E-02
P 30= 1.06665E-02
P 31= 1.06665E-02
P 32= 1.06665E-02
P 33= 1.06665E-02
P 34= 1.06665E-02
P 35= 1.06665E-02
P 36= 1.06665E-02
P 37= 1.06665E-02
P 38= 1.06665E-02
P 39= 1.06665E-02
P 40= 1.06665E-02
P 41= 1.06665E-02
P 42= 1.06665E-02
P 43= 1.06665E-02
P 44= 1.06665E-02
P 45= 1.06665E-02

P 46= 1.06665E-02
P 47= 1.06665E-02
P 48= 1.06665E-02
P 49= 1.06665E-02
P 50= 1.06665E-02
P 51= 1.06665E-02
P 52= 1.06665E-02
P 53= 1.06665E-02
P 54= 1.06665E-02
P 55= 1.06665E-02
P 56= 1.06665E-02
P 57= 1.06665E-02
P 58= 1.06665E-02
P 59= 1.06665E-02
P 60= 1.06665E-02
P 61= 1.06665E-02
P 62= 1.06665E-02
P 63= 1.06665E-02
P 64= 1.06665E-02
P 65= 1.06665E-02
P 66= 1.06665E-02
P 67= 1.06665E-02
P 68= 1.06665E-02
P 69= 1.06665E-02
P 70= 1.06665E-02
P 71= 1.06665E-02
P 72= 1.06665E-02
P 73= 1.06665E-02
P 74= 1.06665E-02
P 75= 1.06665E-02
P 76= 1.06665E-02
P 77= 1.06665E-02
P 78= 1.06665E-02
P 79= 1.06665E-02
P 80= 1.06665E-02
P 81= 1.06665E-02
P 82= 1.06665E-02
P 83= 1.06665E-02
P 84= 1.06665E-02
P 85= 1.06665E-02
P 86= 1.06665E-02
P 87= 1.06665E-02
P 88= 1.06665E-02
P 89= 1.06665E-02

FOR RANDOM NUMBER N = 76 P1...PN IS

P 1= 0.978158
P 2= 1.41136E-02
P 3= 1.93435E-03
P 4= 5.36681E-04
P 5= 2.37108E-04
P 6= 2.12946E-04
P 7= 1.62584E-04
P 8= 1.12023E-04
P 9= 8.04150E-05
P 10= 7.76911E-05
P 11= 7.69028E-05
P 12= 6.83643E-05
P 13= 6.69410E-05
P 14= 6.62944E-05
P 15= 6.61122E-05
P 16= 6.61103E-05
P 17= 6.60975E-05
P 18= 6.60909E-05
P 19= 6.60892E-05
P 20= 6.60770E-05
P 21= 6.60746E-05
P 22= 6.60713E-05
P 23= 6.60713E-05
P 24= 6.60712E-05
P 25= 6.60713E-05
P 26= 6.60715E-05
P 27= 6.60716E-05
P 28= 6.60728E-05
P 29= 6.60729E-05
P 30= 6.60726E-05
P 31= 6.60725E-05
P 32= 6.60718E-05
P 33= 6.60704E-05
P 34= 6.60705E-05
P 35= 6.60716E-05
P 36= 6.60719E-05
P 37= 6.60718E-05
P 38= 6.60717E-05
P 39= 6.60717E-05
P 40= 6.60723E-05
P 41= 6.60722E-05
P 42= 6.60721E-05
P 43= 6.60720E-05
P 44= 6.60718E-05
P 45= 6.60710E-05

P 46= 6.60706E-05
P 47= 6.60702E-05
P 48= 6.60706E-05
P 49= 6.60708E-05
P 50= 6.60723E-05
P 51= 6.60722E-05
P 52= 6.60721E-05
P 53= 6.60705E-05
P 54= 6.60705E-05
P 55= 6.60709E-05
P 56= 6.60724E-05
P 57= 6.60723E-05
P 58= 6.60721E-05
P 59= 6.60705E-05
P 60= 6.60701E-05
P 61= 6.60711E-05
P 62= 6.60731E-05
P 63= 6.60729E-05
P 64= 6.60707E-05
P 65= 6.60712E-05
P 66= 6.60737E-05
P 67= 6.60726E-05
P 68= 6.60713E-05
P 69= 6.60715E-05
P 70= 6.60737E-05
P 71= 6.60727E-05
P 72= 6.60667E-05
P 73= 6.60677E-05
P 74= 6.60733E-05
P 75= 6.60732E-05
P 76= 6.60419E-05

FOR RANDOM NUMBER N = 74 P1...PN IS

P 1= 4.86513E-02
P 2= 1.31219E-02
P 3= 1.30571E-02
P 4= 1.30454E-02
P 5= 1.30320E-02
P 6= 1.30316E-02
P 7= 1.30316E-02
P 8= 1.30305E-02
P 9= 1.30304E-02
P 10= 1.30304E-02
P 11= 1.30303E-02
P 12= 1.30303E-02

P 13= 1.30303E-02
P 14= 1.30303E-02
P 15= 1.30303E-02
P 16= 1.30303E-02
P 17= 1.30303E-02
P 18= 1.30303E-02
P 19= 1.30303E-02
P 20= 1.30303E-02
P 21= 1.30303E-02
P 22= 1.30303E-02
P 23= 1.30303E-02
P 24= 1.30303E-02
P 25= 1.30303E-02
P 26= 1.30303E-02
P 27= 1.30303E-02
P 28= 1.30303E-02
P 29= 1.30303E-02
P 30= 1.30303E-02
P 31= 1.30303E-02
P 32= 1.30303E-02
P 33= 1.30303E-02
P 34= 1.30303E-02
P 35= 1.30303E-02
P 36= 1.30303E-02
P 37= 1.30303E-02
P 38= 1.30303E-02
P 39= 1.30303E-02
P 40= 1.30303E-02
P 41= 1.30303E-02
P 42= 1.30303E-02
P 43= 1.30303E-02
P 44= 1.30303E-02
P 45= 1.30303E-02
P 46= 1.30303E-02
P 47= 1.30303E-02
P 48= 1.30303E-02
P 49= 1.30303E-02
P 50= 1.30303E-02
P 51= 1.30303E-02
P 52= 1.30303E-02
P 53= 1.30303E-02
P 54= 1.30303E-02
P 55= 1.30303E-02
P 56= 1.30303E-02
P 57= 1.30303E-02
P 58= 1.30303E-02

P 59= 1.30303E-02
P 60= 1.30303E-02
P 61= 1.30303E-02
P 62= 1.30303E-02
P 63= 1.30303E-02
P 64= 1.30303E-02
P 65= 1.30303E-02
P 66= 1.30303E-02
P 67= 1.30303E-02
P 68= 1.30303E-02
P 69= 1.30303E-02
P 70= 1.30303E-02
P 71= 1.30303E-02
P 72= 1.30303E-02
P 73= 1.30303E-02
P 74= 1.30302E-02

FOR RANDOM NUMBER N = 80 P1...PN IS

P 1= 5.00937E-02
P 2= 1.54066E-02
P 3= 1.29448E-02
P 4= 1.26347E-02
P 5= 1.26193E-02
P 6= 1.23782E-02
P 7= 1.21067E-02
P 8= 1.19608E-02
P 9= 1.19527E-02
P 10= 1.19454E-02
P 11= 1.19438E-02
P 12= 1.19426E-02
P 13= 1.19423E-02
P 14= 1.19422E-02
P 15= 1.19422E-02
P 16= 1.19422E-02
P 17= 1.19422E-02
P 18= 1.19422E-02
P 19= 1.19422E-02
P 20= 1.19422E-02
P 21= 1.19422E-02
P 22= 1.19422E-02
P 23= 1.19422E-02
P 24= 1.19422E-02
P 25= 1.19422E-02
P 26= 1.19422E-02
P 27= 1.19422E-02

P 28=	1.19422E-02
P 29=	1.19422E-02
P 30=	1.19422E-02
P 31=	1.19422E-02
P 32=	1.19422E-02
P 33=	1.19422E-02
P 34=	1.19422E-02
P 35=	1.19422E-02
P 36=	1.19422E-02
P 37=	1.19422E-02
P 38=	1.19422E-02
P 39=	1.19422E-02
P 40=	1.19422E-02
P 41=	1.19422E-02
P 42=	1.19422E-02
P 43=	1.19422E-02
P 44=	1.19422E-02
P 45=	1.19422E-02
P 46=	1.19422E-02
P 47=	1.19422E-02
P 48=	1.19422E-02
P 49=	1.19422E-02
P 50=	1.19422E-02
P 51=	1.19422E-02
P 52=	1.19422E-02
P 53=	1.19422E-02
P 54=	1.19422E-02
P 55=	1.19422E-02
P 56=	1.19422E-02
P 57=	1.19422E-02
P 58=	1.19422E-02
P 59=	1.19422E-02
P 60=	1.19422E-02
P 61=	1.19422E-02
P 62=	1.19422E-02
P 63=	1.19422E-02
P 64=	1.19422E-02
P 65=	1.19422E-02
P 66=	1.19422E-02
P 67=	1.19422E-02
P 68=	1.19422E-02
P 69=	1.19422E-02
P 70=	1.19422E-02
P 71=	1.19422E-02
P 72=	1.19422E-02
P 73=	1.19422E-02

P 74= 1.19422E-02
P 75= 1.19422E-02
P 76= 1.19422E-02
P 77= 1.19422E-02
P 78= 1.19422E-02
P 79= 1.19422E-02
P 80= 1.19422E-02

FOR RANDOM NUMBER N = 53 P1...PN IS

P 1= 3.33913E-02
P 2= 3.02880E-02
P 3= 2.43567E-02
P 4= 2.20583E-02
P 5= 1.93142E-02
P 6= 1.86549E-02
P 7= 1.82607E-02
P 8= 1.82288E-02
P 9= 1.81620E-02
P 10= 1.81444E-02
P 11= 1.81229E-02
P 12= 1.81203E-02
P 13= 1.81201E-02
P 14= 1.81197E-02
P 15= 1.81197E-02
P 16= 1.81195E-02
P 17= 1.81195E-02
P 18= 1.81194E-02
P 19= 1.81194E-02
P 20= 1.81194E-02
P 21= 1.81194E-02
P 22= 1.81194E-02
P 23= 1.81194E-02
P 24= 1.81194E-02
P 25= 1.81194E-02
P 26= 1.81194E-02
P 27= 1.81194E-02
P 28= 1.81194E-02
P 29= 1.81194E-02
P 30= 1.81194E-02
P 31= 1.81194E-02
P 32= 1.81194E-02
P 33= 1.81194E-02
P 34= 1.81194E-02
P 35= 1.81194E-02
P 36= 1.81194E-02

P 37= 1.81194E-02
P 38= 1.81194E-02
P 39= 1.81194E-02
P 40= 1.81194E-02
P 41= 1.81194E-02
P 42= 1.81194E-02
P 43= 1.81194E-02
P 44= 1.81194E-02
P 45= 1.81194E-02
P 46= 1.81194E-02
P 47= 1.81194E-02
P 48= 1.81194E-02
P 49= 1.81194E-02
P 50= 1.81194E-02
P 51= 1.81194E-02
P 52= 1.81194E-02
P 53= 1.81194E-02

FOR RANDOM NUMBER N = 79 P1...PN IS

P 1= 0.124540
P 2= 5.90198E-02
P 3= 3.43237E-02
P 4= 1.83561E-02
P 5= 1.29943E-02
P 6= 1.09072E-02
P 7= 1.03897E-02
P 8= 1.03312E-02
P 9= 1.01998E-02
P 10= 1.01348E-02
P 11= 1.01341E-02
P 12= 1.01284E-02
P 13= 1.01280E-02
P 14= 1.01279E-02
P 15= 1.01276E-02
P 16= 1.01276E-02
P 17= 1.01275E-02
P 18= 1.01275E-02
P 19= 1.01275E-02
P 20= 1.01275E-02
P 21= 1.01275E-02
P 22= 1.01275E-02
P 23= 1.01275E-02
P 24= 1.01275E-02
P 25= 1.01275E-02
P 26= 1.01275E-02

P 27=	1.01275E-02
P 28=	1.01275E-02
P 29=	1.01275E-02
P 30=	1.01275E-02
P 31=	1.01275E-02
P 32=	1.01275E-02
P 33=	1.01275E-02
P 34=	1.01275E-02
P 35=	1.01275E-02
P 36=	1.01275E-02
P 37=	1.01275E-02
P 38=	1.01275E-02
P 39=	1.01275E-02
P 40=	1.01275E-02
P 41=	1.01275E-02
P 42=	1.01275E-02
P 43=	1.01275E-02
P 44=	1.01275E-02
P 45=	1.01275E-02
P 46=	1.01275E-02
P 47=	1.01275E-02
P 48=	1.01275E-02
P 49=	1.01275E-02
P 50=	1.01275E-02
P 51=	1.01275E-02
P 52=	1.01275E-02
P 53=	1.01275E-02
P 54=	1.01275E-02
P 55=	1.01275E-02
P 56=	1.01275E-02
P 57=	1.01275E-02
P 58=	1.01275E-02
P 59=	1.01275E-02
P 60=	1.01275E-02
P 61=	1.01275E-02
P 62=	1.01275E-02
P 63=	1.01275E-02
P 64=	1.01275E-02
P 65=	1.01275E-02
P 66=	1.01275E-02
P 67=	1.01275E-02
P 68=	1.01275E-02
P 69=	1.01275E-02
P 70=	1.01275E-02
P 71=	1.01275E-02
P 72=	1.01275E-02

P 73= 1.01275E-02
P 74= 1.01275E-02
P 75= 1.01274E-02
P 76= 1.01275E-02
P 77= 1.01275E-02
P 78= 1.01275E-02
P 79= 1.01274E-02

FOR RANDOM NUMBER N = 92 P1...PN IS

P 1= 4.78272E-02
P 2= 4.13878E-02
P 3= 3.07037E-02
P 4= 2.80769E-02
P 5= 1.70719E-02
P 6= 9.86764E-03
P 7= 9.79932E-03
P 8= 9.62008E-03
P 9= 9.61483E-03
P 10= 9.60809E-03
P 11= 9.60214E-03
P 12= 9.59894E-03
P 13= 9.59791E-03
P 14= 9.59147E-03
P 15= 9.59138E-03
P 16= 9.59074E-03
P 17= 9.59021E-03
P 18= 9.59021E-03
P 19= 9.59020E-03
P 20= 9.59018E-03
P 21= 9.59016E-03
P 22= 9.59013E-03
P 23= 9.59013E-03
P 24= 9.59013E-03
P 25= 9.59013E-03
P 26= 9.59013E-03
P 27= 9.59013E-03
P 28= 9.59013E-03
P 29= 9.59013E-03
P 30= 9.59013E-03
P 31= 9.59013E-03
P 32= 9.59013E-03
P 33= 9.59013E-03
P 34= 9.59013E-03
P 35= 9.59013E-03
P 36= 9.59013E-03

P 37= 9.59013E-03
P 38= 9.59013E-03
P 39= 9.59013E-03
P 40= 9.59013E-03
P 41= 9.59013E-03
P 42= 9.59013E-03
P 43= 9.59013E-03
P 44= 9.59013E-03
P 45= 9.59013E-03
P 46= 9.59013E-03
P 47= 9.59013E-03
P 48= 9.59012E-03
P 49= 9.59012E-03
P 50= 9.59012E-03
P 51= 9.59012E-03
P 52= 9.59012E-03
P 53= 9.59012E-03
P 54= 9.59012E-03
P 55= 9.59012E-03
P 56= 9.59012E-03
P 57= 9.59012E-03
P 58= 9.59012E-03
P 59= 9.59012E-03
P 60= 9.59012E-03
P 61= 9.59012E-03
P 62= 9.59012E-03
P 63= 9.59012E-03
P 64= 9.59012E-03
P 65= 9.59012E-03
P 66= 9.59012E-03
P 67= 9.59012E-03
P 68= 9.59012E-03
P 69= 9.59012E-03
P 70= 9.59012E-03
P 71= 9.59012E-03
P 72= 9.59012E-03
P 73= 9.59012E-03
P 74= 9.59012E-03
P 75= 9.59012E-03
P 76= 9.59012E-03
P 77= 9.59012E-03
P 78= 9.59012E-03
P 79= 9.59012E-03
P 80= 9.59011E-03
P 81= 9.59011E-03
P 82= 9.59012E-03

P 83= 9.59012E-03
P 84= 9.59012E-03
P 85= 9.59012E-03
P 86= 9.59012E-03
P 87= 9.59012E-03
P 88= 9.59012E-03
P 89= 9.59012E-03
P 90= 9.59012E-03
P 91= 9.59012E-03
P 92= 9.59009E-03

FOR RANDOM NUMBER N = 73 P1...PN IS

P 1= 7.32091E-02
P 2= 3.00890E-02
P 3= 1.47610E-02
P 4= 1.30652E-02
P 5= 1.29528E-02
P 6= 1.29063E-02
P 7= 1.27484E-02
P 8= 1.27254E-02
P 9= 1.27083E-02
P 10= 1.26774E-02
P 11= 1.26624E-02
P 12= 1.26152E-02
P 13= 1.25875E-02
P 14= 1.25860E-02
P 15= 1.25813E-02
P 16= 1.25766E-02
P 17= 1.25714E-02
P 18= 1.25710E-02
P 19= 1.25710E-02
P 20= 1.25710E-02
P 21= 1.25710E-02
P 22= 1.25710E-02
P 23= 1.25710E-02
P 24= 1.25710E-02
P 25= 1.25710E-02
P 26= 1.25710E-02
P 27= 1.25710E-02
P 28= 1.25710E-02
P 29= 1.25710E-02
P 30= 1.25710E-02
P 31= 1.25710E-02
P 32= 1.25710E-02
P 33= 1.25710E-02

P 34= 1.25710E-02
P 35= 1.25710E-02
P 36= 1.25710E-02
P 37= 1.25710E-02
P 38= 1.25710E-02
P 39= 1.25710E-02
P 40= 1.25710E-02
P 41= 1.25710E-02
P 42= 1.25710E-02
P 43= 1.25710E-02
P 44= 1.25710E-02
P 45= 1.25710E-02
P 46= 1.25710E-02
P 47= 1.25710E-02
P 48= 1.25710E-02
P 49= 1.25710E-02
P 50= 1.25710E-02
P 51= 1.25710E-02
P 52= 1.25710E-02
P 53= 1.25710E-02
P 54= 1.25710E-02
P 55= 1.25710E-02
P 56= 1.25710E-02
P 57= 1.25710E-02
P 58= 1.25710E-02
P 59= 1.25710E-02
P 60= 1.25710E-02
P 61= 1.25710E-02
P 62= 1.25710E-02
P 63= 1.25710E-02
P 64= 1.25710E-02
P 65= 1.25710E-02
P 66= 1.25710E-02
P 67= 1.25710E-02
P 68= 1.25710E-02
P 69= 1.25710E-02
P 70= 1.25710E-02
P 71= 1.25710E-02
P 72= 1.25710E-02
P 73= 1.25710E-02

FOR RANDOM NUMBER N = 3 P1...PN IS

P 1= 0.908568
P 2= 8.93507E-02
P 3= 2.08104E-03

FOR RANDOM NUMBER N = 70 P1...PN IS

P 1= 1.03814E-01
P 2= 1.59417E-02
P 3= 1.44818E-02
P 4= 1.32814E-02
P 5= 1.31829E-02
P 6= 1.29136E-02
P 7= 1.29131E-02
P 8= 1.29124E-02
P 9= 1.29123E-02
P 10= 1.29123E-02
P 11= 1.29123E-02
P 12= 1.29123E-02
P 13= 1.29122E-02
P 14= 1.29122E-02
P 15= 1.29122E-02
P 16= 1.29122E-02
P 17= 1.29122E-02
P 18= 1.29122E-02
P 19= 1.29122E-02
P 20= 1.29122E-02
P 21= 1.29122E-02
P 22= 1.29122E-02
P 23= 1.29122E-02
P 24= 1.29122E-02
P 25= 1.29122E-02
P 26= 1.29122E-02
P 27= 1.29122E-02
P 28= 1.29122E-02
P 29= 1.29122E-02
P 30= 1.29122E-02
P 31= 1.29122E-02
P 32= 1.29122E-02
P 33= 1.29122E-02
P 34= 1.29122E-02
P 35= 1.29122E-02
P 36= 1.29122E-02
P 37= 1.29122E-02
P 38= 1.29122E-02
P 39= 1.29122E-02
P 40= 1.29122E-02
P 41= 1.29122E-02
P 42= 1.29122E-02
P 43= 1.29122E-02

P 44= 1.29122E-02
P 45= 1.29122E-02
P 46= 1.29122E-02
P 47= 1.29122E-02
P 48= 1.29122E-02
P 49= 1.29122E-02
P 50= 1.29122E-02
P 51= 1.29122E-02
P 52= 1.29122E-02
P 53= 1.29122E-02
P 54= 1.29122E-02
P 55= 1.29122E-02
P 56= 1.29122E-02
P 57= 1.29122E-02
P 58= 1.29122E-02
P 59= 1.29122E-02
P 60= 1.29122E-02
P 61= 1.29122E-02
P 62= 1.29122E-02
P 63= 1.29122E-02
P 64= 1.29122E-02
P 65= 1.29123E-02
P 66= 1.29123E-02
P 67= 1.29122E-02
P 68= 1.29122E-02
P 69= 1.29122E-02
P 70= 1.29123E-02

FOR RANDOM NUMBER N = 4 P1...PN IS

P 1= 0.358221
P 2= 0.299695
P 3= 0.191465
P 4= 0.150620

FOR RANDOM NUMBER N = 21 P1...PN IS

P 1= 5.64184E-02
P 2= 5.55734E-02
P 3= 5.12485E-02
P 4= 4.93881E-02
P 5= 4.87920E-02
P 6= 4.87299E-02
P 7= 4.74849E-02
P 8= 4.64671E-02
P 9= 4.62466E-02

P 10= 4.60569E-02
P 11= 4.59136E-02
P 12= 4.58749E-02
P 13= 4.57928E-02
P 14= 4.57612E-02
P 15= 4.57521E-02
P 16= 4.57506E-02
P 17= 4.57505E-02
P 18= 4.57502E-02
P 19= 4.57496E-02
P 20= 4.57495E-02
P 21= 4.57493E-02

FOR RANDOM NUMBER N = 88 P1...PN IS

P 1= 1.28051E-02
P 2= 1.23054E-02
P 3= 1.21093E-02
P 4= 1.14652E-02
P 5= 1.13610E-02
P 6= 1.13565E-02
P 7= 1.13253E-02
P 8= 1.13251E-02
P 9= 1.13247E-02
P 10= 1.13245E-02
P 11= 1.13244E-02
P 12= 1.13244E-02
P 13= 1.13243E-02
P 14= 1.13243E-02
P 15= 1.13243E-02
P 16= 1.13243E-02
P 17= 1.13243E-02
P 18= 1.13243E-02
P 19= 1.13243E-02
P 20= 1.13243E-02
P 21= 1.13243E-02
P 22= 1.13243E-02
P 23= 1.13243E-02
P 24= 1.13243E-02
P 25= 1.13243E-02
P 26= 1.13243E-02
P 27= 1.13243E-02
P 28= 1.13243E-02
P 29= 1.13243E-02
P 30= 1.13243E-02
P 31= 1.13243E-02

P 32= 1.13243E-02
P 33= 1.13243E-02
P 34= 1.13243E-02
P 35= 1.13243E-02
P 36= 1.13243E-02
P 37= 1.13243E-02
P 38= 1.13243E-02
P 39= 1.13243E-02
P 40= 1.13243E-02
P 41= 1.13243E-02
P 42= 1.13243E-02
P 43= 1.13243E-02
P 44= 1.13243E-02
P 45= 1.13243E-02
P 46= 1.13243E-02
P 47= 1.13243E-02
P 48= 1.13243E-02
P 49= 1.13243E-02
P 50= 1.13243E-02
P 51= 1.13243E-02
P 52= 1.13243E-02
P 53= 1.13243E-02
P 54= 1.13243E-02
P 55= 1.13243E-02
P 56= 1.13243E-02
P 57= 1.13243E-02
P 58= 1.13243E-02
P 59= 1.13243E-02
P 60= 1.13243E-02
P 61= 1.13243E-02
P 62= 1.13243E-02
P 63= 1.13243E-02
P 64= 1.13243E-02
P 65= 1.13243E-02
P 66= 1.13243E-02
P 67= 1.13243E-02
P 68= 1.13243E-02
P 69= 1.13243E-02
P 70= 1.13243E-02
P 71= 1.13243E-02
P 72= 1.13243E-02
P 73= 1.13243E-02
P 74= 1.13243E-02
P 75= 1.13243E-02
P 76= 1.13243E-02
P 77= 1.13243E-02

P 78= 1.13243E-02
P 79= 1.13243E-02
P 80= 1.13243E-02
P 81= 1.13243E-02
P 82= 1.13243E-02
P 83= 1.13243E-02
P 84= 1.13243E-02
P 85= 1.13243E-02
P 86= 1.13243E-02
P 87= 1.13243E-02
P 88= 1.13243E-02

FOR RANDOM NUMBER N = 8 P1...PN IS

P 1= 0.198163
P 2= 0.165504
P 3= 0.121688
P 4= 0.105357
P 5= 0.105220
P 6= 0.104914
P 7= 1.01216E-01
P 8= 9.79384E-02

FOR RANDOM NUMBER N = 41 P1...PN IS

P 1= 5.66614E-02
P 2= 5.45189E-02
P 3= 2.53006E-02
P 4= 2.47385E-02
P 5= 2.35398E-02
P 6= 2.30509E-02
P 7= 2.30096E-02
P 8= 2.28574E-02
P 9= 2.27827E-02
P 10= 2.27500E-02
P 11= 2.27030E-02
P 12= 2.26788E-02
P 13= 2.26463E-02
P 14= 2.26021E-02
P 15= 2.25987E-02
P 16= 2.25985E-02
P 17= 2.25985E-02
P 18= 2.25985E-02
P 19= 2.25985E-02
P 20= 2.25985E-02
P 21= 2.25985E-02

P 22= 2.25985E-02
P 23= 2.25985E-02
P 24= 2.25985E-02
P 25= 2.25985E-02
P 26= 2.25985E-02
P 27= 2.25985E-02
P 28= 2.25985E-02
P 29= 2.25985E-02
P 30= 2.25985E-02
P 31= 2.25985E-02
P 32= 2.25985E-02
P 33= 2.25985E-02
P 34= 2.25985E-02
P 35= 2.25985E-02
P 36= 2.25985E-02
P 37= 2.25985E-02
P 38= 2.25985E-02
P 39= 2.25985E-02
P 40= 2.25985E-02
P 41= 2.25985E-02

FOR RANDOM NUMBER N = 14 P1...PN IS

P 1= 0.236931
P 2= 0.199444
P 3= 0.186016
P 4= 0.108609
P 5= 4.22082E-02
P 6= 2.98237E-02
P 7= 2.78968E-02
P 8= 2.64523E-02
P 9= 2.43556E-02
P 10= 2.39056E-02
P 11= 2.38901E-02
P 12= 2.38402E-02
P 13= 2.34282E-02
P 14= 2.32001E-02

FOR RANDOM NUMBER N = 91 P1...PN IS

P 1= 0.386527
P 2= 0.229136
P 3= 0.201294
P 4= 0.119449
P 5= 5.81661E-03
P 6= 2.77578E-03

P 7= 1.10669E-03
P 8= 1.00836E-03
P 9= 9.76326E-04
P 10= 8.59411E-04
P 11= 8.30043E-04
P 12= 7.18221E-04
P 13= 6.50822E-04
P 14= 6.31351E-04
P 15= 6.28539E-04
P 16= 6.26969E-04
P 17= 6.26253E-04
P 18= 6.26211E-04
P 19= 6.26204E-04
P 20= 6.26200E-04
P 21= 6.26197E-04
P 22= 6.26196E-04
P 23= 6.26195E-04
P 24= 6.26195E-04
P 25= 6.26195E-04
P 26= 6.26195E-04
P 27= 6.26194E-04
P 28= 6.26194E-04
P 29= 6.26194E-04
P 30= 6.26194E-04
P 31= 6.26194E-04
P 32= 6.26194E-04
P 33= 6.26194E-04
P 34= 6.26193E-04
P 35= 6.26193E-04
P 36= 6.26193E-04
P 37= 6.26193E-04
P 38= 6.26192E-04
P 39= 6.26192E-04
P 40= 6.26192E-04
P 41= 6.26191E-04
P 42= 6.26191E-04
P 43= 6.26191E-04
P 44= 6.26191E-04
P 45= 6.26190E-04
P 46= 6.26190E-04
P 47= 6.26189E-04
P 48= 6.26189E-04
P 49= 6.26188E-04
P 50= 6.26188E-04
P 51= 6.26188E-04
P 52= 6.26188E-04

P 53= 6.26187E-04
P 54= 6.26186E-04
P 55= 6.26186E-04
P 56= 6.26186E-04
P 57= 6.26185E-04
P 58= 6.26184E-04
P 59= 6.26184E-04
P 60= 6.26183E-04
P 61= 6.26182E-04
P 62= 6.26182E-04
P 63= 6.26181E-04
P 64= 6.26180E-04
P 65= 6.26178E-04
P 66= 6.26177E-04
P 67= 6.26177E-04
P 68= 6.26177E-04
P 69= 6.26176E-04
P 70= 6.26176E-04
P 71= 6.26178E-04
P 72= 6.26177E-04
P 73= 6.26176E-04
P 74= 6.26176E-04
P 75= 6.26177E-04
P 76= 6.26177E-04
P 77= 6.26175E-04
P 78= 6.26176E-04
P 79= 6.26177E-04
P 80= 6.26177E-04
P 81= 6.26176E-04
P 82= 6.26176E-04
P 83= 6.26179E-04
P 84= 6.26179E-04
P 85= 6.26176E-04
P 86= 6.26177E-04
P 87= 6.26178E-04
P 88= 6.26177E-04
P 89= 6.26172E-04
P 90= 6.26175E-04
P 91= 6.26199E-04

FOR RANDOM NUMBER N = 61 P1...PN IS

P 1= 0.353112
P 2= 3.31054E-02
P 3= 2.34659E-02
P 4= 2.18361E-02

P 5= 1.53262E-02
P 6= 1.01468E-02
P 7= 1.00587E-02
P 8= 9.98995E-03
P 9= 9.90046E-03
P 10= 9.88143E-03
P 11= 9.87733E-03
P 12= 9.87033E-03
P 13= 9.86827E-03
P 14= 9.86740E-03
P 15= 9.86724E-03
P 16= 9.86593E-03
P 17= 9.86583E-03
P 18= 9.86582E-03
P 19= 9.86580E-03
P 20= 9.86578E-03
P 21= 9.86578E-03
P 22= 9.86578E-03
P 23= 9.86578E-03
P 24= 9.86578E-03
P 25= 9.86578E-03
P 26= 9.86578E-03
P 27= 9.86579E-03
P 28= 9.86579E-03
P 29= 9.86579E-03
P 30= 9.86579E-03
P 31= 9.86579E-03
P 32= 9.86579E-03
P 33= 9.86579E-03
P 34= 9.86579E-03
P 35= 9.86579E-03
P 36= 9.86579E-03
P 37= 9.86579E-03
P 38= 9.86579E-03
P 39= 9.86579E-03
P 40= 9.86579E-03
P 41= 9.86579E-03
P 42= 9.86579E-03
P 43= 9.86579E-03
P 44= 9.86579E-03
P 45= 9.86579E-03
P 46= 9.86579E-03
P 47= 9.86579E-03
P 48= 9.86579E-03
P 49= 9.86579E-03
P 50= 9.86579E-03

P 51= 9.86579E-03
P 52= 9.86579E-03
P 53= 9.86579E-03
P 54= 9.86579E-03
P 55= 9.86579E-03
P 56= 9.86579E-03
P 57= 9.86579E-03
P 58= 9.86579E-03
P 59= 9.86580E-03
P 60= 9.86580E-03
P 61= 9.86576E-03

FOR RANDOM NUMBER N = 85 P1...PN IS

P 1= 0.453875
P 2= 1.27944E-02
P 3= 8.96211E-03
P 4= 7.34706E-03
P 5= 6.97198E-03
P 6= 6.80093E-03
P 7= 6.65758E-03
P 8= 6.39137E-03
P 9= 6.39060E-03
P 10= 6.37553E-03
P 11= 6.36739E-03
P 12= 6.36722E-03
P 13= 6.36596E-03
P 14= 6.36581E-03
P 15= 6.36578E-03
P 16= 6.36575E-03
P 17= 6.36574E-03
P 18= 6.36574E-03
P 19= 6.36574E-03
P 20= 6.36573E-03
P 21= 6.36573E-03
P 22= 6.36573E-03
P 23= 6.36573E-03
P 24= 6.36573E-03
P 25= 6.36574E-03
P 26= 6.36574E-03
P 27= 6.36574E-03
P 28= 6.36574E-03
P 29= 6.36574E-03
P 30= 6.36574E-03
P 31= 6.36574E-03
P 32= 6.36574E-03

P 33= 6.36574E-03
P 34= 6.36574E-03
P 35= 6.36574E-03
P 36= 6.36574E-03
P 37= 6.36574E-03
P 38= 6.36574E-03
P 39= 6.36574E-03
P 40= 6.36574E-03
P 41= 6.36574E-03
P 42= 6.36574E-03
P 43= 6.36574E-03
P 44= 6.36574E-03
P 45= 6.36574E-03
P 46= 6.36574E-03
P 47= 6.36574E-03
P 48= 6.36575E-03
P 49= 6.36575E-03
P 50= 6.36575E-03
P 51= 6.36575E-03
P 52= 6.36574E-03
P 53= 6.36574E-03
P 54= 6.36575E-03
P 55= 6.36575E-03
P 56= 6.36575E-03
P 57= 6.36575E-03
P 58= 6.36575E-03
P 59= 6.36575E-03
P 60= 6.36575E-03
P 61= 6.36575E-03
P 62= 6.36575E-03
P 63= 6.36575E-03
P 64= 6.36574E-03
P 65= 6.36574E-03
P 66= 6.36575E-03
P 67= 6.36575E-03
P 68= 6.36575E-03
P 69= 6.36575E-03
P 70= 6.36575E-03
P 71= 6.36575E-03
P 72= 6.36575E-03
P 73= 6.36575E-03
P 74= 6.36575E-03
P 75= 6.36574E-03
P 76= 6.36575E-03
P 77= 6.36575E-03
P 78= 6.36575E-03

P 79= 6.36575E-03
P 80= 6.36575E-03
P 81= 6.36575E-03
P 82= 6.36575E-03
P 83= 6.36574E-03
P 84= 6.36574E-03
P 85= 6.36576E-03

FOR RANDOM NUMBER N = 14 P1...PN IS

P 1= 0.928489
P 2= 1.26361E-02
P 3= 5.99729E-03
P 4= 5.68428E-03
P 5= 5.22975E-03
P 6= 5.08211E-03
P 7= 4.84462E-03
P 8= 4.61559E-03
P 9= 4.59188E-03
P 10= 4.57231E-03
P 11= 4.56959E-03
P 12= 4.56299E-03
P 13= 4.56207E-03
P 14= 4.56196E-03

FOR RANDOM NUMBER N = 33 P1...PN IS

P 1= 0.253163
P 2= 1.01796E-01
P 3= 9.33055E-02
P 4= 3.40916E-02
P 5= 2.60698E-02
P 6= 2.04475E-02
P 7= 1.89070E-02
P 8= 1.86897E-02
P 9= 1.77407E-02
P 10= 1.77026E-02
P 11= 1.76963E-02
P 12= 1.76859E-02
P 13= 1.73053E-02
P 14= 1.72783E-02
P 15= 1.72720E-02
P 16= 1.72712E-02
P 17= 1.72699E-02
P 18= 1.72697E-02
P 19= 1.72695E-02

P 20= 1.72694E-02
P 21= 1.72693E-02
P 22= 1.72692E-02
P 23= 1.72692E-02
P 24= 1.72692E-02
P 25= 1.72692E-02
P 26= 1.72692E-02
P 27= 1.72691E-02
P 28= 1.72691E-02
P 29= 1.72691E-02
P 30= 1.72691E-02
P 31= 1.72691E-02
P 32= 1.72691E-02
P 33= 1.72691E-02

FOR RANDOM NUMBER N = 62 P1...PN IS

P 1= 5.00792E-02
P 2= 2.86009E-02
P 3= 1.67495E-02
P 4= 1.59139E-02
P 5= 1.58840E-02
P 6= 1.56547E-02
P 7= 1.54513E-02
P 8= 1.54255E-02
P 9= 1.53198E-02
P 10= 1.53175E-02
P 11= 1.53038E-02
P 12= 1.53023E-02
P 13= 1.53015E-02
P 14= 1.53014E-02
P 15= 1.53011E-02
P 16= 1.53009E-02
P 17= 1.53005E-02
P 18= 1.52998E-02
P 19= 1.52998E-02
P 20= 1.52998E-02
P 21= 1.52998E-02
P 22= 1.52998E-02
P 23= 1.52998E-02
P 24= 1.52998E-02
P 25= 1.52998E-02
P 26= 1.52998E-02
P 27= 1.52998E-02
P 28= 1.52998E-02
P 29= 1.52998E-02

P 30= 1.52998E-02
P 31= 1.52998E-02
P 32= 1.52998E-02
P 33= 1.52998E-02
P 34= 1.52998E-02
P 35= 1.52998E-02
P 36= 1.52998E-02
P 37= 1.52998E-02
P 38= 1.52998E-02
P 39= 1.52998E-02
P 40= 1.52998E-02
P 41= 1.52998E-02
P 42= 1.52998E-02
P 43= 1.52998E-02
P 44= 1.52998E-02
P 45= 1.52998E-02
P 46= 1.52998E-02
P 47= 1.52998E-02
P 48= 1.52998E-02
P 49= 1.52998E-02
P 50= 1.52998E-02
P 51= 1.52998E-02
P 52= 1.52998E-02
P 53= 1.52998E-02
P 54= 1.52998E-02
P 55= 1.52998E-02
P 56= 1.52998E-02
P 57= 1.52998E-02
P 58= 1.52998E-02
P 59= 1.52998E-02
P 60= 1.52998E-02
P 61= 1.52998E-02
P 62= 1.52998E-02

FOR RANDOM NUMBER N = 80 P1...PN IS

P 1= 0.393512
P 2= 4.90083E-02
P 3= 2.09531E-02
P 4= 9.58803E-03
P 5= 8.00581E-03
P 6= 7.31015E-03
P 7= 7.00061E-03
P 8= 6.96418E-03
P 9= 6.93646E-03
P 10= 6.93484E-03

P 11= 6.93074E-03
P 12= 6.92854E-03
P 13= 6.91829E-03
P 14= 6.91156E-03
P 15= 6.91104E-03
P 16= 6.91075E-03
P 17= 6.91071E-03
P 18= 6.91055E-03
P 19= 6.91055E-03
P 20= 6.91055E-03
P 21= 6.91055E-03
P 22= 6.91055E-03
P 23= 6.91055E-03
P 24= 6.91055E-03
P 25= 6.91055E-03
P 26= 6.91055E-03
P 27= 6.91055E-03
P 28= 6.91055E-03
P 29= 6.91055E-03
P 30= 6.91055E-03
P 31= 6.91055E-03
P 32= 6.91055E-03
P 33= 6.91055E-03
P 34= 6.91055E-03
P 35= 6.91054E-03
P 36= 6.91054E-03
P 37= 6.91054E-03
P 38= 6.91054E-03
P 39= 6.91054E-03
P 40= 6.91054E-03
P 41= 6.91054E-03
P 42= 6.91054E-03
P 43= 6.91054E-03
P 44= 6.91054E-03
P 45= 6.91054E-03
P 46= 6.91054E-03
P 47= 6.91054E-03
P 48= 6.91054E-03
P 49= 6.91054E-03
P 50= 6.91054E-03
P 51= 6.91053E-03
P 52= 6.91053E-03
P 53= 6.91053E-03
P 54= 6.91053E-03
P 55= 6.91053E-03
P 56= 6.91053E-03

P 57= 6.91053E-03
P 58= 6.91053E-03
P 59= 6.91053E-03
P 60= 6.91053E-03
P 61= 6.91053E-03
P 62= 6.91053E-03
P 63= 6.91053E-03
P 64= 6.91053E-03
P 65= 6.91053E-03
P 66= 6.91053E-03
P 67= 6.91053E-03
P 68= 6.91053E-03
P 69= 6.91053E-03
P 70= 6.91054E-03
P 71= 6.91053E-03
P 72= 6.91053E-03
P 73= 6.91053E-03
P 74= 6.91053E-03
P 75= 6.91053E-03
P 76= 6.91053E-03
P 77= 6.91054E-03
P 78= 6.91054E-03
P 79= 6.91054E-03
P 80= 6.91050E-03

FOR RANDOM NUMBER N = 20 P1...PN IS

P 1= 0.760250
P 2= 1.42182E-02
P 3= 1.37555E-02
P 4= 1.34781E-02
P 5= 1.29865E-02
P 6= 1.25951E-02
P 7= 1.25328E-02
P 8= 1.24363E-02
P 9= 1.24089E-02
P 10= 1.23477E-02
P 11= 1.23037E-02
P 12= 1.23029E-02
P 13= 1.23007E-02
P 14= 1.22983E-02
P 15= 1.22979E-02
P 16= 1.22978E-02
P 17= 1.22976E-02
P 18= 1.22973E-02
P 19= 1.22973E-02

P 20= 1.22972E-02

FOR RANDOM NUMBER N = 40 P1...PN IS

P 1= 2.56221E-02
P 2= 2.50379E-02
P 3= 2.50223E-02
P 4= 2.50125E-02
P 5= 2.49982E-02
P 6= 2.49820E-02
P 7= 2.49809E-02
P 8= 2.49805E-02
P 9= 2.49804E-02
P 10= 2.49801E-02
P 11= 2.49801E-02
P 12= 2.49801E-02
P 13= 2.49801E-02
P 14= 2.49801E-02
P 15= 2.49801E-02
P 16= 2.49801E-02
P 17= 2.49801E-02
P 18= 2.49801E-02
P 19= 2.49801E-02
P 20= 2.49801E-02
P 21= 2.49801E-02
P 22= 2.49801E-02
P 23= 2.49801E-02
P 24= 2.49801E-02
P 25= 2.49801E-02
P 26= 2.49801E-02
P 27= 2.49801E-02
P 28= 2.49801E-02
P 29= 2.49801E-02
P 30= 2.49801E-02
P 31= 2.49801E-02
P 32= 2.49801E-02
P 33= 2.49801E-02
P 34= 2.49801E-02
P 35= 2.49801E-02
P 36= 2.49801E-02
P 37= 2.49801E-02
P 38= 2.49801E-02
P 39= 2.49801E-02
P 40= 2.49801E-02

FOR RANDOM NUMBER N = 94 P1...PN IS

P 1= 9.83396E-02
P 2= 3.26218E-02
P 3= 1.04718E-02
P 4= 9.99180E-03
P 5= 9.69584E-03
P 6= 9.63539E-03
P 7= 9.60469E-03
P 8= 9.50359E-03
P 9= 9.45529E-03
P 10= 9.44470E-03
P 11= 9.43682E-03
P 12= 9.42282E-03
P 13= 9.42131E-03
P 14= 9.42038E-03
P 15= 9.41919E-03
P 16= 9.41918E-03
P 17= 9.41918E-03
P 18= 9.41918E-03
P 19= 9.41918E-03
P 20= 9.41918E-03
P 21= 9.41918E-03
P 22= 9.41918E-03
P 23= 9.41918E-03
P 24= 9.41918E-03
P 25= 9.41918E-03
P 26= 9.41918E-03
P 27= 9.41918E-03
P 28= 9.41918E-03
P 29= 9.41918E-03
P 30= 9.41918E-03
P 31= 9.41918E-03
P 32= 9.41918E-03
P 33= 9.41918E-03
P 34= 9.41918E-03
P 35= 9.41918E-03
P 36= 9.41918E-03
P 37= 9.41918E-03
P 38= 9.41918E-03
P 39= 9.41918E-03
P 40= 9.41918E-03
P 41= 9.41918E-03
P 42= 9.41918E-03
P 43= 9.41918E-03
P 44= 9.41918E-03
P 45= 9.41918E-03

P 46= 9.41918E-03
P 47= 9.41918E-03
P 48= 9.41918E-03
P 49= 9.41918E-03
P 50= 9.41918E-03
P 51= 9.41918E-03
P 52= 9.41918E-03
P 53= 9.41917E-03
P 54= 9.41917E-03
P 55= 9.41917E-03
P 56= 9.41917E-03
P 57= 9.41917E-03
P 58= 9.41917E-03
P 59= 9.41917E-03
P 60= 9.41917E-03
P 61= 9.41917E-03
P 62= 9.41917E-03
P 63= 9.41917E-03
P 64= 9.41917E-03
P 65= 9.41917E-03
P 66= 9.41917E-03
P 67= 9.41917E-03
P 68= 9.41917E-03
P 69= 9.41917E-03
P 70= 9.41917E-03
P 71= 9.41917E-03
P 72= 9.41917E-03
P 73= 9.41917E-03
P 74= 9.41917E-03
P 75= 9.41917E-03
P 76= 9.41917E-03
P 77= 9.41917E-03
P 78= 9.41917E-03
P 79= 9.41917E-03
P 80= 9.41917E-03
P 81= 9.41917E-03
P 82= 9.41917E-03
P 83= 9.41916E-03
P 84= 9.41916E-03
P 85= 9.41917E-03
P 86= 9.41917E-03
P 87= 9.41917E-03
P 88= 9.41918E-03
P 89= 9.41918E-03
P 90= 9.41918E-03
P 91= 9.41916E-03

P 92= 9.41916E-03
P 93= 9.41917E-03
P 94= 9.41920E-03

FOR RANDOM NUMBER N = 9 P1...PN IS

P 1= 0.960137
P 2= 9.16727E-03
P 3= 7.77822E-03
P 4= 7.29926E-03
P 5= 3.31676E-03
P 6= 3.25761E-03
P 7= 3.19097E-03
P 8= 3.01054E-03
P 9= 2.84261E-03

FOR RANDOM NUMBER N = 71 P1...PN IS

P 1= 2.92107E-02
P 2= 2.25127E-02
P 3= 1.73256E-02
P 4= 1.55619E-02
P 5= 1.45829E-02
P 6= 1.41640E-02
P 7= 1.36697E-02
P 8= 1.36695E-02
P 9= 1.36409E-02
P 10= 1.36408E-02
P 11= 1.36404E-02
P 12= 1.36401E-02
P 13= 1.36397E-02
P 14= 1.36397E-02
P 15= 1.36397E-02
P 16= 1.36397E-02
P 17= 1.36397E-02
P 18= 1.36397E-02
P 19= 1.36397E-02
P 20= 1.36397E-02
P 21= 1.36397E-02
P 22= 1.36397E-02
P 23= 1.36397E-02
P 24= 1.36397E-02
P 25= 1.36397E-02
P 26= 1.36397E-02
P 27= 1.36397E-02
P 28= 1.36397E-02

P 29= 1.36397E-02
P 30= 1.36397E-02
P 31= 1.36397E-02
P 32= 1.36397E-02
P 33= 1.36397E-02
P 34= 1.36397E-02
P 35= 1.36397E-02
P 36= 1.36397E-02
P 37= 1.36397E-02
P 38= 1.36397E-02
P 39= 1.36397E-02
P 40= 1.36397E-02
P 41= 1.36397E-02
P 42= 1.36397E-02
P 43= 1.36397E-02
P 44= 1.36397E-02
P 45= 1.36397E-02
P 46= 1.36397E-02
P 47= 1.36397E-02
P 48= 1.36397E-02
P 49= 1.36397E-02
P 50= 1.36397E-02
P 51= 1.36397E-02
P 52= 1.36397E-02
P 53= 1.36397E-02
P 54= 1.36397E-02
P 55= 1.36397E-02
P 56= 1.36397E-02
P 57= 1.36397E-02
P 58= 1.36397E-02
P 59= 1.36397E-02
P 60= 1.36397E-02
P 61= 1.36397E-02
P 62= 1.36397E-02
P 63= 1.36397E-02
P 64= 1.36397E-02
P 65= 1.36397E-02
P 66= 1.36397E-02
P 67= 1.36397E-02
P 68= 1.36397E-02
P 69= 1.36397E-02
P 70= 1.36397E-02
P 71= 1.36397E-02

FOR RANDOM NUMBER N = 21 P1...PN IS

P 1= 0.127957
P 2= 8.12589E-02
P 3= 4.21735E-02
P 4= 4.17437E-02
P 5= 4.16610E-02
P 6= 4.16010E-02
P 7= 4.15768E-02
P 8= 4.15750E-02
P 9= 4.15738E-02
P 10= 4.15738E-02
P 11= 4.15735E-02
P 12= 4.15734E-02
P 13= 4.15733E-02
P 14= 4.15733E-02
P 15= 4.15733E-02
P 16= 4.15733E-02
P 17= 4.15733E-02
P 18= 4.15732E-02
P 19= 4.15732E-02
P 20= 4.15732E-02
P 21= 4.15732E-02

FOR RANDOM NUMBER N = 34 P1...PN IS

P 1= 0.406669
P 2= 2.12909E-02
P 3= 1.99472E-02
P 4= 1.78253E-02
P 5= 1.78241E-02
P 6= 1.78121E-02
P 7= 1.78102E-02
P 8= 1.78092E-02
P 9= 1.78087E-02
P 10= 1.78085E-02
P 11= 1.78082E-02
P 12= 1.78081E-02
P 13= 1.78081E-02
P 14= 1.78081E-02
P 15= 1.78081E-02
P 16= 1.78081E-02
P 17= 1.78081E-02
P 18= 1.78081E-02
P 19= 1.78081E-02
P 20= 1.78081E-02
P 21= 1.78081E-02
P 22= 1.78081E-02

P 23= 1.78081E-02
P 24= 1.78081E-02
P 25= 1.78081E-02
P 26= 1.78081E-02
P 27= 1.78081E-02
P 28= 1.78081E-02
P 29= 1.78081E-02
P 30= 1.78081E-02
P 31= 1.78081E-02
P 32= 1.78081E-02
P 33= 1.78081E-02
P 34= 1.78081E-02

FOR RANDOM NUMBER N = 48 P1...PN IS

P 1= 0.228096
P 2= 1.79200E-02
P 3= 1.68114E-02
P 4= 1.65013E-02
P 5= 1.63932E-02
P 6= 1.63852E-02
P 7= 1.63829E-02
P 8= 1.63810E-02
P 9= 1.63804E-02
P 10= 1.63782E-02
P 11= 1.63782E-02
P 12= 1.63782E-02
P 13= 1.63782E-02
P 14= 1.63782E-02
P 15= 1.63782E-02
P 16= 1.63782E-02
P 17= 1.63782E-02
P 18= 1.63782E-02
P 19= 1.63782E-02
P 20= 1.63782E-02
P 21= 1.63782E-02
P 22= 1.63782E-02
P 23= 1.63782E-02
P 24= 1.63782E-02
P 25= 1.63782E-02
P 26= 1.63782E-02
P 27= 1.63782E-02
P 28= 1.63782E-02
P 29= 1.63782E-02
P 30= 1.63782E-02
P 31= 1.63782E-02

P 32= 1.63782E-02
P 33= 1.63782E-02
P 34= 1.63782E-02
P 35= 1.63782E-02
P 36= 1.63782E-02
P 37= 1.63782E-02
P 38= 1.63782E-02
P 39= 1.63782E-02
P 40= 1.63782E-02
P 41= 1.63781E-02
P 42= 1.63781E-02
P 43= 1.63781E-02
P 44= 1.63781E-02
P 45= 1.63781E-02
P 46= 1.63781E-02
P 47= 1.63781E-02
P 48= 1.63781E-02

FOR RANDOM NUMBER N = 83 P1...PN IS

P 1= 3.35148E-02
P 2= 2.60385E-02
P 3= 1.91458E-02
P 4= 1.43380E-02
P 5= 1.21209E-02
P 6= 1.18904E-02
P 7= 1.17417E-02
P 8= 1.15750E-02
P 9= 1.15454E-02
P 10= 1.14964E-02
P 11= 1.14899E-02
P 12= 1.14826E-02
P 13= 1.14675E-02
P 14= 1.14623E-02
P 15= 1.14619E-02
P 16= 1.14597E-02
P 17= 1.14597E-02
P 18= 1.14594E-02
P 19= 1.14593E-02
P 20= 1.14593E-02
P 21= 1.14593E-02
P 22= 1.14593E-02
P 23= 1.14592E-02
P 24= 1.14592E-02
P 25= 1.14592E-02
P 26= 1.14592E-02

P 27= 1.14592E-02
P 28= 1.14592E-02
P 29= 1.14592E-02
P 30= 1.14592E-02
P 31= 1.14592E-02
P 32= 1.14592E-02
P 33= 1.14592E-02
P 34= 1.14592E-02
P 35= 1.14592E-02
P 36= 1.14592E-02
P 37= 1.14592E-02
P 38= 1.14592E-02
P 39= 1.14592E-02
P 40= 1.14592E-02
P 41= 1.14592E-02
P 42= 1.14592E-02
P 43= 1.14592E-02
P 44= 1.14592E-02
P 45= 1.14592E-02
P 46= 1.14592E-02
P 47= 1.14592E-02
P 48= 1.14592E-02
P 49= 1.14592E-02
P 50= 1.14592E-02
P 51= 1.14592E-02
P 52= 1.14592E-02
P 53= 1.14592E-02
P 54= 1.14592E-02
P 55= 1.14592E-02
P 56= 1.14592E-02
P 57= 1.14592E-02
P 58= 1.14592E-02
P 59= 1.14592E-02
P 60= 1.14592E-02
P 61= 1.14592E-02
P 62= 1.14592E-02
P 63= 1.14592E-02
P 64= 1.14592E-02
P 65= 1.14592E-02
P 66= 1.14592E-02
P 67= 1.14592E-02
P 68= 1.14592E-02
P 69= 1.14592E-02
P 70= 1.14592E-02
P 71= 1.14592E-02
P 72= 1.14592E-02

P 73= 1.14592E-02
P 74= 1.14592E-02
P 75= 1.14592E-02
P 76= 1.14592E-02
P 77= 1.14592E-02
P 78= 1.14592E-02
P 79= 1.14592E-02
P 80= 1.14592E-02
P 81= 1.14592E-02
P 82= 1.14592E-02
P 83= 1.14592E-02

FOR RANDOM NUMBER N = 57 P1...PN IS

P 1= 2.76192E-02
P 2= 2.54463E-02
P 3= 2.18782E-02
P 4= 1.90584E-02
P 5= 1.84181E-02
P 6= 1.83171E-02
P 7= 1.74803E-02
P 8= 1.74327E-02
P 9= 1.71380E-02
P 10= 1.70270E-02
P 11= 1.70256E-02
P 12= 1.70255E-02
P 13= 1.70254E-02
P 14= 1.70252E-02
P 15= 1.70252E-02
P 16= 1.70252E-02
P 17= 1.70252E-02
P 18= 1.70252E-02
P 19= 1.70252E-02
P 20= 1.70252E-02
P 21= 1.70252E-02
P 22= 1.70252E-02
P 23= 1.70252E-02
P 24= 1.70252E-02
P 25= 1.70252E-02
P 26= 1.70252E-02
P 27= 1.70252E-02
P 28= 1.70252E-02
P 29= 1.70252E-02
P 30= 1.70252E-02
P 31= 1.70252E-02
P 32= 1.70252E-02

P 33= 1.70252E-02
P 34= 1.70252E-02
P 35= 1.70252E-02
P 36= 1.70252E-02
P 37= 1.70252E-02
P 38= 1.70252E-02
P 39= 1.70252E-02
P 40= 1.70252E-02
P 41= 1.70252E-02
P 42= 1.70252E-02
P 43= 1.70252E-02
P 44= 1.70252E-02
P 45= 1.70252E-02
P 46= 1.70252E-02
P 47= 1.70252E-02
P 48= 1.70252E-02
P 49= 1.70252E-02
P 50= 1.70252E-02
P 51= 1.70252E-02
P 52= 1.70252E-02
P 53= 1.70252E-02
P 54= 1.70252E-02
P 55= 1.70252E-02
P 56= 1.70252E-02
P 57= 1.70252E-02

FOR RANDOM NUMBER N = 68 P1...PN IS

P 1= 6.37567E-02
P 2= 2.16906E-02
P 3= 1.49502E-02
P 4= 1.43832E-02
P 5= 1.41470E-02
P 6= 1.40230E-02
P 7= 1.39039E-02
P 8= 1.38995E-02
P 9= 1.38302E-02
P 10= 1.38245E-02
P 11= 1.38213E-02
P 12= 1.38209E-02
P 13= 1.38206E-02
P 14= 1.38205E-02
P 15= 1.38205E-02
P 16= 1.38205E-02
P 17= 1.38205E-02
P 18= 1.38205E-02

P 19= 1.38205E-02
P 20= 1.38205E-02
P 21= 1.38205E-02
P 22= 1.38205E-02
P 23= 1.38205E-02
P 24= 1.38205E-02
P 25= 1.38205E-02
P 26= 1.38205E-02
P 27= 1.38205E-02
P 28= 1.38205E-02
P 29= 1.38205E-02
P 30= 1.38205E-02
P 31= 1.38205E-02
P 32= 1.38205E-02
P 33= 1.38205E-02
P 34= 1.38205E-02
P 35= 1.38205E-02
P 36= 1.38205E-02
P 37= 1.38205E-02
P 38= 1.38205E-02
P 39= 1.38205E-02
P 40= 1.38205E-02
P 41= 1.38205E-02
P 42= 1.38205E-02
P 43= 1.38205E-02
P 44= 1.38205E-02
P 45= 1.38205E-02
P 46= 1.38205E-02
P 47= 1.38205E-02
P 48= 1.38205E-02
P 49= 1.38205E-02
P 50= 1.38205E-02
P 51= 1.38205E-02
P 52= 1.38205E-02
P 53= 1.38205E-02
P 54= 1.38205E-02
P 55= 1.38205E-02
P 56= 1.38205E-02
P 57= 1.38205E-02
P 58= 1.38205E-02
P 59= 1.38205E-02
P 60= 1.38205E-02
P 61= 1.38205E-02
P 62= 1.38205E-02
P 63= 1.38205E-02
P 64= 1.38205E-02

P 65= 1.38205E-02
P 66= 1.38205E-02
P 67= 1.38205E-02
P 68= 1.38205E-02

FOR RANDOM NUMBER N = 52 P1...PN IS

P 1= 0.251368
P 2= 0.198256
P 3= 6.17100E-02
P 4= 3.10997E-02
P 5= 2.48125E-02
P 6= 1.09159E-02
P 7= 1.07818E-02
P 8= 9.16552E-03
P 9= 9.16499E-03
P 10= 9.16391E-03
P 11= 9.14460E-03
P 12= 9.13810E-03
P 13= 9.13717E-03
P 14= 9.13377E-03
P 15= 9.13328E-03
P 16= 9.13216E-03
P 17= 9.13209E-03
P 18= 9.13201E-03
P 19= 9.13195E-03
P 20= 9.13193E-03
P 21= 9.13181E-03
P 22= 9.13175E-03
P 23= 9.13172E-03
P 24= 9.13171E-03
P 25= 9.13171E-03
P 26= 9.13171E-03
P 27= 9.13171E-03
P 28= 9.13170E-03
P 29= 9.13170E-03
P 30= 9.13170E-03
P 31= 9.13170E-03
P 32= 9.13170E-03
P 33= 9.13170E-03
P 34= 9.13170E-03
P 35= 9.13170E-03
P 36= 9.13170E-03
P 37= 9.13170E-03
P 38= 9.13170E-03
P 39= 9.13170E-03

P 40= 9.13170E-03
P 41= 9.13170E-03
P 42= 9.13170E-03
P 43= 9.13170E-03
P 44= 9.13170E-03
P 45= 9.13170E-03
P 46= 9.13170E-03
P 47= 9.13171E-03
P 48= 9.13171E-03
P 49= 9.13170E-03
P 50= 9.13169E-03
P 51= 9.13170E-03
P 52= 9.13172E-03

FOR RANDOM NUMBER N = 43 P1...PN IS

P 1= 0.968097
P 2= 2.93494E-03
P 3= 2.75316E-03
P 4= 2.63818E-03
P 5= 1.00622E-03
P 6= 7.05707E-04
P 7= 6.22689E-04
P 8= 5.97185E-04
P 9= 5.94060E-04
P 10= 5.90150E-04
P 11= 5.89742E-04
P 12= 5.89715E-04
P 13= 5.89710E-04
P 14= 5.89709E-04
P 15= 5.89708E-04
P 16= 5.89708E-04
P 17= 5.89706E-04
P 18= 5.89706E-04
P 19= 5.89705E-04
P 20= 5.89705E-04
P 21= 5.89703E-04
P 22= 5.89702E-04
P 23= 5.89700E-04
P 24= 5.89699E-04
P 25= 5.89697E-04
P 26= 5.89698E-04
P 27= 5.89699E-04
P 28= 5.89699E-04
P 29= 5.89699E-04
P 30= 5.89697E-04

P 31= 5.89697E-04
P 32= 5.89698E-04
P 33= 5.89699E-04
P 34= 5.89699E-04
P 35= 5.89698E-04
P 36= 5.89698E-04
P 37= 5.89700E-04
P 38= 5.89699E-04
P 39= 5.89693E-04
P 40= 5.89693E-04
P 41= 5.89707E-04
P 42= 5.89701E-04
P 43= 5.89669E-04

FOR RANDOM NUMBER N = 52 P1...PN IS

P 1= 0.483565
P 2= 0.161153
P 3= 5.31182E-02
P 4= 7.76621E-03
P 5= 6.79295E-03
P 6= 6.66727E-03
P 7= 6.23836E-03
P 8= 6.19342E-03
P 9= 6.18322E-03
P 10= 6.16524E-03
P 11= 6.12326E-03
P 12= 6.10452E-03
P 13= 6.10259E-03
P 14= 6.10172E-03
P 15= 6.10070E-03
P 16= 6.09825E-03
P 17= 6.09820E-03
P 18= 6.09806E-03
P 19= 6.09801E-03
P 20= 6.09797E-03
P 21= 6.09796E-03
P 22= 6.09796E-03
P 23= 6.09796E-03
P 24= 6.09796E-03
P 25= 6.09796E-03
P 26= 6.09795E-03
P 27= 6.09795E-03
P 28= 6.09795E-03
P 29= 6.09795E-03
P 30= 6.09795E-03

P 31= 6.09795E-03
P 32= 6.09795E-03
P 33= 6.09795E-03
P 34= 6.09794E-03
P 35= 6.09794E-03
P 36= 6.09794E-03
P 37= 6.09794E-03
P 38= 6.09794E-03
P 39= 6.09794E-03
P 40= 6.09794E-03
P 41= 6.09794E-03
P 42= 6.09794E-03
P 43= 6.09794E-03
P 44= 6.09794E-03
P 45= 6.09794E-03
P 46= 6.09794E-03
P 47= 6.09794E-03
P 48= 6.09794E-03
P 49= 6.09794E-03
P 50= 6.09795E-03
P 51= 6.09795E-03
P 52= 6.09791E-03

FOR RANDOM NUMBER N = 56 P1...PN IS

P 1= 2.15459E-02
P 2= 1.83190E-02
P 3= 1.81556E-02
P 4= 1.81016E-02
P 5= 1.79611E-02
P 6= 1.79474E-02
P 7= 1.78803E-02
P 8= 1.78695E-02
P 9= 1.77568E-02
P 10= 1.77551E-02
P 11= 1.77546E-02
P 12= 1.77546E-02
P 13= 1.77546E-02
P 14= 1.77545E-02
P 15= 1.77545E-02
P 16= 1.77545E-02
P 17= 1.77545E-02
P 18= 1.77545E-02
P 19= 1.77545E-02
P 20= 1.77545E-02
P 21= 1.77545E-02

P 22=   1.77545E-02
P 23=   1.77545E-02
P 24=   1.77545E-02
P 25=   1.77545E-02
P 26=   1.77545E-02
P 27=   1.77545E-02
P 28=   1.77545E-02
P 29=   1.77545E-02
P 30=   1.77545E-02
P 31=   1.77545E-02
P 32=   1.77545E-02
P 33=   1.77545E-02
P 34=   1.77545E-02
P 35=   1.77545E-02
P 36=   1.77545E-02
P 37=   1.77545E-02
P 38=   1.77545E-02
P 39=   1.77545E-02
P 40=   1.77545E-02
P 41=   1.77545E-02
P 42=   1.77545E-02
P 43=   1.77545E-02
P 44=   1.77545E-02
P 45=   1.77545E-02
P 46=   1.77545E-02
P 47=   1.77545E-02
P 48=   1.77545E-02
P 49=   1.77545E-02
P 50=   1.77545E-02
P 51=   1.77545E-02
P 52=   1.77545E-02
P 53=   1.77545E-02
P 54=   1.77545E-02
P 55=   1.77545E-02
P 56=   1.77545E-02

FOR RANDOM NUMBER N = 99   P1...PN IS

P 1=   1.42794E-02
P 2=   1.03108E-02
P 3=   1.01879E-02
P 4=   1.00993E-02
P 5=   1.00702E-02
P 6=   1.00684E-02
P 7=   1.00543E-02
P 8=   1.00541E-02

P 9= 1.00538E-02
P 10= 1.00536E-02
P 11= 1.00536E-02
P 12= 1.00536E-02
P 13= 1.00536E-02
P 14= 1.00536E-02
P 15= 1.00536E-02
P 16= 1.00536E-02
P 17= 1.00536E-02
P 18= 1.00536E-02
P 19= 1.00536E-02
P 20= 1.00536E-02
P 21= 1.00536E-02
P 22= 1.00536E-02
P 23= 1.00536E-02
P 24= 1.00536E-02
P 25= 1.00536E-02
P 26= 1.00536E-02
P 27= 1.00536E-02
P 28= 1.00536E-02
P 29= 1.00536E-02
P 30= 1.00536E-02
P 31= 1.00536E-02
P 32= 1.00536E-02
P 33= 1.00536E-02
P 34= 1.00536E-02
P 35= 1.00536E-02
P 36= 1.00536E-02
P 37= 1.00536E-02
P 38= 1.00536E-02
P 39= 1.00536E-02
P 40= 1.00536E-02
P 41= 1.00536E-02
P 42= 1.00536E-02
P 43= 1.00536E-02
P 44= 1.00536E-02
P 45= 1.00536E-02
P 46= 1.00536E-02
P 47= 1.00536E-02
P 48= 1.00536E-02
P 49= 1.00536E-02
P 50= 1.00536E-02
P 51= 1.00536E-02
P 52= 1.00536E-02
P 53= 1.00536E-02
P 54= 1.00536E-02

P 55= 1.00536E-02
P 56= 1.00536E-02
P 57= 1.00536E-02
P 58= 1.00536E-02
P 59= 1.00536E-02
P 60= 1.00536E-02
P 61= 1.00536E-02
P 62= 1.00536E-02
P 63= 1.00536E-02
P 64= 1.00536E-02
P 65= 1.00536E-02
P 66= 1.00536E-02
P 67= 1.00536E-02
P 68= 1.00536E-02
P 69= 1.00536E-02
P 70= 1.00536E-02
P 71= 1.00536E-02
P 72= 1.00536E-02
P 73= 1.00536E-02
P 74= 1.00536E-02
P 75= 1.00536E-02
P 76= 1.00536E-02
P 77= 1.00536E-02
P 78= 1.00536E-02
P 79= 1.00536E-02
P 80= 1.00536E-02
P 81= 1.00536E-02
P 82= 1.00536E-02
P 83= 1.00536E-02
P 84= 1.00536E-02
P 85= 1.00536E-02
P 86= 1.00536E-02
P 87= 1.00536E-02
P 88= 1.00536E-02
P 89= 1.00536E-02
P 90= 1.00536E-02
P 91= 1.00536E-02
P 92= 1.00536E-02
P 93= 1.00536E-02
P 94= 1.00536E-02
P 95= 1.00536E-02
P 96= 1.00536E-02
P 97= 1.00536E-02
P 98= 1.00536E-02
P 99= 1.00536E-02

FOR RANDOM NUMBER N = 35   P1...PN IS

P 1=   0.754882
P 2=   9.99017E-03
P 3=   9.88158E-03
P 4=   7.53560E-03
P 5=   7.30320E-03
P 6=   7.28307E-03
P 7=   7.27469E-03
P 8=   7.07687E-03
P 9=   7.02237E-03
P 10=   6.99867E-03
P 11=   6.99614E-03
P 12=   6.99339E-03
P 13=   6.99141E-03
P 14=   6.99035E-03
P 15=   6.98990E-03
P 16=   6.98986E-03
P 17=   6.98985E-03
P 18=   6.98974E-03
P 19=   6.98973E-03
P 20=   6.98972E-03
P 21=   6.98959E-03
P 22=   6.98948E-03
P 23=   6.98946E-03
P 24=   6.98946E-03
P 25=   6.98946E-03
P 26=   6.98946E-03
P 27=   6.98945E-03
P 28=   6.98943E-03
P 29=   6.98944E-03
P 30=   6.98944E-03
P 31=   6.98944E-03
P 32=   6.98945E-03
P 33=   6.98945E-03
P 34=   6.98945E-03
P 35=   6.98942E-03

FOR RANDOM NUMBER N =   4   P1...PN IS

P 1=   0.395342
P 2=   0.212762
P 3=   0.205795
P 4=   0.186101

FOR RANDOM NUMBER N = 69   P1...PN IS

P 1= 2.22715E-02
P 2= 1.80402E-02
P 3= 1.61528E-02
P 4= 1.52986E-02
P 5= 1.52889E-02
P 6= 1.49321E-02
P 7= 1.48468E-02
P 8= 1.46257E-02
P 9= 1.44483E-02
P 10= 1.43647E-02
P 11= 1.42946E-02
P 12= 1.42740E-02
P 13= 1.42373E-02
P 14= 1.42339E-02
P 15= 1.42333E-02
P 16= 1.42316E-02
P 17= 1.42308E-02
P 18= 1.42307E-02
P 19= 1.42307E-02
P 20= 1.42307E-02
P 21= 1.42307E-02
P 22= 1.42307E-02
P 23= 1.42307E-02
P 24= 1.42307E-02
P 25= 1.42307E-02
P 26= 1.42307E-02
P 27= 1.42307E-02
P 28= 1.42307E-02
P 29= 1.42307E-02
P 30= 1.42307E-02
P 31= 1.42307E-02
P 32= 1.42307E-02
P 33= 1.42307E-02
P 34= 1.42307E-02
P 35= 1.42307E-02
P 36= 1.42307E-02
P 37= 1.42307E-02
P 38= 1.42307E-02
P 39= 1.42307E-02
P 40= 1.42307E-02
P 41= 1.42307E-02
P 42= 1.42307E-02
P 43= 1.42307E-02
P 44= 1.42307E-02
P 45= 1.42307E-02

P 46= 1.42307E-02
P 47= 1.42307E-02
P 48= 1.42307E-02
P 49= 1.42307E-02
P 50= 1.42307E-02
P 51= 1.42307E-02
P 52= 1.42307E-02
P 53= 1.42307E-02
P 54= 1.42307E-02
P 55= 1.42307E-02
P 56= 1.42307E-02
P 57= 1.42307E-02
P 58= 1.42307E-02
P 59= 1.42307E-02
P 60= 1.42307E-02
P 61= 1.42307E-02
P 62= 1.42307E-02
P 63= 1.42307E-02
P 64= 1.42307E-02
P 65= 1.42307E-02
P 66= 1.42307E-02
P 67= 1.42307E-02
P 68= 1.42307E-02
P 69= 1.42307E-02

FOR RANDOM NUMBER N = 95 P1...PN IS

P 1= 8.19830E-02
P 2= 3.10859E-02
P 3= 1.04606E-02
P 4= 9.97475E-03
P 5= 9.54216E-03
P 6= 9.53556E-03
P 7= 9.53040E-03
P 8= 9.52647E-03
P 9= 9.52633E-03
P 10= 9.52405E-03
P 11= 9.52131E-03
P 12= 9.52131E-03
P 13= 9.52131E-03
P 14= 9.52130E-03
P 15= 9.52130E-03
P 16= 9.52130E-03
P 17= 9.52130E-03
P 18= 9.52130E-03
P 19= 9.52130E-03

P 20= 9.52130E-03
P 21= 9.52130E-03
P 22= 9.52130E-03
P 23= 9.52130E-03
P 24= 9.52130E-03
P 25= 9.52130E-03
P 26= 9.52130E-03
P 27= 9.52130E-03
P 28= 9.52130E-03
P 29= 9.52130E-03
P 30= 9.52130E-03
P 31= 9.52130E-03
P 32= 9.52130E-03
P 33= 9.52130E-03
P 34= 9.52130E-03
P 35= 9.52130E-03
P 36= 9.52130E-03
P 37= 9.52130E-03
P 38= 9.52130E-03
P 39= 9.52130E-03
P 40= 9.52130E-03
P 41= 9.52130E-03
P 42= 9.52130E-03
P 43= 9.52130E-03
P 44= 9.52130E-03
P 45= 9.52130E-03
P 46= 9.52130E-03
P 47= 9.52130E-03
P 48= 9.52130E-03
P 49= 9.52130E-03
P 50= 9.52130E-03
P 51= 9.52130E-03
P 52= 9.52130E-03
P 53= 9.52130E-03
P 54= 9.52130E-03
P 55= 9.52130E-03
P 56= 9.52130E-03
P 57= 9.52130E-03
P 58= 9.52130E-03
P 59= 9.52130E-03
P 60= 9.52130E-03
P 61= 9.52130E-03
P 62= 9.52130E-03
P 63= 9.52130E-03
P 64= 9.52130E-03
P 65= 9.52130E-03

P 66= 9.52130E-03
P 67= 9.52130E-03
P 68= 9.52130E-03
P 69= 9.52130E-03
P 70= 9.52130E-03
P 71= 9.52130E-03
P 72= 9.52130E-03
P 73= 9.52130E-03
P 74= 9.52130E-03
P 75= 9.52130E-03
P 76= 9.52130E-03
P 77= 9.52130E-03
P 78= 9.52130E-03
P 79= 9.52130E-03
P 80= 9.52130E-03
P 81= 9.52130E-03
P 82= 9.52130E-03
P 83= 9.52129E-03
P 84= 9.52129E-03
P 85= 9.52129E-03
P 86= 9.52129E-03
P 87= 9.52129E-03
P 88= 9.52129E-03
P 89= 9.52128E-03
P 90= 9.52128E-03
P 91= 9.52127E-03
P 92= 9.52127E-03
P 93= 9.52128E-03
P 94= 9.52128E-03
P 95= 9.52125E-03

FOR RANDOM NUMBER N = 64 P1...PN IS

P 1= 7.24067E-02
P 2= 1.79347E-02
P 3= 1.53426E-02
P 4= 1.50768E-02
P 5= 1.50007E-02
P 6= 1.47455E-02
P 7= 1.46869E-02
P 8= 1.46736E-02
P 9= 1.46495E-02
P 10= 1.46481E-02
P 11= 1.46451E-02
P 12= 1.46451E-02
P 13= 1.46451E-02

P 14= 1.46451E-02
P 15= 1.46451E-02
P 16= 1.46451E-02
P 17= 1.46451E-02
P 18= 1.46451E-02
P 19= 1.46451E-02
P 20= 1.46451E-02
P 21= 1.46451E-02
P 22= 1.46451E-02
P 23= 1.46451E-02
P 24= 1.46451E-02
P 25= 1.46451E-02
P 26= 1.46451E-02
P 27= 1.46451E-02
P 28= 1.46451E-02
P 29= 1.46451E-02
P 30= 1.46451E-02
P 31= 1.46451E-02
P 32= 1.46451E-02
P 33= 1.46451E-02
P 34= 1.46451E-02
P 35= 1.46451E-02
P 36= 1.46451E-02
P 37= 1.46451E-02
P 38= 1.46451E-02
P 39= 1.46451E-02
P 40= 1.46451E-02
P 41= 1.46451E-02
P 42= 1.46451E-02
P 43= 1.46451E-02
P 44= 1.46451E-02
P 45= 1.46451E-02
P 46= 1.46451E-02
P 47= 1.46451E-02
P 48= 1.46451E-02
P 49= 1.46451E-02
P 50= 1.46451E-02
P 51= 1.46451E-02
P 52= 1.46451E-02
P 53= 1.46451E-02
P 54= 1.46451E-02
P 55= 1.46451E-02
P 56= 1.46451E-02
P 57= 1.46451E-02
P 58= 1.46451E-02
P 59= 1.46451E-02

P 60= 1.46451E-02
P 61= 1.46451E-02
P 62= 1.46451E-02
P 63= 1.46450E-02
P 64= 1.46450E-02

FOR RANDOM NUMBER N = 17 P1...PN IS

P 1= 0.651967
P 2= 5.39667E-02
P 3= 3.15069E-02
P 4= 2.30375E-02
P 5= 2.00378E-02
P 6= 1.92185E-02
P 7= 1.82786E-02
P 8= 1.82176E-02
P 9= 1.82048E-02
P 10= 1.82009E-02
P 11= 1.82009E-02
P 12= 1.81985E-02
P 13= 1.81948E-02
P 14= 1.81924E-02
P 15= 1.81924E-02
P 16= 1.81923E-02
P 17= 1.81922E-02

FOR RANDOM NUMBER N = 29 P1...PN IS

P 1= 0.841363
P 2= 7.27954E-02
P 3= 2.02885E-02
P 4= 9.80490E-03
P 5= 6.03002E-03
P 6= 3.71287E-03
P 7= 3.56351E-03
P 8= 1.97565E-03
P 9= 1.93933E-03
P 10= 1.93627E-03
P 11= 1.93437E-03
P 12= 1.93381E-03
P 13= 1.93043E-03
P 14= 1.92590E-03
P 15= 1.92469E-03
P 16= 1.92456E-03
P 17= 1.92446E-03
P 18= 1.92442E-03

P 19= 1.92442E-03
P 20= 1.92441E-03
P 21= 1.92440E-03
P 22= 1.92440E-03
P 23= 1.92440E-03
P 24= 1.92440E-03
P 25= 1.92440E-03
P 26= 1.92440E-03
P 27= 1.92440E-03
P 28= 1.92440E-03
P 29= 1.92440E-03

FOR RANDOM NUMBER N = 72 P1...PN IS

P 1= 2.78702E-02
P 2= 1.98456E-02
P 3= 1.89697E-02
P 4= 1.50287E-02
P 5= 1.47437E-02
P 6= 1.46654E-02
P 7= 1.44577E-02
P 8= 1.42637E-02
P 9= 1.42100E-02
P 10= 1.37355E-02
P 11= 1.36755E-02
P 12= 1.35274E-02
P 13= 1.34903E-02
P 14= 1.34847E-02
P 15= 1.34218E-02
P 16= 1.34210E-02
P 17= 1.34192E-02
P 18= 1.34153E-02
P 19= 1.34145E-02
P 20= 1.34140E-02
P 21= 1.34140E-02
P 22= 1.34140E-02
P 23= 1.34140E-02
P 24= 1.34140E-02
P 25= 1.34140E-02
P 26= 1.34140E-02
P 27= 1.34140E-02
P 28= 1.34140E-02
P 29= 1.34140E-02
P 30= 1.34140E-02
P 31= 1.34140E-02
P 32= 1.34140E-02

P 33= 1.34140E-02
P 34= 1.34140E-02
P 35= 1.34140E-02
P 36= 1.34140E-02
P 37= 1.34140E-02
P 38= 1.34140E-02
P 39= 1.34140E-02
P 40= 1.34140E-02
P 41= 1.34140E-02
P 42= 1.34140E-02
P 43= 1.34140E-02
P 44= 1.34140E-02
P 45= 1.34140E-02
P 46= 1.34140E-02
P 47= 1.34140E-02
P 48= 1.34140E-02
P 49= 1.34140E-02
P 50= 1.34140E-02
P 51= 1.34139E-02
P 52= 1.34139E-02
P 53= 1.34139E-02
P 54= 1.34139E-02
P 55= 1.34139E-02
P 56= 1.34139E-02
P 57= 1.34139E-02
P 58= 1.34139E-02
P 59= 1.34139E-02
P 60= 1.34139E-02
P 61= 1.34139E-02
P 62= 1.34139E-02
P 63= 1.34139E-02
P 64= 1.34139E-02
P 65= 1.34139E-02
P 66= 1.34139E-02
P 67= 1.34139E-02
P 68= 1.34139E-02
P 69= 1.34139E-02
P 70= 1.34139E-02
P 71= 1.34139E-02
P 72= 1.34139E-02

FOR RANDOM NUMBER N = 74 P1...PN IS

P 1= 0.503974
P 2= 0.151313
P 3= 9.57200E-03

P 4= 6.97151E-03
P 5= 5.31867E-03
P 6= 4.82480E-03
P 7= 4.82439E-03
P 8= 4.68184E-03
P 9= 4.67879E-03
P 10= 4.67660E-03
P 11= 4.67478E-03
P 12= 4.67462E-03
P 13= 4.67454E-03
P 14= 4.67453E-03
P 15= 4.67450E-03
P 16= 4.67449E-03
P 17= 4.67447E-03
P 18= 4.67446E-03
P 19= 4.67445E-03
P 20= 4.67444E-03
P 21= 4.67444E-03
P 22= 4.67444E-03
P 23= 4.67444E-03
P 24= 4.67445E-03
P 25= 4.67445E-03
P 26= 4.67445E-03
P 27= 4.67445E-03
P 28= 4.67445E-03
P 29= 4.67445E-03
P 30= 4.67445E-03
P 31= 4.67445E-03
P 32= 4.67445E-03
P 33= 4.67445E-03
P 34= 4.67445E-03
P 35= 4.67445E-03
P 36= 4.67445E-03
P 37= 4.67445E-03
P 38= 4.67445E-03
P 39= 4.67445E-03
P 40= 4.67445E-03
P 41= 4.67445E-03
P 42= 4.67445E-03
P 43= 4.67445E-03
P 44= 4.67445E-03
P 45= 4.67445E-03
P 46= 4.67445E-03
P 47= 4.67445E-03
P 48= 4.67445E-03
P 49= 4.67445E-03

P 50= 4.67446E-03
P 51= 4.67446E-03
P 52= 4.67446E-03
P 53= 4.67446E-03
P 54= 4.67446E-03
P 55= 4.67446E-03
P 56= 4.67446E-03
P 57= 4.67446E-03
P 58= 4.67446E-03
P 59= 4.67447E-03
P 60= 4.67447E-03
P 61= 4.67446E-03
P 62= 4.67446E-03
P 63= 4.67446E-03
P 64= 4.67446E-03
P 65= 4.67446E-03
P 66= 4.67446E-03
P 67= 4.67446E-03
P 68= 4.67447E-03
P 69= 4.67447E-03
P 70= 4.67446E-03
P 71= 4.67446E-03
P 72= 4.67447E-03
P 73= 4.67447E-03
P 74= 4.67443E-03

FOR RANDOM NUMBER N = 3 P1...PN IS

P 1= 0.822964
P 2= 0.158176
P 3= 1.88599E-02

FOR RANDOM NUMBER N = 34 P1...PN IS

P 1= 1.01783E-01
P 2= 6.61321E-02
P 3= 4.81189E-02
P 4= 3.03268E-02
P 5= 2.75949E-02
P 6= 2.72468E-02
P 7= 2.65077E-02
P 8= 2.51239E-02
P 9= 2.50743E-02
P 10= 2.49117E-02
P 11= 2.48958E-02
P 12= 2.48930E-02

P 13= 2.48883E-02
P 14= 2.48862E-02
P 15= 2.48849E-02
P 16= 2.48832E-02
P 17= 2.48806E-02
P 18= 2.48805E-02
P 19= 2.48805E-02
P 20= 2.48805E-02
P 21= 2.48805E-02
P 22= 2.48805E-02
P 23= 2.48805E-02
P 24= 2.48805E-02
P 25= 2.48804E-02
P 26= 2.48804E-02
P 27= 2.48804E-02
P 28= 2.48804E-02
P 29= 2.48804E-02
P 30= 2.48804E-02
P 31= 2.48804E-02
P 32= 2.48804E-02
P 33= 2.48804E-02
P 34= 2.48804E-02

FOR RANDOM NUMBER N = 89 P1...PN IS

P 1= 0.965662
P 2= 8.29347E-04
P 3= 7.89706E-04
P 4= 6.27524E-04
P 5= 6.25285E-04
P 6= 5.03357E-04
P 7= 4.59683E-04
P 8= 4.25346E-04
P 9= 4.00651E-04
P 10= 3.72735E-04
P 11= 3.72418E-04
P 12= 3.72221E-04
P 13= 3.72097E-04
P 14= 3.71446E-04
P 15= 3.71061E-04
P 16= 3.70982E-04
P 17= 3.70915E-04
P 18= 3.70890E-04
P 19= 3.70888E-04
P 20= 3.70888E-04
P 21= 3.70885E-04

P 22= 3.70885E-04
P 23= 3.70885E-04
P 24= 3.70885E-04
P 25= 3.70886E-04
P 26= 3.70886E-04
P 27= 3.70887E-04
P 28= 3.70887E-04
P 29= 3.70887E-04
P 30= 3.70887E-04
P 31= 3.70888E-04
P 32= 3.70888E-04
P 33= 3.70889E-04
P 34= 3.70890E-04
P 35= 3.70890E-04
P 36= 3.70890E-04
P 37= 3.70890E-04
P 38= 3.70890E-04
P 39= 3.70890E-04
P 40= 3.70890E-04
P 41= 3.70889E-04
P 42= 3.70890E-04
P 43= 3.70890E-04
P 44= 3.70891E-04
P 45= 3.70891E-04
P 46= 3.70891E-04
P 47= 3.70889E-04
P 48= 3.70890E-04
P 49= 3.70890E-04
P 50= 3.70890E-04
P 51= 3.70890E-04
P 52= 3.70890E-04
P 53= 3.70890E-04
P 54= 3.70890E-04
P 55= 3.70889E-04
P 56= 3.70890E-04
P 57= 3.70890E-04
P 58= 3.70890E-04
P 59= 3.70890E-04
P 60= 3.70890E-04
P 61= 3.70890E-04
P 62= 3.70890E-04
P 63= 3.70889E-04
P 64= 3.70889E-04
P 65= 3.70890E-04
P 66= 3.70890E-04
P 67= 3.70891E-04

P 68= 3.70890E-04
P 69= 3.70889E-04
P 70= 3.70890E-04
P 71= 3.70891E-04
P 72= 3.70891E-04
P 73= 3.70890E-04
P 74= 3.70890E-04
P 75= 3.70891E-04
P 76= 3.70891E-04
P 77= 3.70889E-04
P 78= 3.70890E-04
P 79= 3.70890E-04
P 80= 3.70896E-04
P 81= 3.70894E-04
P 82= 3.70890E-04
P 83= 3.70890E-04
P 84= 3.70887E-04
P 85= 3.70886E-04
P 86= 3.70888E-04
P 87= 3.70896E-04
P 88= 3.70892E-04
P 89= 3.70860E-04

FOR RANDOM NUMBER N = 30 P1...PN IS

P 1= 3.67328E-02
P 2= 3.39521E-02
P 3= 3.34203E-02
P 4= 3.34190E-02
P 5= 3.34171E-02
P 6= 3.34020E-02
P 7= 3.32963E-02
P 8= 3.32670E-02
P 9= 3.32003E-02
P 10= 3.31551E-02
P 11= 3.31488E-02
P 12= 3.31451E-02
P 13= 3.31435E-02
P 14= 3.31375E-02
P 15= 3.31361E-02
P 16= 3.31359E-02
P 17= 3.31356E-02
P 18= 3.31353E-02
P 19= 3.31350E-02
P 20= 3.31350E-02
P 21= 3.31350E-02

P 22= 3.31350E-02
P 23= 3.31350E-02
P 24= 3.31350E-02
P 25= 3.31350E-02
P 26= 3.31350E-02
P 27= 3.31350E-02
P 28= 3.31350E-02
P 29= 3.31350E-02
P 30= 3.31351E-02

FOR RANDOM NUMBER N = 16 P1...PN IS

P 1= 9.12247E-02
P 2= 6.20880E-02
P 3= 6.08716E-02
P 4= 6.06574E-02
P 5= 6.06048E-02
P 6= 6.05893E-02
P 7= 6.05768E-02
P 8= 6.04998E-02
P 9= 6.03837E-02
P 10= 6.03768E-02
P 11= 6.03648E-02
P 12= 6.03630E-02
P 13= 6.03577E-02
P 14= 6.03575E-02
P 15= 6.03432E-02
P 16= 6.03409E-02

FOR RANDOM NUMBER N = 72 P1...PN IS

P 1= 8.48774E-02
P 2= 1.54078E-02
P 3= 1.45301E-02
P 4= 1.40367E-02
P 5= 1.31541E-02
P 6= 1.28253E-02
P 7= 1.28202E-02
P 8= 1.28186E-02
P 9= 1.28095E-02
P 10= 1.28092E-02
P 11= 1.28061E-02
P 12= 1.28055E-02
P 13= 1.28052E-02
P 14= 1.28051E-02
P 15= 1.28050E-02

P 16= 1.28050E-02
P 17= 1.28050E-02
P 18= 1.28050E-02
P 19= 1.28050E-02
P 20= 1.28050E-02
P 21= 1.28050E-02
P 22= 1.28050E-02
P 23= 1.28050E-02
P 24= 1.28050E-02
P 25= 1.28050E-02
P 26= 1.28050E-02
P 27= 1.28050E-02
P 28= 1.28050E-02
P 29= 1.28050E-02
P 30= 1.28050E-02
P 31= 1.28050E-02
P 32= 1.28050E-02
P 33= 1.28050E-02
P 34= 1.28050E-02
P 35= 1.28050E-02
P 36= 1.28050E-02
P 37= 1.28050E-02
P 38= 1.28050E-02
P 39= 1.28050E-02
P 40= 1.28050E-02
P 41= 1.28050E-02
P 42= 1.28050E-02
P 43= 1.28050E-02
P 44= 1.28050E-02
P 45= 1.28050E-02
P 46= 1.28050E-02
P 47= 1.28050E-02
P 48= 1.28050E-02
P 49= 1.28050E-02
P 50= 1.28050E-02
P 51= 1.28050E-02
P 52= 1.28050E-02
P 53= 1.28050E-02
P 54= 1.28050E-02
P 55= 1.28050E-02
P 56= 1.28050E-02
P 57= 1.28050E-02
P 58= 1.28050E-02
P 59= 1.28050E-02
P 60= 1.28050E-02
P 61= 1.28050E-02

P 62= 1.28050E-02
P 63= 1.28050E-02
P 64= 1.28050E-02
P 65= 1.28050E-02
P 66= 1.28050E-02
P 67= 1.28050E-02
P 68= 1.28050E-02
P 69= 1.28050E-02
P 70= 1.28050E-02
P 71= 1.28050E-02
P 72= 1.28050E-02

FOR RANDOM NUMBER N = 75 P1...PN IS

P 1= 2.32286E-02
P 2= 1.54392E-02
P 3= 1.42030E-02
P 4= 1.40494E-02
P 5= 1.34889E-02
P 6= 1.33905E-02
P 7= 1.33891E-02
P 8= 1.33369E-02
P 9= 1.33128E-02
P 10= 1.32878E-02
P 11= 1.31907E-02
P 12= 1.31851E-02
P 13= 1.31621E-02
P 14= 1.31225E-02
P 15= 1.31193E-02
P 16= 1.31191E-02
P 17= 1.31187E-02
P 18= 1.31186E-02
P 19= 1.31184E-02
P 20= 1.31184E-02
P 21= 1.31184E-02
P 22= 1.31183E-02
P 23= 1.31183E-02
P 24= 1.31182E-02
P 25= 1.31182E-02
P 26= 1.31182E-02
P 27= 1.31182E-02
P 28= 1.31182E-02
P 29= 1.31182E-02
P 30= 1.31182E-02
P 31= 1.31182E-02
P 32= 1.31182E-02

P 33= 1.31182E-02
P 34= 1.31182E-02
P 35= 1.31182E-02
P 36= 1.31182E-02
P 37= 1.31182E-02
P 38= 1.31182E-02
P 39= 1.31182E-02
P 40= 1.31182E-02
P 41= 1.31182E-02
P 42= 1.31182E-02
P 43= 1.31182E-02
P 44= 1.31182E-02
P 45= 1.31182E-02
P 46= 1.31182E-02
P 47= 1.31182E-02
P 48= 1.31182E-02
P 49= 1.31182E-02
P 50= 1.31182E-02
P 51= 1.31182E-02
P 52= 1.31182E-02
P 53= 1.31182E-02
P 54= 1.31182E-02
P 55= 1.31182E-02
P 56= 1.31182E-02
P 57= 1.31182E-02
P 58= 1.31182E-02
P 59= 1.31182E-02
P 60= 1.31182E-02
P 61= 1.31182E-02
P 62= 1.31182E-02
P 63= 1.31182E-02
P 64= 1.31182E-02
P 65= 1.31182E-02
P 66= 1.31182E-02
P 67= 1.31182E-02
P 68= 1.31182E-02
P 69= 1.31182E-02
P 70= 1.31182E-02
P 71= 1.31182E-02
P 72= 1.31182E-02
P 73= 1.31182E-02
P 74= 1.31182E-02
P 75= 1.31181E-02

FOR RANDOM NUMBER N = 42 P1...PN IS

P 1= 0.316947
P 2= 0.217452
P 3= 5.15120E-02
P 4= 2.45921E-02
P 5= 2.14838E-02
P 6= 1.19315E-02
P 7= 1.02864E-02
P 8= 1.01518E-02
P 9= 1.01189E-02
P 10= 9.86677E-03
P 11= 9.86667E-03
P 12= 9.86654E-03
P 13= 9.86490E-03
P 14= 9.86426E-03
P 15= 9.86414E-03
P 16= 9.86414E-03
P 17= 9.86414E-03
P 18= 9.86414E-03
P 19= 9.86414E-03
P 20= 9.86414E-03
P 21= 9.86414E-03
P 22= 9.86414E-03
P 23= 9.86413E-03
P 24= 9.86413E-03
P 25= 9.86413E-03
P 26= 9.86413E-03
P 27= 9.86413E-03
P 28= 9.86413E-03
P 29= 9.86413E-03
P 30= 9.86413E-03
P 31= 9.86412E-03
P 32= 9.86412E-03
P 33= 9.86412E-03
P 34= 9.86412E-03
P 35= 9.86412E-03
P 36= 9.86412E-03
P 37= 9.86412E-03
P 38= 9.86412E-03
P 39= 9.86412E-03
P 40= 9.86412E-03
P 41= 9.86412E-03
P 42= 9.86414E-03

FOR RANDOM NUMBER N = 99 P1...PN IS

P 1= 0.483542

P 2= 0.106915
P 3= 1.30104E-02
P 4= 9.51008E-03
P 5= 4.53319E-03
P 6= 4.47683E-03
P 7= 4.43561E-03
P 8= 4.39707E-03
P 9= 4.28473E-03
P 10= 4.13285E-03
P 11= 4.08924E-03
P 12= 4.07581E-03
P 13= 4.07023E-03
P 14= 4.06917E-03
P 15= 4.06351E-03
P 16= 4.05911E-03
P 17= 4.05526E-03
P 18= 4.05412E-03
P 19= 4.05334E-03
P 20= 4.05299E-03
P 21= 4.05230E-03
P 22= 4.05228E-03
P 23= 4.05218E-03
P 24= 4.05218E-03
P 25= 4.05218E-03
P 26= 4.05217E-03
P 27= 4.05215E-03
P 28= 4.05215E-03
P 29= 4.05215E-03
P 30= 4.05215E-03
P 31= 4.05215E-03
P 32= 4.05215E-03
P 33= 4.05215E-03
P 34= 4.05215E-03
P 35= 4.05215E-03
P 36= 4.05215E-03
P 37= 4.05215E-03
P 38= 4.05215E-03
P 39= 4.05215E-03
P 40= 4.05215E-03
P 41= 4.05215E-03
P 42= 4.05214E-03
P 43= 4.05214E-03
P 44= 4.05214E-03
P 45= 4.05214E-03
P 46= 4.05214E-03
P 47= 4.05214E-03

P 48= 4.05214E-03
P 49= 4.05214E-03
P 50= 4.05214E-03
P 51= 4.05214E-03
P 52= 4.05214E-03
P 53= 4.05214E-03
P 54= 4.05214E-03
P 55= 4.05214E-03
P 56= 4.05214E-03
P 57= 4.05214E-03
P 58= 4.05214E-03
P 59= 4.05214E-03
P 60= 4.05214E-03
P 61= 4.05214E-03
P 62= 4.05214E-03
P 63= 4.05213E-03
P 64= 4.05213E-03
P 65= 4.05213E-03
P 66= 4.05213E-03
P 67= 4.05213E-03
P 68= 4.05213E-03
P 69= 4.05213E-03
P 70= 4.05213E-03
P 71= 4.05213E-03
P 72= 4.05213E-03
P 73= 4.05213E-03
P 74= 4.05213E-03
P 75= 4.05213E-03
P 76= 4.05213E-03
P 77= 4.05213E-03
P 78= 4.05213E-03
P 79= 4.05213E-03
P 80= 4.05213E-03
P 81= 4.05213E-03
P 82= 4.05213E-03
P 83= 4.05213E-03
P 84= 4.05213E-03
P 85= 4.05213E-03
P 86= 4.05213E-03
P 87= 4.05213E-03
P 88= 4.05213E-03
P 89= 4.05213E-03
P 90= 4.05213E-03
P 91= 4.05213E-03
P 92= 4.05213E-03
P 93= 4.05214E-03

P 94= 4.05213E-03
P 95= 4.05213E-03
P 96= 4.05213E-03
P 97= 4.05214E-03
P 98= 4.05214E-03
P 99= 4.05210E-03

FOR RANDOM NUMBER N = 25 P1...PN IS

P 1= 0.178113
P 2= 3.56033E-02
P 3= 3.43118E-02
P 4= 3.41808E-02
P 5= 3.41806E-02
P 6= 3.41805E-02
P 7= 3.41805E-02
P 8= 3.41805E-02
P 9= 3.41805E-02
P 10= 3.41805E-02
P 11= 3.41805E-02
P 12= 3.41805E-02
P 13= 3.41805E-02
P 14= 3.41805E-02
P 15= 3.41805E-02
P 16= 3.41805E-02
P 17= 3.41805E-02
P 18= 3.41805E-02
P 19= 3.41805E-02
P 20= 3.41805E-02
P 21= 3.41805E-02
P 22= 3.41805E-02
P 23= 3.41805E-02
P 24= 3.41805E-02
P 25= 3.41805E-02

FOR RANDOM NUMBER N = 85 P1...PN IS

P 1= 2.82240E-02
P 2= 1.35388E-02
P 3= 1.33204E-02
P 4= 1.17591E-02
P 5= 1.16037E-02
P 6= 1.15670E-02
P 7= 1.15286E-02
P 8= 1.15229E-02
P 9= 1.15194E-02

P 10= 1.15188E-02
P 11= 1.15187E-02
P 12= 1.15186E-02
P 13= 1.15186E-02
P 14= 1.15186E-02
P 15= 1.15186E-02
P 16= 1.15186E-02
P 17= 1.15186E-02
P 18= 1.15186E-02
P 19= 1.15186E-02
P 20= 1.15186E-02
P 21= 1.15186E-02
P 22= 1.15186E-02
P 23= 1.15186E-02
P 24= 1.15186E-02
P 25= 1.15186E-02
P 26= 1.15186E-02
P 27= 1.15186E-02
P 28= 1.15186E-02
P 29= 1.15186E-02
P 30= 1.15186E-02
P 31= 1.15186E-02
P 32= 1.15186E-02
P 33= 1.15186E-02
P 34= 1.15186E-02
P 35= 1.15186E-02
P 36= 1.15186E-02
P 37= 1.15186E-02
P 38= 1.15186E-02
P 39= 1.15186E-02
P 40= 1.15186E-02
P 41= 1.15186E-02
P 42= 1.15186E-02
P 43= 1.15186E-02
P 44= 1.15186E-02
P 45= 1.15186E-02
P 46= 1.15186E-02
P 47= 1.15186E-02
P 48= 1.15186E-02
P 49= 1.15186E-02
P 50= 1.15186E-02
P 51= 1.15186E-02
P 52= 1.15186E-02
P 53= 1.15186E-02
P 54= 1.15186E-02
P 55= 1.15186E-02

P 56= 1.15186E-02
P 57= 1.15186E-02
P 58= 1.15186E-02
P 59= 1.15186E-02
P 60= 1.15186E-02
P 61= 1.15186E-02
P 62= 1.15186E-02
P 63= 1.15186E-02
P 64= 1.15186E-02
P 65= 1.15186E-02
P 66= 1.15186E-02
P 67= 1.15186E-02
P 68= 1.15186E-02
P 69= 1.15186E-02
P 70= 1.15186E-02
P 71= 1.15186E-02
P 72= 1.15186E-02
P 73= 1.15186E-02
P 74= 1.15186E-02
P 75= 1.15186E-02
P 76= 1.15186E-02
P 77= 1.15186E-02
P 78= 1.15186E-02
P 79= 1.15186E-02
P 80= 1.15186E-02
P 81= 1.15186E-02
P 82= 1.15186E-02
P 83= 1.15186E-02
P 84= 1.15186E-02
P 85= 1.15186E-02

FOR RANDOM NUMBER N = 32 P1...PN IS

P 1= 0.627018
P 2= 5.51743E-02
P 3= 3.52488E-02
P 4= 1.91953E-02
P 5= 1.38870E-02
P 6= 1.30466E-02
P 7= 1.09369E-02
P 8= 9.27740E-03
P 9= 9.06957E-03
P 10= 9.05644E-03
P 11= 9.03709E-03
P 12= 9.03234E-03
P 13= 9.02110E-03

P 14= 9.00874E-03
P 15= 9.00615E-03
P 16= 9.00476E-03
P 17= 9.00036E-03
P 18= 9.00014E-03
P 19= 8.99908E-03
P 20= 8.99878E-03
P 21= 8.99864E-03
P 22= 8.99841E-03
P 23= 8.99841E-03
P 24= 8.99841E-03
P 25= 8.99841E-03
P 26= 8.99841E-03
P 27= 8.99841E-03
P 28= 8.99841E-03
P 29= 8.99842E-03
P 30= 8.99842E-03
P 31= 8.99842E-03
P 32= 8.99849E-03

FOR RANDOM NUMBER N = 47 P1...PN IS

P 1= 0.182835
P 2= 2.47540E-02
P 3= 2.14321E-02
P 4= 2.09810E-02
P 5= 1.94387E-02
P 6= 1.90353E-02
P 7= 1.77235E-02
P 8= 1.77178E-02
P 9= 1.75609E-02
P 10= 1.74794E-02
P 11= 1.74142E-02
P 12= 1.73285E-02
P 13= 1.73272E-02
P 14= 1.73248E-02
P 15= 1.73239E-02
P 16= 1.73230E-02
P 17= 1.73227E-02
P 18= 1.73227E-02
P 19= 1.73226E-02
P 20= 1.73226E-02
P 21= 1.73226E-02
P 22= 1.73226E-02
P 23= 1.73226E-02
P 24= 1.73226E-02

P 25= 1.73226E-02
P 26= 1.73226E-02
P 27= 1.73226E-02
P 28= 1.73226E-02
P 29= 1.73226E-02
P 30= 1.73226E-02
P 31= 1.73226E-02
P 32= 1.73226E-02
P 33= 1.73226E-02
P 34= 1.73226E-02
P 35= 1.73226E-02
P 36= 1.73226E-02
P 37= 1.73226E-02
P 38= 1.73226E-02
P 39= 1.73226E-02
P 40= 1.73226E-02
P 41= 1.73226E-02
P 42= 1.73226E-02
P 43= 1.73226E-02
P 44= 1.73226E-02
P 45= 1.73226E-02
P 46= 1.73226E-02
P 47= 1.73225E-02

FOR RANDOM NUMBER N = 14 P1...PN IS

P 1= 0.772203
P 2= 0.221866
P 3= 3.33345E-03
P 4= 7.33768E-04
P 5= 2.26197E-04
P 6= 1.83841E-04
P 7= 1.81901E-04
P 8= 1.81781E-04
P 9= 1.81781E-04
P 10= 1.81777E-04
P 11= 1.81772E-04
P 12= 1.81761E-04
P 13= 1.81764E-04
P 14= 1.81769E-04

FOR RANDOM NUMBER N = 33 P1...PN IS

P 1= 3.11887E-02
P 2= 3.09893E-02
P 3= 3.04961E-02

P 4= 3.03259E-02
P 5= 3.03070E-02
P 6= 3.02467E-02
P 7= 3.02450E-02
P 8= 3.02440E-02
P 9= 3.02418E-02
P 10= 3.02394E-02
P 11= 3.02388E-02
P 12= 3.02382E-02
P 13= 3.02381E-02
P 14= 3.02381E-02
P 15= 3.02381E-02
P 16= 3.02380E-02
P 17= 3.02380E-02
P 18= 3.02380E-02
P 19= 3.02380E-02
P 20= 3.02380E-02
P 21= 3.02380E-02
P 22= 3.02380E-02
P 23= 3.02380E-02
P 24= 3.02381E-02
P 25= 3.02381E-02
P 26= 3.02381E-02
P 27= 3.02381E-02
P 28= 3.02381E-02
P 29= 3.02381E-02
P 30= 3.02381E-02
P 31= 3.02381E-02
P 32= 3.02381E-02
P 33= 3.02380E-02

FOR RANDOM NUMBER N = 25 P1...PN IS

P 1= 0.144183
P 2= 9.40778E-02
P 3= 3.43205E-02
P 4= 3.35935E-02
P 5= 3.33247E-02
P 6= 3.32111E-02
P 7= 3.31456E-02
P 8= 3.31120E-02
P 9= 3.30293E-02
P 10= 3.30047E-02
P 11= 3.29998E-02
P 12= 3.29998E-02
P 13= 3.29998E-02

P 14= 3.29998E-02
P 15= 3.29998E-02
P 16= 3.29998E-02
P 17= 3.29998E-02
P 18= 3.29998E-02
P 19= 3.29998E-02
P 20= 3.29998E-02
P 21= 3.29998E-02
P 22= 3.29998E-02
P 23= 3.29998E-02
P 24= 3.29998E-02
P 25= 3.29998E-02

FOR RANDOM NUMBER N = 12 P1...PN IS

P 1= 0.157666
P 2= 8.52603E-02
P 3= 7.95188E-02
P 4= 7.73133E-02
P 5= 7.63671E-02
P 6= 7.61696E-02
P 7= 7.46618E-02
P 8= 7.46436E-02
P 9= 7.46321E-02
P 10= 7.45912E-02
P 11= 7.45900E-02
P 12= 7.45859E-02

FOR RANDOM NUMBER N = 49 P1...PN IS

P 1= 2.71344E-02
P 2= 2.24429E-02
P 3= 2.16190E-02
P 4= 2.10117E-02
P 5= 2.08361E-02
P 6= 2.05617E-02
P 7= 2.02369E-02
P 8= 2.02033E-02
P 9= 2.01703E-02
P 10= 2.01687E-02
P 11= 2.01486E-02
P 12= 2.01467E-02
P 13= 2.01455E-02
P 14= 2.01447E-02
P 15= 2.01445E-02
P 16= 2.01437E-02

P 17= 2.01437E-02
P 18= 2.01437E-02
P 19= 2.01437E-02
P 20= 2.01437E-02
P 21= 2.01437E-02
P 22= 2.01437E-02
P 23= 2.01437E-02
P 24= 2.01437E-02
P 25= 2.01437E-02
P 26= 2.01437E-02
P 27= 2.01437E-02
P 28= 2.01437E-02
P 29= 2.01437E-02
P 30= 2.01437E-02
P 31= 2.01437E-02
P 32= 2.01437E-02
P 33= 2.01437E-02
P 34= 2.01437E-02
P 35= 2.01437E-02
P 36= 2.01437E-02
P 37= 2.01437E-02
P 38= 2.01437E-02
P 39= 2.01437E-02
P 40= 2.01437E-02
P 41= 2.01437E-02
P 42= 2.01437E-02
P 43= 2.01437E-02
P 44= 2.01437E-02
P 45= 2.01437E-02
P 46= 2.01437E-02
P 47= 2.01437E-02
P 48= 2.01437E-02
P 49= 2.01436E-02

FOR RANDOM NUMBER N = 22 P1...PN IS

P 1= 0.178096
P 2= 4.33219E-02
P 3= 4.27201E-02
P 4= 4.09947E-02
P 5= 4.08455E-02
P 6= 3.94386E-02
P 7= 3.90126E-02
P 8= 3.87530E-02
P 9= 3.86129E-02
P 10= 3.84308E-02

P 11= 3.83795E-02
P 12= 3.83332E-02
P 13= 3.83193E-02
P 14= 3.83119E-02
P 15= 3.83083E-02
P 16= 3.83061E-02
P 17= 3.83034E-02
P 18= 3.83033E-02
P 19= 3.83023E-02
P 20= 3.83023E-02
P 21= 3.83023E-02
P 22= 3.83022E-02

FOR RANDOM NUMBER N = 69 P1...PN IS

P 1= 0.282639
P 2= 0.178605
P 3= 0.119680
P 4= 2.10832E-02
P 5= 1.23006E-02
P 6= 8.28118E-03
P 7= 7.28344E-03
P 8= 6.66688E-03
P 9= 6.18376E-03
P 10= 6.18026E-03
P 11= 6.13337E-03
P 12= 5.98827E-03
P 13= 5.95321E-03
P 14= 5.95259E-03
P 15= 5.94954E-03
P 16= 5.94717E-03
P 17= 5.94689E-03
P 18= 5.94674E-03
P 19= 5.94667E-03
P 20= 5.94667E-03
P 21= 5.94667E-03
P 22= 5.94664E-03
P 23= 5.94664E-03
P 24= 5.94664E-03
P 25= 5.94664E-03
P 26= 5.94664E-03
P 27= 5.94664E-03
P 28= 5.94664E-03
P 29= 5.94664E-03
P 30= 5.94664E-03
P 31= 5.94664E-03

P 32= 5.94664E-03
P 33= 5.94664E-03
P 34= 5.94664E-03
P 35= 5.94664E-03
P 36= 5.94664E-03
P 37= 5.94664E-03
P 38= 5.94664E-03
P 39= 5.94664E-03
P 40= 5.94664E-03
P 41= 5.94664E-03
P 42= 5.94664E-03
P 43= 5.94664E-03
P 44= 5.94664E-03
P 45= 5.94664E-03
P 46= 5.94664E-03
P 47= 5.94664E-03
P 48= 5.94664E-03
P 49= 5.94664E-03
P 50= 5.94664E-03
P 51= 5.94664E-03
P 52= 5.94664E-03
P 53= 5.94664E-03
P 54= 5.94664E-03
P 55= 5.94664E-03
P 56= 5.94664E-03
P 57= 5.94664E-03
P 58= 5.94664E-03
P 59= 5.94664E-03
P 60= 5.94664E-03
P 61= 5.94664E-03
P 62= 5.94664E-03
P 63= 5.94664E-03
P 64= 5.94664E-03
P 65= 5.94664E-03
P 66= 5.94664E-03
P 67= 5.94664E-03
P 68= 5.94664E-03
P 69= 5.94664E-03

FOR RANDOM NUMBER N = 78 P1...PN IS

P 1= 5.45351E-02
P 2= 1.98976E-02
P 3= 1.39449E-02
P 4= 1.26214E-02
P 5= 1.22296E-02

P 6= 1.21941E-02
P 7= 1.21844E-02
P 8= 1.21831E-02
P 9= 1.21629E-02
P 10= 1.21469E-02
P 11= 1.21466E-02
P 12= 1.21461E-02
P 13= 1.21457E-02
P 14= 1.21456E-02
P 15= 1.21456E-02
P 16= 1.21456E-02
P 17= 1.21456E-02
P 18= 1.21456E-02
P 19= 1.21456E-02
P 20= 1.21456E-02
P 21= 1.21456E-02
P 22= 1.21456E-02
P 23= 1.21456E-02
P 24= 1.21456E-02
P 25= 1.21456E-02
P 26= 1.21456E-02
P 27= 1.21456E-02
P 28= 1.21456E-02
P 29= 1.21456E-02
P 30= 1.21456E-02
P 31= 1.21456E-02
P 32= 1.21456E-02
P 33= 1.21456E-02
P 34= 1.21456E-02
P 35= 1.21456E-02
P 36= 1.21456E-02
P 37= 1.21456E-02
P 38= 1.21456E-02
P 39= 1.21456E-02
P 40= 1.21456E-02
P 41= 1.21456E-02
P 42= 1.21456E-02
P 43= 1.21456E-02
P 44= 1.21456E-02
P 45= 1.21456E-02
P 46= 1.21456E-02
P 47= 1.21456E-02
P 48= 1.21456E-02
P 49= 1.21456E-02
P 50= 1.21456E-02
P 51= 1.21456E-02

P 52= 1.21456E-02
P 53= 1.21456E-02
P 54= 1.21456E-02
P 55= 1.21456E-02
P 56= 1.21456E-02
P 57= 1.21456E-02
P 58= 1.21455E-02
P 59= 1.21455E-02
P 60= 1.21455E-02
P 61= 1.21455E-02
P 62= 1.21455E-02
P 63= 1.21455E-02
P 64= 1.21455E-02
P 65= 1.21455E-02
P 66= 1.21455E-02
P 67= 1.21455E-02
P 68= 1.21455E-02
P 69= 1.21456E-02
P 70= 1.21456E-02
P 71= 1.21455E-02
P 72= 1.21455E-02
P 73= 1.21455E-02
P 74= 1.21455E-02
P 75= 1.21455E-02
P 76= 1.21455E-02
P 77= 1.21456E-02
P 78= 1.21456E-02

FOR RANDOM NUMBER N = 100 P1...PN IS

P 1= 0.414881
P 2= 2.99036E-02
P 3= 5.87416E-03
P 4= 5.74237E-03
P 5= 5.68682E-03
P 6= 5.67567E-03
P 7= 5.67419E-03
P 8= 5.67380E-03
P 9= 5.66416E-03
P 10= 5.66278E-03
P 11= 5.66265E-03
P 12= 5.66230E-03
P 13= 5.66191E-03
P 14= 5.66179E-03
P 15= 5.66178E-03
P 16= 5.66178E-03

P 17= 5.66178E-03
P 18= 5.66178E-03
P 19= 5.66178E-03
P 20= 5.66178E-03
P 21= 5.66178E-03
P 22= 5.66178E-03
P 23= 5.66178E-03
P 24= 5.66178E-03
P 25= 5.66177E-03
P 26= 5.66177E-03
P 27= 5.66177E-03
P 28= 5.66177E-03
P 29= 5.66177E-03
P 30= 5.66177E-03
P 31= 5.66177E-03
P 32= 5.66177E-03
P 33= 5.66177E-03
P 34= 5.66177E-03
P 35= 5.66177E-03
P 36= 5.66177E-03
P 37= 5.66177E-03
P 38= 5.66177E-03
P 39= 5.66177E-03
P 40= 5.66177E-03
P 41= 5.66177E-03
P 42= 5.66177E-03
P 43= 5.66177E-03
P 44= 5.66177E-03
P 45= 5.66177E-03
P 46= 5.66177E-03
P 47= 5.66177E-03
P 48= 5.66177E-03
P 49= 5.66177E-03
P 50= 5.66177E-03
P 51= 5.66177E-03
P 52= 5.66177E-03
P 53= 5.66177E-03
P 54= 5.66177E-03
P 55= 5.66177E-03
P 56= 5.66177E-03
P 57= 5.66177E-03
P 58= 5.66177E-03
P 59= 5.66177E-03
P 60= 5.66177E-03
P 61= 5.66177E-03
P 62= 5.66176E-03

P 63= 5.66176E-03
P 64= 5.66176E-03
P 65= 5.66176E-03
P 66= 5.66176E-03
P 67= 5.66176E-03
P 68= 5.66176E-03
P 69= 5.66176E-03
P 70= 5.66176E-03
P 71= 5.66176E-03
P 72= 5.66176E-03
P 73= 5.66176E-03
P 74= 5.66176E-03
P 75= 5.66176E-03
P 76= 5.66176E-03
P 77= 5.66176E-03
P 78= 5.66176E-03
P 79= 5.66176E-03
P 80= 5.66176E-03
P 81= 5.66176E-03
P 82= 5.66176E-03
P 83= 5.66176E-03
P 84= 5.66176E-03
P 85= 5.66175E-03
P 86= 5.66175E-03
P 87= 5.66176E-03
P 88= 5.66176E-03
P 89= 5.66176E-03
P 90= 5.66176E-03
P 91= 5.66175E-03
P 92= 5.66175E-03
P 93= 5.66175E-03
P 94= 5.66176E-03
P 95= 5.66176E-03
P 96= 5.66175E-03
P 97= 5.66175E-03
P 98= 5.66176E-03
P 99= 5.66177E-03
P 100= 5.66178E-03

FOR RANDOM NUMBER N = 15 P1...PN IS

P 1= 0.423855
P 2= 0.303955
P 3= 0.236513
P 4= 1.40390E-02
P 5= 2.92971E-03

P 6= 2.22959E-03
P 7= 1.84837E-03
P 8= 1.84040E-03
P 9= 1.83877E-03
P 10= 1.83530E-03
P 11= 1.83353E-03
P 12= 1.82758E-03
P 13= 1.82477E-03
P 14= 1.82358E-03
P 15= 1.80614E-03

FOR RANDOM NUMBER N = 89 P1...PN IS

P 1= 0.322210
P 2= 0.243299
P 3= 0.121036
P 4= 0.106434
P 5= 5.28166E-03
P 6= 3.36763E-03
P 7= 3.26658E-03
P 8= 3.22489E-03
P 9= 3.00141E-03
P 10= 2.65835E-03
P 11= 2.40464E-03
P 12= 2.36297E-03
P 13= 2.35673E-03
P 14= 2.35657E-03
P 15= 2.35656E-03
P 16= 2.35654E-03
P 17= 2.35654E-03
P 18= 2.35654E-03
P 19= 2.35654E-03
P 20= 2.35654E-03
P 21= 2.35654E-03
P 22= 2.35654E-03
P 23= 2.35654E-03
P 24= 2.35654E-03
P 25= 2.35654E-03
P 26= 2.35654E-03
P 27= 2.35654E-03
P 28= 2.35654E-03
P 29= 2.35654E-03
P 30= 2.35654E-03
P 31= 2.35654E-03
P 32= 2.35654E-03
P 33= 2.35654E-03

P 34= 2.35655E-03
P 35= 2.35655E-03
P 36= 2.35655E-03
P 37= 2.35655E-03
P 38= 2.35655E-03
P 39= 2.35655E-03
P 40= 2.35655E-03
P 41= 2.35655E-03
P 42= 2.35655E-03
P 43= 2.35655E-03
P 44= 2.35655E-03
P 45= 2.35655E-03
P 46= 2.35655E-03
P 47= 2.35655E-03
P 48= 2.35655E-03
P 49= 2.35655E-03
P 50= 2.35655E-03
P 51= 2.35655E-03
P 52= 2.35655E-03
P 53= 2.35655E-03
P 54= 2.35655E-03
P 55= 2.35655E-03
P 56= 2.35656E-03
P 57= 2.35656E-03
P 58= 2.35656E-03
P 59= 2.35656E-03
P 60= 2.35656E-03
P 61= 2.35656E-03
P 62= 2.35656E-03
P 63= 2.35656E-03
P 64= 2.35656E-03
P 65= 2.35656E-03
P 66= 2.35656E-03
P 67= 2.35656E-03
P 68= 2.35656E-03
P 69= 2.35656E-03
P 70= 2.35656E-03
P 71= 2.35656E-03
P 72= 2.35656E-03
P 73= 2.35656E-03
P 74= 2.35656E-03
P 75= 2.35656E-03
P 76= 2.35656E-03
P 77= 2.35656E-03
P 78= 2.35656E-03
P 79= 2.35656E-03

P 80= 2.35656E-03
P 81= 2.35656E-03
P 82= 2.35656E-03
P 83= 2.35656E-03
P 84= 2.35656E-03
P 85= 2.35656E-03
P 86= 2.35656E-03
P 87= 2.35655E-03
P 88= 2.35656E-03
P 89= 2.35659E-03

FOR RANDOM NUMBER N = 6 P1...PN IS

P 1= 0.651384
P 2= 0.228445
P 3= 0.115528
P 4= 1.88075E-03
P 5= 1.59035E-03
P 6= 1.17117E-03

FOR RANDOM NUMBER N = 94 P1...PN IS

P 1= 0.438632
P 2= 1.88773E-02
P 3= 8.14383E-03
P 4= 7.53671E-03
P 5= 6.37437E-03
P 6= 6.17819E-03
P 7= 6.03350E-03
P 8= 5.97407E-03
P 9= 5.85195E-03
P 10= 5.84946E-03
P 11= 5.84706E-03
P 12= 5.84039E-03
P 13= 5.83981E-03
P 14= 5.83979E-03
P 15= 5.83979E-03
P 16= 5.83977E-03
P 17= 5.83977E-03
P 18= 5.83977E-03
P 19= 5.83977E-03
P 20= 5.83977E-03
P 21= 5.83977E-03
P 22= 5.83977E-03
P 23= 5.83977E-03
P 24= 5.83977E-03

P 25= 5.83977E-03
P 26= 5.83977E-03
P 27= 5.83977E-03
P 28= 5.83977E-03
P 29= 5.83977E-03
P 30= 5.83977E-03
P 31= 5.83977E-03
P 32= 5.83977E-03
P 33= 5.83977E-03
P 34= 5.83977E-03
P 35= 5.83977E-03
P 36= 5.83977E-03
P 37= 5.83977E-03
P 38= 5.83977E-03
P 39= 5.83977E-03
P 40= 5.83977E-03
P 41= 5.83977E-03
P 42= 5.83977E-03
P 43= 5.83977E-03
P 44= 5.83977E-03
P 45= 5.83977E-03
P 46= 5.83977E-03
P 47= 5.83977E-03
P 48= 5.83977E-03
P 49= 5.83977E-03
P 50= 5.83977E-03
P 51= 5.83977E-03
P 52= 5.83977E-03
P 53= 5.83977E-03
P 54= 5.83977E-03
P 55= 5.83977E-03
P 56= 5.83977E-03
P 57= 5.83977E-03
P 58= 5.83977E-03
P 59= 5.83977E-03
P 60= 5.83977E-03
P 61= 5.83977E-03
P 62= 5.83977E-03
P 63= 5.83977E-03
P 64= 5.83977E-03
P 65= 5.83977E-03
P 66= 5.83977E-03
P 67= 5.83977E-03
P 68= 5.83977E-03
P 69= 5.83977E-03
P 70= 5.83977E-03

P 71= 5.83977E-03
P 72= 5.83977E-03
P 73= 5.83977E-03
P 74= 5.83977E-03
P 75= 5.83977E-03
P 76= 5.83977E-03
P 77= 5.83977E-03
P 78= 5.83977E-03
P 79= 5.83977E-03
P 80= 5.83977E-03
P 81= 5.83977E-03
P 82= 5.83977E-03
P 83= 5.83977E-03
P 84= 5.83977E-03
P 85= 5.83977E-03
P 86= 5.83977E-03
P 87= 5.83977E-03
P 88= 5.83977E-03
P 89= 5.83977E-03
P 90= 5.83977E-03
P 91= 5.83978E-03
P 92= 5.83978E-03
P 93= 5.83979E-03
P 94= 5.83982E-03

FOR RANDOM NUMBER N = 95 P1...PN IS

P 1= 2.41277E-02
P 2= 1.27210E-02
P 3= 1.17896E-02
P 4= 1.13292E-02
P 5= 1.11713E-02
P 6= 1.05830E-02
P 7= 1.03838E-02
P 8= 1.03460E-02
P 9= 1.03399E-02
P 10= 1.03346E-02
P 11= 1.03217E-02
P 12= 1.03173E-02
P 13= 1.03161E-02
P 14= 1.03161E-02
P 15= 1.03161E-02
P 16= 1.03161E-02
P 17= 1.03161E-02
P 18= 1.03161E-02
P 19= 1.03161E-02

P 20= 1.03161E-02
P 21= 1.03161E-02
P 22= 1.03161E-02
P 23= 1.03161E-02
P 24= 1.03161E-02
P 25= 1.03161E-02
P 26= 1.03161E-02
P 27= 1.03161E-02
P 28= 1.03161E-02
P 29= 1.03161E-02
P 30= 1.03161E-02
P 31= 1.03161E-02
P 32= 1.03161E-02
P 33= 1.03161E-02
P 34= 1.03161E-02
P 35= 1.03161E-02
P 36= 1.03161E-02
P 37= 1.03161E-02
P 38= 1.03161E-02
P 39= 1.03161E-02
P 40= 1.03161E-02
P 41= 1.03161E-02
P 42= 1.03161E-02
P 43= 1.03161E-02
P 44= 1.03161E-02
P 45= 1.03161E-02
P 46= 1.03161E-02
P 47= 1.03161E-02
P 48= 1.03161E-02
P 49= 1.03161E-02
P 50= 1.03161E-02
P 51= 1.03161E-02
P 52= 1.03161E-02
P 53= 1.03161E-02
P 54= 1.03161E-02
P 55= 1.03161E-02
P 56= 1.03161E-02
P 57= 1.03161E-02
P 58= 1.03161E-02
P 59= 1.03161E-02
P 60= 1.03161E-02
P 61= 1.03161E-02
P 62= 1.03161E-02
P 63= 1.03161E-02
P 64= 1.03161E-02
P 65= 1.03161E-02

P 66= 1.03161E-02
P 67= 1.03161E-02
P 68= 1.03161E-02
P 69= 1.03161E-02
P 70= 1.03161E-02
P 71= 1.03161E-02
P 72= 1.03161E-02
P 73= 1.03161E-02
P 74= 1.03161E-02
P 75= 1.03161E-02
P 76= 1.03161E-02
P 77= 1.03161E-02
P 78= 1.03161E-02
P 79= 1.03161E-02
P 80= 1.03161E-02
P 81= 1.03161E-02
P 82= 1.03161E-02
P 83= 1.03161E-02
P 84= 1.03161E-02
P 85= 1.03161E-02
P 86= 1.03161E-02
P 87= 1.03161E-02
P 88= 1.03161E-02
P 89= 1.03161E-02
P 90= 1.03161E-02
P 91= 1.03161E-02
P 92= 1.03161E-02
P 93= 1.03161E-02
P 94= 1.03161E-02
P 95= 1.03161E-02

FOR RANDOM NUMBER N = 36 P1...PN IS

P 1= 7.89185E-02
P 2= 3.05622E-02
P 3= 2.77905E-02
P 4= 2.70102E-02
P 5= 2.64222E-02
P 6= 2.63191E-02
P 7= 2.62771E-02
P 8= 2.62413E-02
P 9= 2.61963E-02
P 10= 2.60999E-02
P 11= 2.60943E-02
P 12= 2.60883E-02
P 13= 2.60837E-02

P 14= 2.60833E-02
P 15= 2.60832E-02
P 16= 2.60829E-02
P 17= 2.60829E-02
P 18= 2.60827E-02
P 19= 2.60825E-02
P 20= 2.60825E-02
P 21= 2.60823E-02
P 22= 2.60823E-02
P 23= 2.60823E-02
P 24= 2.60823E-02
P 25= 2.60823E-02
P 26= 2.60823E-02
P 27= 2.60823E-02
P 28= 2.60823E-02
P 29= 2.60823E-02
P 30= 2.60823E-02
P 31= 2.60823E-02
P 32= 2.60823E-02
P 33= 2.60823E-02
P 34= 2.60823E-02
P 35= 2.60823E-02
P 36= 2.60823E-02

FOR RANDOM NUMBER N = 42 P1...PN IS

P 1= 2.86571E-02
P 2= 2.45664E-02
P 3= 2.44222E-02
P 4= 2.41950E-02
P 5= 2.41086E-02
P 6= 2.38957E-02
P 7= 2.37705E-02
P 8= 2.37422E-02
P 9= 2.37037E-02
P 10= 2.36162E-02
P 11= 2.36113E-02
P 12= 2.36050E-02
P 13= 2.36040E-02
P 14= 2.36036E-02
P 15= 2.36036E-02
P 16= 2.36035E-02
P 17= 2.36035E-02
P 18= 2.36035E-02
P 19= 2.36035E-02
P 20= 2.36035E-02

P 21= 2.36035E-02
P 22= 2.36035E-02
P 23= 2.36035E-02
P 24= 2.36035E-02
P 25= 2.36035E-02
P 26= 2.36035E-02
P 27= 2.36035E-02
P 28= 2.36035E-02
P 29= 2.36035E-02
P 30= 2.36035E-02
P 31= 2.36035E-02
P 32= 2.36035E-02
P 33= 2.36035E-02
P 34= 2.36035E-02
P 35= 2.36035E-02
P 36= 2.36035E-02
P 37= 2.36035E-02
P 38= 2.36035E-02
P 39= 2.36035E-02
P 40= 2.36035E-02
P 41= 2.36035E-02
P 42= 2.36035E-02

FOR RANDOM NUMBER N = 98 P1...PN IS

P 1= 5.26328E-02
P 2= 3.19471E-02
P 3= 1.25387E-02
P 4= 1.06095E-02
P 5= 1.01452E-02
P 6= 1.00462E-02
P 7= 9.85039E-03
P 8= 9.54326E-03
P 9= 9.48091E-03
P 10= 9.47658E-03
P 11= 9.47638E-03
P 12= 9.47580E-03
P 13= 9.47501E-03
P 14= 9.47464E-03
P 15= 9.47416E-03
P 16= 9.47416E-03
P 17= 9.47414E-03
P 18= 9.47414E-03
P 19= 9.47414E-03
P 20= 9.47414E-03
P 21= 9.47414E-03

P 22= 9.47414E-03
P 23= 9.47414E-03
P 24= 9.47414E-03
P 25= 9.47414E-03
P 26= 9.47414E-03
P 27= 9.47414E-03
P 28= 9.47414E-03
P 29= 9.47414E-03
P 30= 9.47414E-03
P 31= 9.47414E-03
P 32= 9.47414E-03
P 33= 9.47414E-03
P 34= 9.47414E-03
P 35= 9.47414E-03
P 36= 9.47414E-03
P 37= 9.47414E-03
P 38= 9.47414E-03
P 39= 9.47414E-03
P 40= 9.47414E-03
P 41= 9.47414E-03
P 42= 9.47414E-03
P 43= 9.47414E-03
P 44= 9.47414E-03
P 45= 9.47414E-03
P 46= 9.47414E-03
P 47= 9.47414E-03
P 48= 9.47414E-03
P 49= 9.47414E-03
P 50= 9.47414E-03
P 51= 9.47414E-03
P 52= 9.47414E-03
P 53= 9.47414E-03
P 54= 9.47414E-03
P 55= 9.47414E-03
P 56= 9.47414E-03
P 57= 9.47414E-03
P 58= 9.47414E-03
P 59= 9.47414E-03
P 60= 9.47414E-03
P 61= 9.47413E-03
P 62= 9.47413E-03
P 63= 9.47413E-03
P 64= 9.47413E-03
P 65= 9.47413E-03
P 66= 9.47413E-03
P 67= 9.47413E-03

P 68= 9.47413E-03
P 69= 9.47413E-03
P 70= 9.47413E-03
P 71= 9.47413E-03
P 72= 9.47413E-03
P 73= 9.47413E-03
P 74= 9.47413E-03
P 75= 9.47413E-03
P 76= 9.47413E-03
P 77= 9.47413E-03
P 78= 9.47413E-03
P 79= 9.47413E-03
P 80= 9.47413E-03
P 81= 9.47413E-03
P 82= 9.47413E-03
P 83= 9.47413E-03
P 84= 9.47413E-03
P 85= 9.47412E-03
P 86= 9.47412E-03
P 87= 9.47413E-03
P 88= 9.47413E-03
P 89= 9.47413E-03
P 90= 9.47413E-03
P 91= 9.47413E-03
P 92= 9.47412E-03
P 93= 9.47413E-03
P 94= 9.47413E-03
P 95= 9.47413E-03
P 96= 9.47413E-03
P 97= 9.47413E-03
P 98= 9.47415E-03

FOR RANDOM NUMBER N = 61 P1...PN IS

P 1= 0.922740
P 2= 1.52176E-03
P 3= 1.41377E-03
P 4= 1.35917E-03
P 5= 1.33792E-03
P 6= 1.31300E-03
P 7= 1.29637E-03
P 8= 1.29226E-03
P 9= 1.28338E-03
P 10= 1.27927E-03
P 11= 1.27833E-03
P 12= 1.27779E-03

P 13= 1.27776E-03
P 14= 1.27770E-03
P 15= 1.27770E-03
P 16= 1.27770E-03
P 17= 1.27770E-03
P 18= 1.27770E-03
P 19= 1.27770E-03
P 20= 1.27770E-03
P 21= 1.27770E-03
P 22= 1.27770E-03
P 23= 1.27770E-03
P 24= 1.27770E-03
P 25= 1.27770E-03
P 26= 1.27770E-03
P 27= 1.27771E-03
P 28= 1.27771E-03
P 29= 1.27771E-03
P 30= 1.27771E-03
P 31= 1.27771E-03
P 32= 1.27771E-03
P 33= 1.27771E-03
P 34= 1.27771E-03
P 35= 1.27771E-03
P 36= 1.27771E-03
P 37= 1.27771E-03
P 38= 1.27771E-03
P 39= 1.27771E-03
P 40= 1.27771E-03
P 41= 1.27772E-03
P 42= 1.27772E-03
P 43= 1.27772E-03
P 44= 1.27771E-03
P 45= 1.27771E-03
P 46= 1.27771E-03
P 47= 1.27772E-03
P 48= 1.27772E-03
P 49= 1.27771E-03
P 50= 1.27771E-03
P 51= 1.27772E-03
P 52= 1.27771E-03
P 53= 1.27771E-03
P 54= 1.27771E-03
P 55= 1.27772E-03
P 56= 1.27772E-03
P 57= 1.27771E-03
P 58= 1.27771E-03

P 59= 1.27772E-03
P 60= 1.27771E-03
P 61= 1.27769E-03

FOR RANDOM NUMBER N = 70 P1...PN IS

P 1= 2.91264E-02
P 2= 2.04258E-02
P 3= 1.77947E-02
P 4= 1.52636E-02
P 5= 1.50334E-02
P 6= 1.43885E-02
P 7= 1.40131E-02
P 8= 1.39769E-02
P 9= 1.39658E-02
P 10= 1.39123E-02
P 11= 1.38774E-02
P 12= 1.38726E-02
P 13= 1.38690E-02
P 14= 1.38683E-02
P 15= 1.38681E-02
P 16= 1.38681E-02
P 17= 1.38681E-02
P 18= 1.38681E-02
P 19= 1.38681E-02
P 20= 1.38681E-02
P 21= 1.38681E-02
P 22= 1.38681E-02
P 23= 1.38681E-02
P 24= 1.38681E-02
P 25= 1.38681E-02
P 26= 1.38681E-02
P 27= 1.38681E-02
P 28= 1.38681E-02
P 29= 1.38681E-02
P 30= 1.38681E-02
P 31= 1.38681E-02
P 32= 1.38681E-02
P 33= 1.38681E-02
P 34= 1.38681E-02
P 35= 1.38681E-02
P 36= 1.38681E-02
P 37= 1.38681E-02
P 38= 1.38681E-02
P 39= 1.38681E-02
P 40= 1.38681E-02

P 41= 1.38681E-02
P 42= 1.38681E-02
P 43= 1.38681E-02
P 44= 1.38681E-02
P 45= 1.38681E-02
P 46= 1.38681E-02
P 47= 1.38681E-02
P 48= 1.38681E-02
P 49= 1.38681E-02
P 50= 1.38681E-02
P 51= 1.38681E-02
P 52= 1.38681E-02
P 53= 1.38681E-02
P 54= 1.38681E-02
P 55= 1.38681E-02
P 56= 1.38681E-02
P 57= 1.38681E-02
P 58= 1.38681E-02
P 59= 1.38681E-02
P 60= 1.38681E-02
P 61= 1.38681E-02
P 62= 1.38681E-02
P 63= 1.38681E-02
P 64= 1.38681E-02
P 65= 1.38681E-02
P 66= 1.38681E-02
P 67= 1.38681E-02
P 68= 1.38681E-02
P 69= 1.38681E-02
P 70= 1.38681E-02

FOR RANDOM NUMBER N = 9 P1...PN IS

P 1= 0.732571
P 2= 6.62022E-02
P 3= 3.58682E-02
P 4= 3.45297E-02
P 5= 2.91316E-02
P 6= 2.87284E-02
P 7= 2.82871E-02
P 8= 2.80024E-02
P 9= 1.66790E-02

FOR RANDOM NUMBER N = 16 P1...PN IS

P 1= 9.72012E-02

P 2= 6.20579E-02
P 3= 6.08728E-02
P 4= 6.05107E-02
P 5= 6.00653E-02
P 6= 5.99930E-02
P 7= 5.99660E-02
P 8= 5.99580E-02
P 9= 5.99318E-02
P 10= 5.99241E-02
P 11= 5.99215E-02
P 12= 5.99197E-02
P 13= 5.99196E-02
P 14= 5.99195E-02
P 15= 5.99195E-02
P 16= 5.99193E-02

FOR RANDOM NUMBER N = 93 P1...PN IS

P 1= 5.03638E-02
P 2= 1.54042E-02
P 3= 1.48728E-02
P 4= 1.03329E-02
P 5= 1.02423E-02
P 6= 1.02325E-02
P 7= 1.02221E-02
P 8= 1.02140E-02
P 9= 1.02138E-02
P 10= 1.02135E-02
P 11= 1.02134E-02
P 12= 1.02134E-02
P 13= 1.02133E-02
P 14= 1.02131E-02
P 15= 1.02131E-02
P 16= 1.02131E-02
P 17= 1.02131E-02
P 18= 1.02131E-02
P 19= 1.02131E-02
P 20= 1.02131E-02
P 21= 1.02131E-02
P 22= 1.02131E-02
P 23= 1.02131E-02
P 24= 1.02131E-02
P 25= 1.02131E-02
P 26= 1.02131E-02
P 27= 1.02131E-02
P 28= 1.02131E-02

P 29= 1.02131E-02
P 30= 1.02131E-02
P 31= 1.02131E-02
P 32= 1.02131E-02
P 33= 1.02131E-02
P 34= 1.02131E-02
P 35= 1.02131E-02
P 36= 1.02131E-02
P 37= 1.02131E-02
P 38= 1.02131E-02
P 39= 1.02131E-02
P 40= 1.02131E-02
P 41= 1.02131E-02
P 42= 1.02131E-02
P 43= 1.02131E-02
P 44= 1.02131E-02
P 45= 1.02131E-02
P 46= 1.02131E-02
P 47= 1.02131E-02
P 48= 1.02131E-02
P 49= 1.02131E-02
P 50= 1.02131E-02
P 51= 1.02131E-02
P 52= 1.02131E-02
P 53= 1.02131E-02
P 54= 1.02131E-02
P 55= 1.02131E-02
P 56= 1.02131E-02
P 57= 1.02131E-02
P 58= 1.02131E-02
P 59= 1.02131E-02
P 60= 1.02131E-02
P 61= 1.02131E-02
P 62= 1.02131E-02
P 63= 1.02131E-02
P 64= 1.02131E-02
P 65= 1.02131E-02
P 66= 1.02131E-02
P 67= 1.02131E-02
P 68= 1.02131E-02
P 69= 1.02131E-02
P 70= 1.02131E-02
P 71= 1.02131E-02
P 72= 1.02131E-02
P 73= 1.02131E-02
P 74= 1.02131E-02

P 75= 1.02131E-02
P 76= 1.02131E-02
P 77= 1.02131E-02
P 78= 1.02131E-02
P 79= 1.02131E-02
P 80= 1.02131E-02
P 81= 1.02131E-02
P 82= 1.02131E-02
P 83= 1.02131E-02
P 84= 1.02131E-02
P 85= 1.02131E-02
P 86= 1.02131E-02
P 87= 1.02131E-02
P 88= 1.02131E-02
P 89= 1.02131E-02
P 90= 1.02131E-02
P 91= 1.02131E-02
P 92= 1.02131E-02
P 93= 1.02131E-02

FOR RANDOM NUMBER N = 15 P1...PN IS

P 1= 0.684207
P 2= 0.107779
P 3= 3.67380E-02
P 4= 1.60023E-02
P 5= 1.54849E-02
P 6= 1.39922E-02
P 7= 1.39917E-02
P 8= 1.39804E-02
P 9= 1.39791E-02
P 10= 1.39747E-02
P 11= 1.39742E-02
P 12= 1.39742E-02
P 13= 1.39742E-02
P 14= 1.39742E-02
P 15= 1.39742E-02

FOR RANDOM NUMBER N = 16 P1...PN IS

P 1= 0.406839
P 2= 0.317666
P 3= 3.04625E-02
P 4= 1.96997E-02
P 5= 1.95884E-02
P 6= 1.87888E-02

P 7= 1.87532E-02
P 8= 1.87408E-02
P 9= 1.87399E-02
P 10= 1.86761E-02
P 11= 1.86749E-02
P 12= 1.86744E-02
P 13= 1.86743E-02
P 14= 1.86742E-02
P 15= 1.86739E-02
P 16= 1.86738E-02

FOR RANDOM NUMBER N = 27 P1...PN IS

P 1= 9.56453E-02
P 2= 8.58123E-02
P 3= 4.22819E-02
P 4= 4.18215E-02
P 5= 3.98681E-02
P 6= 3.22608E-02
P 7= 3.19528E-02
P 8= 3.16206E-02
P 9= 3.15665E-02
P 10= 3.15623E-02
P 11= 3.15271E-02
P 12= 3.15123E-02
P 13= 3.15118E-02
P 14= 3.15109E-02
P 15= 3.15038E-02
P 16= 3.15037E-02
P 17= 3.15035E-02
P 18= 3.15035E-02
P 19= 3.15035E-02
P 20= 3.15035E-02
P 21= 3.15035E-02
P 22= 3.15035E-02
P 23= 3.15035E-02
P 24= 3.15035E-02
P 25= 3.15035E-02
P 26= 3.15035E-02
P 27= 3.15034E-02

FOR RANDOM NUMBER N = 74 P1...PN IS

P 1= 0.238961
P 2= 0.121745
P 3= 2.79395E-02

P 4= 2.73385E-02
P 5= 2.47031E-02
P 6= 9.56328E-03
P 7= 8.50809E-03
P 8= 8.18129E-03
P 9= 8.12368E-03
P 10= 8.10794E-03
P 11= 8.10132E-03
P 12= 8.09864E-03
P 13= 8.09016E-03
P 14= 8.07982E-03
P 15= 8.07845E-03
P 16= 8.07568E-03
P 17= 8.07507E-03
P 18= 8.07433E-03
P 19= 8.07428E-03
P 20= 8.07420E-03
P 21= 8.07419E-03
P 22= 8.07418E-03
P 23= 8.07418E-03
P 24= 8.07418E-03
P 25= 8.07418E-03
P 26= 8.07418E-03
P 27= 8.07418E-03
P 28= 8.07418E-03
P 29= 8.07418E-03
P 30= 8.07419E-03
P 31= 8.07419E-03
P 32= 8.07419E-03
P 33= 8.07419E-03
P 34= 8.07419E-03
P 35= 8.07419E-03
P 36= 8.07419E-03
P 37= 8.07419E-03
P 38= 8.07419E-03
P 39= 8.07419E-03
P 40= 8.07419E-03
P 41= 8.07419E-03
P 42= 8.07419E-03
P 43= 8.07419E-03
P 44= 8.07420E-03
P 45= 8.07420E-03
P 46= 8.07419E-03
P 47= 8.07419E-03
P 48= 8.07420E-03
P 49= 8.07420E-03

P 50= 8.07420E-03
P 51= 8.07419E-03
P 52= 8.07419E-03
P 53= 8.07419E-03
P 54= 8.07419E-03
P 55= 8.07419E-03
P 56= 8.07419E-03
P 57= 8.07420E-03
P 58= 8.07420E-03
P 59= 8.07420E-03
P 60= 8.07420E-03
P 61= 8.07419E-03
P 62= 8.07419E-03
P 63= 8.07419E-03
P 64= 8.07420E-03
P 65= 8.07420E-03
P 66= 8.07419E-03
P 67= 8.07419E-03
P 68= 8.07420E-03
P 69= 8.07420E-03
P 70= 8.07419E-03
P 71= 8.07419E-03
P 72= 8.07420E-03
P 73= 8.07420E-03
P 74= 8.07416E-03

FOR RANDOM NUMBER N = 72 P1...PN IS

P 1= 5.79735E-02
P 2= 1.74604E-02
P 3= 1.47609E-02
P 4= 1.32858E-02
P 5= 1.32388E-02
P 6= 1.32234E-02
P 7= 1.32215E-02
P 8= 1.31936E-02
P 9= 1.31864E-02
P 10= 1.31861E-02
P 11= 1.31838E-02
P 12= 1.31834E-02
P 13= 1.31821E-02
P 14= 1.31817E-02
P 15= 1.31817E-02
P 16= 1.31817E-02
P 17= 1.31817E-02
P 18= 1.31817E-02

P 19= 1.31817E-02
P 20= 1.31817E-02
P 21= 1.31817E-02
P 22= 1.31817E-02
P 23= 1.31817E-02
P 24= 1.31817E-02
P 25= 1.31817E-02
P 26= 1.31817E-02
P 27= 1.31817E-02
P 28= 1.31817E-02
P 29= 1.31817E-02
P 30= 1.31817E-02
P 31= 1.31817E-02
P 32= 1.31817E-02
P 33= 1.31817E-02
P 34= 1.31817E-02
P 35= 1.31817E-02
P 36= 1.31817E-02
P 37= 1.31817E-02
P 38= 1.31817E-02
P 39= 1.31817E-02
P 40= 1.31817E-02
P 41= 1.31817E-02
P 42= 1.31817E-02
P 43= 1.31817E-02
P 44= 1.31817E-02
P 45= 1.31817E-02
P 46= 1.31817E-02
P 47= 1.31817E-02
P 48= 1.31817E-02
P 49= 1.31817E-02
P 50= 1.31817E-02
P 51= 1.31817E-02
P 52= 1.31817E-02
P 53= 1.31817E-02
P 54= 1.31817E-02
P 55= 1.31817E-02
P 56= 1.31817E-02
P 57= 1.31817E-02
P 58= 1.31817E-02
P 59= 1.31817E-02
P 60= 1.31817E-02
P 61= 1.31817E-02
P 62= 1.31817E-02
P 63= 1.31817E-02
P 64= 1.31817E-02

P 65= 1.31817E-02
P 66= 1.31817E-02
P 67= 1.31817E-02
P 68= 1.31817E-02
P 69= 1.31817E-02
P 70= 1.31817E-02
P 71= 1.31817E-02
P 72= 1.31817E-02

FOR RANDOM NUMBER N = 78 P1...PN IS

P 1= 3.39639E-02
P 2= 1.54859E-02
P 3= 1.51128E-02
P 4= 1.50683E-02
P 5= 1.36781E-02
P 6= 1.29282E-02
P 7= 1.28188E-02
P 8= 1.27945E-02
P 9= 1.24648E-02
P 10= 1.24139E-02
P 11= 1.24053E-02
P 12= 1.24021E-02
P 13= 1.24010E-02
P 14= 1.24010E-02
P 15= 1.24010E-02
P 16= 1.24010E-02
P 17= 1.24010E-02
P 18= 1.24010E-02
P 19= 1.24010E-02
P 20= 1.24010E-02
P 21= 1.24010E-02
P 22= 1.24010E-02
P 23= 1.24010E-02
P 24= 1.24010E-02
P 25= 1.24010E-02
P 26= 1.24010E-02
P 27= 1.24010E-02
P 28= 1.24010E-02
P 29= 1.24010E-02
P 30= 1.24010E-02
P 31= 1.24010E-02
P 32= 1.24010E-02
P 33= 1.24010E-02
P 34= 1.24010E-02
P 35= 1.24010E-02

P 36= 1.24010E-02
P 37= 1.24010E-02
P 38= 1.24010E-02
P 39= 1.24010E-02
P 40= 1.24010E-02
P 41= 1.24010E-02
P 42= 1.24010E-02
P 43= 1.24010E-02
P 44= 1.24010E-02
P 45= 1.24010E-02
P 46= 1.24010E-02
P 47= 1.24010E-02
P 48= 1.24010E-02
P 49= 1.24010E-02
P 50= 1.24010E-02
P 51= 1.24010E-02
P 52= 1.24010E-02
P 53= 1.24010E-02
P 54= 1.24010E-02
P 55= 1.24010E-02
P 56= 1.24010E-02
P 57= 1.24010E-02
P 58= 1.24010E-02
P 59= 1.24010E-02
P 60= 1.24010E-02
P 61= 1.24010E-02
P 62= 1.24010E-02
P 63= 1.24010E-02
P 64= 1.24010E-02
P 65= 1.24010E-02
P 66= 1.24010E-02
P 67= 1.24010E-02
P 68= 1.24010E-02
P 69= 1.24010E-02
P 70= 1.24010E-02
P 71= 1.24010E-02
P 72= 1.24010E-02
P 73= 1.24010E-02
P 74= 1.24010E-02
P 75= 1.24010E-02
P 76= 1.24010E-02
P 77= 1.24010E-02
P 78= 1.24010E-02

FOR RANDOM NUMBER N = 71 P1...PN IS

P 1=	2.49496E-02
P 2=	1.81227E-02
P 3=	1.59401E-02
P 4=	1.53785E-02
P 5=	1.49071E-02
P 6=	1.42480E-02
P 7=	1.40771E-02
P 8=	1.40013E-02
P 9=	1.39561E-02
P 10=	1.38558E-02
P 11=	1.38136E-02
P 12=	1.37821E-02
P 13=	1.37791E-02
P 14=	1.37791E-02
P 15=	1.37791E-02
P 16=	1.37791E-02
P 17=	1.37791E-02
P 18=	1.37791E-02
P 19=	1.37791E-02
P 20=	1.37791E-02
P 21=	1.37791E-02
P 22=	1.37791E-02
P 23=	1.37791E-02
P 24=	1.37791E-02
P 25=	1.37791E-02
P 26=	1.37791E-02
P 27=	1.37791E-02
P 28=	1.37791E-02
P 29=	1.37791E-02
P 30=	1.37791E-02
P 31=	1.37791E-02
P 32=	1.37791E-02
P 33=	1.37791E-02
P 34=	1.37791E-02
P 35=	1.37791E-02
P 36=	1.37791E-02
P 37=	1.37791E-02
P 38=	1.37791E-02
P 39=	1.37791E-02
P 40=	1.37791E-02
P 41=	1.37791E-02
P 42=	1.37791E-02
P 43=	1.37791E-02
P 44=	1.37791E-02
P 45=	1.37791E-02
P 46=	1.37791E-02

P 47= 1.37791E-02
P 48= 1.37791E-02
P 49= 1.37791E-02
P 50= 1.37791E-02
P 51= 1.37791E-02
P 52= 1.37791E-02
P 53= 1.37791E-02
P 54= 1.37791E-02
P 55= 1.37791E-02
P 56= 1.37791E-02
P 57= 1.37791E-02
P 58= 1.37791E-02
P 59= 1.37791E-02
P 60= 1.37791E-02
P 61= 1.37791E-02
P 62= 1.37791E-02
P 63= 1.37791E-02
P 64= 1.37791E-02
P 65= 1.37791E-02
P 66= 1.37791E-02
P 67= 1.37791E-02
P 68= 1.37791E-02
P 69= 1.37791E-02
P 70= 1.37791E-02
P 71= 1.37791E-02

FOR RANDOM NUMBER N = 56 P1...PN IS

P 1= 6.21242E-02
P 2= 3.89213E-02
P 3= 1.70350E-02
P 4= 1.66421E-02
P 5= 1.66413E-02
P 6= 1.66409E-02
P 7= 1.66404E-02
P 8= 1.66401E-02
P 9= 1.66399E-02
P 10= 1.66399E-02
P 11= 1.66399E-02
P 12= 1.66399E-02
P 13= 1.66399E-02
P 14= 1.66399E-02
P 15= 1.66399E-02
P 16= 1.66399E-02
P 17= 1.66399E-02
P 18= 1.66399E-02

P 19= 1.66399E-02
P 20= 1.66399E-02
P 21= 1.66399E-02
P 22= 1.66399E-02
P 23= 1.66399E-02
P 24= 1.66399E-02
P 25= 1.66399E-02
P 26= 1.66399E-02
P 27= 1.66399E-02
P 28= 1.66399E-02
P 29= 1.66399E-02
P 30= 1.66399E-02
P 31= 1.66399E-02
P 32= 1.66399E-02
P 33= 1.66399E-02
P 34= 1.66399E-02
P 35= 1.66399E-02
P 36= 1.66399E-02
P 37= 1.66399E-02
P 38= 1.66399E-02
P 39= 1.66399E-02
P 40= 1.66399E-02
P 41= 1.66399E-02
P 42= 1.66399E-02
P 43= 1.66399E-02
P 44= 1.66399E-02
P 45= 1.66399E-02
P 46= 1.66399E-02
P 47= 1.66399E-02
P 48= 1.66399E-02
P 49= 1.66399E-02
P 50= 1.66399E-02
P 51= 1.66399E-02
P 52= 1.66399E-02
P 53= 1.66399E-02
P 54= 1.66399E-02
P 55= 1.66399E-02
P 56= 1.66399E-02

FOR RANDOM NUMBER N = 54 P1...PN IS

P 1= 2.42727E-02
P 2= 2.26557E-02
P 3= 1.84393E-02
P 4= 1.83800E-02
P 5= 1.83367E-02

P 6= 1.83269E-02
P 7= 1.83267E-02
P 8= 1.83258E-02
P 9= 1.83253E-02
P 10= 1.83248E-02
P 11= 1.83248E-02
P 12= 1.83247E-02
P 13= 1.83247E-02
P 14= 1.83247E-02
P 15= 1.83247E-02
P 16= 1.83247E-02
P 17= 1.83247E-02
P 18= 1.83247E-02
P 19= 1.83247E-02
P 20= 1.83247E-02
P 21= 1.83247E-02
P 22= 1.83247E-02
P 23= 1.83247E-02
P 24= 1.83247E-02
P 25= 1.83247E-02
P 26= 1.83247E-02
P 27= 1.83247E-02
P 28= 1.83247E-02
P 29= 1.83247E-02
P 30= 1.83247E-02
P 31= 1.83247E-02
P 32= 1.83247E-02
P 33= 1.83247E-02
P 34= 1.83247E-02
P 35= 1.83247E-02
P 36= 1.83247E-02
P 37= 1.83247E-02
P 38= 1.83247E-02
P 39= 1.83247E-02
P 40= 1.83247E-02
P 41= 1.83247E-02
P 42= 1.83247E-02
P 43= 1.83247E-02
P 44= 1.83247E-02
P 45= 1.83247E-02
P 46= 1.83247E-02
P 47= 1.83247E-02
P 48= 1.83247E-02
P 49= 1.83247E-02
P 50= 1.83247E-02
P 51= 1.83247E-02

P 52= 1.83247E-02
P 53= 1.83247E-02
P 54= 1.83247E-02

FOR RANDOM NUMBER N = 84 P1...PN IS

P 1= 0.338054
P 2= 4.79561E-02
P 3= 1.07640E-02
P 4= 1.03784E-02
P 5= 7.94609E-03
P 6= 7.48064E-03
P 7= 7.43515E-03
P 8= 7.40441E-03
P 9= 7.40323E-03
P 10= 7.40269E-03
P 11= 7.40253E-03
P 12= 7.40241E-03
P 13= 7.40238E-03
P 14= 7.40238E-03
P 15= 7.40236E-03
P 16= 7.40236E-03
P 17= 7.40236E-03
P 18= 7.40236E-03
P 19= 7.40236E-03
P 20= 7.40236E-03
P 21= 7.40236E-03
P 22= 7.40236E-03
P 23= 7.40236E-03
P 24= 7.40236E-03
P 25= 7.40236E-03
P 26= 7.40236E-03
P 27= 7.40236E-03
P 28= 7.40236E-03
P 29= 7.40236E-03
P 30= 7.40236E-03
P 31= 7.40236E-03
P 32= 7.40236E-03
P 33= 7.40236E-03
P 34= 7.40236E-03
P 35= 7.40236E-03
P 36= 7.40236E-03
P 37= 7.40236E-03
P 38= 7.40236E-03
P 39= 7.40236E-03
P 40= 7.40236E-03

P 41= 7.40236E-03
P 42= 7.40236E-03
P 43= 7.40236E-03
P 44= 7.40236E-03
P 45= 7.40236E-03
P 46= 7.40236E-03
P 47= 7.40236E-03
P 48= 7.40236E-03
P 49= 7.40236E-03
P 50= 7.40236E-03
P 51= 7.40236E-03
P 52= 7.40236E-03
P 53= 7.40236E-03
P 54= 7.40236E-03
P 55= 7.40236E-03
P 56= 7.40236E-03
P 57= 7.40236E-03
P 58= 7.40236E-03
P 59= 7.40236E-03
P 60= 7.40236E-03
P 61= 7.40236E-03
P 62= 7.40236E-03
P 63= 7.40236E-03
P 64= 7.40236E-03
P 65= 7.40236E-03
P 66= 7.40236E-03
P 67= 7.40236E-03
P 68= 7.40236E-03
P 69= 7.40236E-03
P 70= 7.40236E-03
P 71= 7.40236E-03
P 72= 7.40237E-03
P 73= 7.40237E-03
P 74= 7.40237E-03
P 75= 7.40237E-03
P 76= 7.40237E-03
P 77= 7.40237E-03
P 78= 7.40237E-03
P 79= 7.40237E-03
P 80= 7.40237E-03
P 81= 7.40237E-03
P 82= 7.40237E-03
P 83= 7.40238E-03
P 84= 7.40239E-03

FOR RANDOM NUMBER N = 30 P1...PN IS

P 1= 0.795648
P 2= 0.132031
P 3= 6.61267E-03
P 4= 3.63985E-03
P 5= 3.25430E-03
P 6= 2.43296E-03
P 7= 2.39805E-03
P 8= 2.37784E-03
P 9= 2.34943E-03
P 10= 2.34841E-03
P 11= 2.34583E-03
P 12= 2.34546E-03
P 13= 2.34541E-03
P 14= 2.34536E-03
P 15= 2.34535E-03
P 16= 2.34534E-03
P 17= 2.34533E-03
P 18= 2.34533E-03
P 19= 2.34533E-03
P 20= 2.34533E-03
P 21= 2.34533E-03
P 22= 2.34533E-03
P 23= 2.34533E-03
P 24= 2.34533E-03
P 25= 2.34533E-03
P 26= 2.34533E-03
P 27= 2.34534E-03
P 28= 2.34534E-03
P 29= 2.34534E-03
P 30= 2.34535E-03

FOR RANDOM NUMBER N = 85 P1...PN IS

P 1= 2.06584E-02
P 2= 1.75228E-02
P 3= 1.36481E-02
P 4= 1.24069E-02
P 5= 1.17411E-02
P 6= 1.15900E-02
P 7= 1.15562E-02
P 8= 1.15549E-02
P 9= 1.15517E-02
P 10= 1.15497E-02
P 11= 1.15496E-02
P 12= 1.15496E-02

P 13= 1.15496E-02
P 14= 1.15496E-02
P 15= 1.15496E-02
P 16= 1.15496E-02
P 17= 1.15496E-02
P 18= 1.15496E-02
P 19= 1.15496E-02
P 20= 1.15496E-02
P 21= 1.15496E-02
P 22= 1.15496E-02
P 23= 1.15496E-02
P 24= 1.15496E-02
P 25= 1.15496E-02
P 26= 1.15496E-02
P 27= 1.15496E-02
P 28= 1.15496E-02
P 29= 1.15496E-02
P 30= 1.15496E-02
P 31= 1.15496E-02
P 32= 1.15496E-02
P 33= 1.15496E-02
P 34= 1.15496E-02
P 35= 1.15496E-02
P 36= 1.15496E-02
P 37= 1.15496E-02
P 38= 1.15496E-02
P 39= 1.15496E-02
P 40= 1.15496E-02
P 41= 1.15496E-02
P 42= 1.15496E-02
P 43= 1.15496E-02
P 44= 1.15496E-02
P 45= 1.15496E-02
P 46= 1.15496E-02
P 47= 1.15496E-02
P 48= 1.15496E-02
P 49= 1.15496E-02
P 50= 1.15496E-02
P 51= 1.15496E-02
P 52= 1.15496E-02
P 53= 1.15496E-02
P 54= 1.15496E-02
P 55= 1.15496E-02
P 56= 1.15496E-02
P 57= 1.15496E-02
P 58= 1.15496E-02

P 59= 1.15496E-02
P 60= 1.15496E-02
P 61= 1.15496E-02
P 62= 1.15496E-02
P 63= 1.15496E-02
P 64= 1.15496E-02
P 65= 1.15496E-02
P 66= 1.15496E-02
P 67= 1.15496E-02
P 68= 1.15496E-02
P 69= 1.15496E-02
P 70= 1.15496E-02
P 71= 1.15496E-02
P 72= 1.15496E-02
P 73= 1.15496E-02
P 74= 1.15496E-02
P 75= 1.15496E-02
P 76= 1.15496E-02
P 77= 1.15496E-02
P 78= 1.15496E-02
P 79= 1.15496E-02
P 80= 1.15496E-02
P 81= 1.15496E-02
P 82= 1.15496E-02
P 83= 1.15496E-02
P 84= 1.15496E-02
P 85= 1.15496E-02

FOR RANDOM NUMBER N = 43 P1...PN IS

P 1= 0.312916
P 2= 0.166020
P 3= 2.73792E-02
P 4= 2.40787E-02
P 5= 2.39088E-02
P 6= 1.71776E-02
P 7= 1.40250E-02
P 8= 1.27304E-02
P 9= 1.20410E-02
P 10= 1.15165E-02
P 11= 1.14772E-02
P 12= 1.14639E-02
P 13= 1.14633E-02
P 14= 1.14612E-02
P 15= 1.14610E-02
P 16= 1.14601E-02

P 17= 1.14600E-02
P 18= 1.14600E-02
P 19= 1.14600E-02
P 20= 1.14600E-02
P 21= 1.14600E-02
P 22= 1.14600E-02
P 23= 1.14600E-02
P 24= 1.14600E-02
P 25= 1.14600E-02
P 26= 1.14600E-02
P 27= 1.14600E-02
P 28= 1.14600E-02
P 29= 1.14600E-02
P 30= 1.14600E-02
P 31= 1.14600E-02
P 32= 1.14600E-02
P 33= 1.14600E-02
P 34= 1.14600E-02
P 35= 1.14600E-02
P 36= 1.14600E-02
P 37= 1.14600E-02
P 38= 1.14600E-02
P 39= 1.14600E-02
P 40= 1.14600E-02
P 41= 1.14600E-02
P 42= 1.14600E-02
P 43= 1.14599E-02

FOR RANDOM NUMBER N = 78 P1...PN IS

P 1= 0.443251
P 2= 0.128056
P 3= 1.00789E-01
P 4= 6.22254E-02
P 5= 4.77723E-02
P 6= 4.24573E-02
P 7= 3.35627E-03
P 8= 3.23055E-03
P 9= 2.60870E-03
P 10= 2.49867E-03
P 11= 2.44753E-03
P 12= 2.42283E-03
P 13= 2.40938E-03
P 14= 2.40769E-03
P 15= 2.40751E-03
P 16= 2.40746E-03

P 17= 2.40742E-03
P 18= 2.40731E-03
P 19= 2.40730E-03
P 20= 2.40730E-03
P 21= 2.40730E-03
P 22= 2.40730E-03
P 23= 2.40730E-03
P 24= 2.40730E-03
P 25= 2.40730E-03
P 26= 2.40730E-03
P 27= 2.40730E-03
P 28= 2.40730E-03
P 29= 2.40730E-03
P 30= 2.40730E-03
P 31= 2.40730E-03
P 32= 2.40730E-03
P 33= 2.40730E-03
P 34= 2.40730E-03
P 35= 2.40730E-03
P 36= 2.40730E-03
P 37= 2.40730E-03
P 38= 2.40730E-03
P 39= 2.40730E-03
P 40= 2.40730E-03
P 41= 2.40730E-03
P 42= 2.40730E-03
P 43= 2.40730E-03
P 44= 2.40730E-03
P 45= 2.40730E-03
P 46= 2.40730E-03
P 47= 2.40730E-03
P 48= 2.40730E-03
P 49= 2.40730E-03
P 50= 2.40730E-03
P 51= 2.40730E-03
P 52= 2.40730E-03
P 53= 2.40729E-03
P 54= 2.40729E-03
P 55= 2.40729E-03
P 56= 2.40729E-03
P 57= 2.40729E-03
P 58= 2.40729E-03
P 59= 2.40729E-03
P 60= 2.40729E-03
P 61= 2.40729E-03
P 62= 2.40728E-03

P 63= 2.40728E-03
P 64= 2.40728E-03
P 65= 2.40728E-03
P 66= 2.40728E-03
P 67= 2.40728E-03
P 68= 2.40728E-03
P 69= 2.40728E-03
P 70= 2.40728E-03
P 71= 2.40728E-03
P 72= 2.40728E-03
P 73= 2.40728E-03
P 74= 2.40728E-03
P 75= 2.40728E-03
P 76= 2.40727E-03
P 77= 2.40728E-03
P 78= 2.40730E-03

FOR RANDOM NUMBER N = 10 P1...PN IS

P 1= 0.242075
P 2= 1.01472E-01
P 3= 9.17159E-02
P 4= 8.73062E-02
P 5= 8.20701E-02
P 6= 8.18248E-02
P 7= 8.18227E-02
P 8= 7.73482E-02
P 9= 7.73398E-02
P 10= 7.70251E-02

FOR RANDOM NUMBER N = 15 P1...PN IS

P 1= 0.602515
P 2= 9.93517E-02
P 3= 5.58171E-02
P 4= 2.32712E-02
P 5= 2.14299E-02
P 6= 2.10288E-02
P 7= 2.07531E-02
P 8= 2.06454E-02
P 9= 1.94097E-02
P 10= 1.93304E-02
P 11= 1.93077E-02
P 12= 1.92956E-02
P 13= 1.92839E-02
P 14= 1.92803E-02

P 15= 1.92800E-02

FOR RANDOM NUMBER N = 66 P1...PN IS

P 1= 8.39461E-02
P 2= 2.26326E-02
P 3= 1.40728E-02
P 4= 1.39623E-02
P 5= 1.39592E-02
P 6= 1.39582E-02
P 7= 1.39581E-02
P 8= 1.39580E-02
P 9= 1.39578E-02
P 10= 1.39578E-02
P 11= 1.39578E-02
P 12= 1.39578E-02
P 13= 1.39578E-02
P 14= 1.39578E-02
P 15= 1.39578E-02
P 16= 1.39578E-02
P 17= 1.39578E-02
P 18= 1.39578E-02
P 19= 1.39578E-02
P 20= 1.39578E-02
P 21= 1.39578E-02
P 22= 1.39578E-02
P 23= 1.39578E-02
P 24= 1.39578E-02
P 25= 1.39578E-02
P 26= 1.39578E-02
P 27= 1.39578E-02
P 28= 1.39578E-02
P 29= 1.39578E-02
P 30= 1.39578E-02
P 31= 1.39578E-02
P 32= 1.39578E-02
P 33= 1.39578E-02
P 34= 1.39578E-02
P 35= 1.39578E-02
P 36= 1.39578E-02
P 37= 1.39578E-02
P 38= 1.39578E-02
P 39= 1.39578E-02
P 40= 1.39578E-02
P 41= 1.39578E-02
P 42= 1.39578E-02

P 43= 1.39578E-02
P 44= 1.39578E-02
P 45= 1.39578E-02
P 46= 1.39578E-02
P 47= 1.39578E-02
P 48= 1.39578E-02
P 49= 1.39578E-02
P 50= 1.39578E-02
P 51= 1.39578E-02
P 52= 1.39578E-02
P 53= 1.39578E-02
P 54= 1.39578E-02
P 55= 1.39578E-02
P 56= 1.39578E-02
P 57= 1.39578E-02
P 58= 1.39578E-02
P 59= 1.39578E-02
P 60= 1.39578E-02
P 61= 1.39578E-02
P 62= 1.39578E-02
P 63= 1.39578E-02
P 64= 1.39578E-02
P 65= 1.39578E-02
P 66= 1.39578E-02

FOR RANDOM NUMBER N = 98 P1...PN IS

P 1= 3.25391E-02
P 2= 1.04841E-02
P 3= 1.03997E-02
P 4= 1.00244E-02
P 5= 9.97589E-03
P 6= 9.96784E-03
P 7= 9.96334E-03
P 8= 9.96317E-03
P 9= 9.96314E-03
P 10= 9.96314E-03
P 11= 9.96314E-03
P 12= 9.96314E-03
P 13= 9.96314E-03
P 14= 9.96314E-03
P 15= 9.96314E-03
P 16= 9.96314E-03
P 17= 9.96314E-03
P 18= 9.96314E-03
P 19= 9.96314E-03

P 20= 9.96314E-03
P 21= 9.96314E-03
P 22= 9.96314E-03
P 23= 9.96314E-03
P 24= 9.96314E-03
P 25= 9.96314E-03
P 26= 9.96314E-03
P 27= 9.96314E-03
P 28= 9.96314E-03
P 29= 9.96314E-03
P 30= 9.96314E-03
P 31= 9.96314E-03
P 32= 9.96314E-03
P 33= 9.96314E-03
P 34= 9.96314E-03
P 35= 9.96314E-03
P 36= 9.96314E-03
P 37= 9.96314E-03
P 38= 9.96314E-03
P 39= 9.96314E-03
P 40= 9.96314E-03
P 41= 9.96314E-03
P 42= 9.96314E-03
P 43= 9.96314E-03
P 44= 9.96314E-03
P 45= 9.96314E-03
P 46= 9.96314E-03
P 47= 9.96314E-03
P 48= 9.96314E-03
P 49= 9.96314E-03
P 50= 9.96314E-03
P 51= 9.96314E-03
P 52= 9.96314E-03
P 53= 9.96314E-03
P 54= 9.96314E-03
P 55= 9.96314E-03
P 56= 9.96314E-03
P 57= 9.96314E-03
P 58= 9.96314E-03
P 59= 9.96314E-03
P 60= 9.96314E-03
P 61= 9.96314E-03
P 62= 9.96314E-03
P 63= 9.96314E-03
P 64= 9.96313E-03
P 65= 9.96313E-03

P 66= 9.96313E-03
P 67= 9.96313E-03
P 68= 9.96313E-03
P 69= 9.96313E-03
P 70= 9.96313E-03
P 71= 9.96313E-03
P 72= 9.96313E-03
P 73= 9.96313E-03
P 74= 9.96312E-03
P 75= 9.96312E-03
P 76= 9.96312E-03
P 77= 9.96312E-03
P 78= 9.96312E-03
P 79= 9.96312E-03
P 80= 9.96312E-03
P 81= 9.96313E-03
P 82= 9.96313E-03
P 83= 9.96313E-03
P 84= 9.96312E-03
P 85= 9.96312E-03
P 86= 9.96313E-03
P 87= 9.96313E-03
P 88= 9.96312E-03
P 89= 9.96312E-03
P 90= 9.96313E-03
P 91= 9.96312E-03
P 92= 9.96312E-03
P 93= 9.96312E-03
P 94= 9.96313E-03
P 95= 9.96313E-03
P 96= 9.96313E-03
P 97= 9.96310E-03
P 98= 9.96310E-03

FOR RANDOM NUMBER N = 17 P1...PN IS

P 1= 0.305765
P 2= 8.46676E-02
P 3= 4.57127E-02
P 4= 4.34388E-02
P 5= 4.23826E-02
P 6= 4.19260E-02
P 7= 4.02470E-02
P 8= 3.95864E-02
P 9= 3.95862E-02
P 10= 3.95860E-02

P 11= 3.95860E-02
P 12= 3.95860E-02
P 13= 3.95860E-02
P 14= 3.95860E-02
P 15= 3.95860E-02
P 16= 3.95860E-02
P 17= 3.95859E-02

FOR RANDOM NUMBER N = 31 P1...PN IS

P 1= 4.93082E-02
P 2= 3.26134E-02
P 3= 3.19771E-02
P 4= 3.19643E-02
P 5= 3.17725E-02
P 6= 3.17411E-02
P 7= 3.17125E-02
P 8= 3.16408E-02
P 9= 3.16341E-02
P 10= 3.16201E-02
P 11= 3.16199E-02
P 12= 3.16198E-02
P 13= 3.16198E-02
P 14= 3.16198E-02
P 15= 3.16198E-02
P 16= 3.16198E-02
P 17= 3.16198E-02
P 18= 3.16198E-02
P 19= 3.16198E-02
P 20= 3.16198E-02
P 21= 3.16198E-02
P 22= 3.16198E-02
P 23= 3.16198E-02
P 24= 3.16198E-02
P 25= 3.16198E-02
P 26= 3.16198E-02
P 27= 3.16198E-02
P 28= 3.16198E-02
P 29= 3.16198E-02
P 30= 3.16198E-02
P 31= 3.16198E-02

FOR RANDOM NUMBER N = 8 P1...PN IS

P 1= 0.490125
P 2= 0.244063

P 3= 0.197808
P 4= 4.26999E-02
P 5= 6.34619E-03
P 6= 6.32483E-03
P 7= 6.31727E-03
P 8= 6.31577E-03

FOR RANDOM NUMBER N = 15 P1...PN IS

P 1= 0.381914
P 2= 0.216701
P 3= 9.31300E-02
P 4= 6.10813E-02
P 5= 4.39750E-02
P 6= 3.73412E-02
P 7= 2.80298E-02
P 8= 1.81996E-02
P 9= 1.81921E-02
P 10= 1.71992E-02
P 11= 1.70738E-02
P 12= 1.70067E-02
P 13= 1.69403E-02
P 14= 1.69118E-02
P 15= 1.63044E-02

FOR RANDOM NUMBER N = 32 P1...PN IS

P 1= 0.187658
P 2= 6.21987E-02
P 3= 3.31642E-02
P 4= 2.64261E-02
P 5= 2.47515E-02
P 6= 2.46626E-02
P 7= 2.46625E-02
P 8= 2.46617E-02
P 9= 2.46616E-02
P 10= 2.46591E-02
P 11= 2.46589E-02
P 12= 2.46588E-02
P 13= 2.46588E-02
P 14= 2.46588E-02
P 15= 2.46588E-02
P 16= 2.46588E-02
P 17= 2.46588E-02
P 18= 2.46588E-02
P 19= 2.46588E-02

P 20= 2.46588E-02
P 21= 2.46588E-02
P 22= 2.46588E-02
P 23= 2.46588E-02
P 24= 2.46588E-02
P 25= 2.46588E-02
P 26= 2.46588E-02
P 27= 2.46588E-02
P 28= 2.46588E-02
P 29= 2.46588E-02
P 30= 2.46588E-02
P 31= 2.46588E-02
P 32= 2.46587E-02

FOR RANDOM NUMBER N = 99 P1...PN IS

P 1= 3.77887E-02
P 2= 3.48979E-02
P 3= 2.86196E-02
P 4= 1.62697E-02
P 5= 1.31908E-02
P 6= 1.09916E-02
P 7= 1.03917E-02
P 8= 9.39820E-03
P 9= 9.35568E-03
P 10= 9.29096E-03
P 11= 9.25640E-03
P 12= 9.24157E-03
P 13= 9.22805E-03
P 14= 9.22344E-03
P 15= 9.21164E-03
P 16= 9.21084E-03
P 17= 9.21052E-03
P 18= 9.21005E-03
P 19= 9.21003E-03
P 20= 9.21003E-03
P 21= 9.21003E-03
P 22= 9.21003E-03
P 23= 9.21003E-03
P 24= 9.21003E-03
P 25= 9.21003E-03
P 26= 9.21003E-03
P 27= 9.21003E-03
P 28= 9.21003E-03
P 29= 9.21003E-03
P 30= 9.21003E-03

P 31= 9.21003E-03
P 32= 9.21003E-03
P 33= 9.21003E-03
P 34= 9.21003E-03
P 35= 9.21003E-03
P 36= 9.21003E-03
P 37= 9.21003E-03
P 38= 9.21003E-03
P 39= 9.21004E-03
P 40= 9.21004E-03
P 41= 9.21004E-03
P 42= 9.21003E-03
P 43= 9.21003E-03
P 44= 9.21004E-03
P 45= 9.21004E-03
P 46= 9.21003E-03
P 47= 9.21003E-03
P 48= 9.21003E-03
P 49= 9.21003E-03
P 50= 9.21003E-03
P 51= 9.21003E-03
P 52= 9.21003E-03
P 53= 9.21003E-03
P 54= 9.21003E-03
P 55= 9.21003E-03
P 56= 9.21003E-03
P 57= 9.21003E-03
P 58= 9.21003E-03
P 59= 9.21003E-03
P 60= 9.21003E-03
P 61= 9.21003E-03
P 62= 9.21003E-03
P 63= 9.21003E-03
P 64= 9.21003E-03
P 65= 9.21003E-03
P 66= 9.21003E-03
P 67= 9.21003E-03
P 68= 9.21002E-03
P 69= 9.21002E-03
P 70= 9.21002E-03
P 71= 9.21002E-03
P 72= 9.21002E-03
P 73= 9.21002E-03
P 74= 9.21002E-03
P 75= 9.21002E-03
P 76= 9.21002E-03

P 77= 9.21002E-03
P 78= 9.21002E-03
P 79= 9.21002E-03
P 80= 9.21002E-03
P 81= 9.21002E-03
P 82= 9.21002E-03
P 83= 9.21002E-03
P 84= 9.21002E-03
P 85= 9.21002E-03
P 86= 9.21002E-03
P 87= 9.21002E-03
P 88= 9.21002E-03
P 89= 9.21002E-03
P 90= 9.21002E-03
P 91= 9.21002E-03
P 92= 9.21002E-03
P 93= 9.21002E-03
P 94= 9.21002E-03
P 95= 9.21002E-03
P 96= 9.21002E-03
P 97= 9.21003E-03
P 98= 9.21002E-03
P 99= 9.20999E-03

FOR RANDOM NUMBER N = 64 P1...PN IS

P 1= 3.39051E-02
P 2= 2.10389E-02
P 3= 1.54705E-02
P 4= 1.53811E-02
P 5= 1.53277E-02
P 6= 1.52460E-02
P 7= 1.52420E-02
P 8= 1.52380E-02
P 9= 1.52377E-02
P 10= 1.52351E-02
P 11= 1.52350E-02
P 12= 1.52349E-02
P 13= 1.52349E-02
P 14= 1.52349E-02
P 15= 1.52348E-02
P 16= 1.52348E-02
P 17= 1.52348E-02
P 18= 1.52348E-02
P 19= 1.52348E-02
P 20= 1.52348E-02

P 21= 1.52348E-02
P 22= 1.52348E-02
P 23= 1.52348E-02
P 24= 1.52348E-02
P 25= 1.52348E-02
P 26= 1.52348E-02
P 27= 1.52348E-02
P 28= 1.52348E-02
P 29= 1.52348E-02
P 30= 1.52348E-02
P 31= 1.52348E-02
P 32= 1.52348E-02
P 33= 1.52348E-02
P 34= 1.52348E-02
P 35= 1.52348E-02
P 36= 1.52348E-02
P 37= 1.52348E-02
P 38= 1.52348E-02
P 39= 1.52348E-02
P 40= 1.52348E-02
P 41= 1.52348E-02
P 42= 1.52348E-02
P 43= 1.52348E-02
P 44= 1.52348E-02
P 45= 1.52348E-02
P 46= 1.52348E-02
P 47= 1.52348E-02
P 48= 1.52348E-02
P 49= 1.52348E-02
P 50= 1.52348E-02
P 51= 1.52348E-02
P 52= 1.52348E-02
P 53= 1.52347E-02
P 54= 1.52347E-02
P 55= 1.52347E-02
P 56= 1.52347E-02
P 57= 1.52347E-02
P 58= 1.52347E-02
P 59= 1.52347E-02
P 60= 1.52347E-02
P 61= 1.52347E-02
P 62= 1.52347E-02
P 63= 1.52347E-02
P 64= 1.52347E-02

FOR RANDOM NUMBER N = 42 P1...PN IS

P 1= 0.174256
P 2= 6.54370E-02
P 3= 2.07927E-02
P 4= 2.01126E-02
P 5= 1.93567E-02
P 6= 1.93056E-02
P 7= 1.90983E-02
P 8= 1.90707E-02
P 9= 1.89593E-02
P 10= 1.89180E-02
P 11= 1.89166E-02
P 12= 1.89088E-02
P 13= 1.89019E-02
P 14= 1.88985E-02
P 15= 1.88984E-02
P 16= 1.88977E-02
P 17= 1.88976E-02
P 18= 1.88951E-02
P 19= 1.88950E-02
P 20= 1.88950E-02
P 21= 1.88950E-02
P 22= 1.88950E-02
P 23= 1.88949E-02
P 24= 1.88949E-02
P 25= 1.88949E-02
P 26= 1.88949E-02
P 27= 1.88949E-02
P 28= 1.88949E-02
P 29= 1.88949E-02
P 30= 1.88949E-02
P 31= 1.88949E-02
P 32= 1.88949E-02
P 33= 1.88949E-02
P 34= 1.88949E-02
P 35= 1.88949E-02
P 36= 1.88949E-02
P 37= 1.88949E-02
P 38= 1.88949E-02
P 39= 1.88949E-02
P 40= 1.88949E-02
P 41= 1.88949E-02
P 42= 1.88949E-02

FOR RANDOM NUMBER N = 78 P1...PN IS

P 1= 4.33570E-02
P 2= 1.82246E-02
P 3= 1.31511E-02
P 4= 1.27659E-02
P 5= 1.26036E-02
P 6= 1.24813E-02
P 7= 1.24684E-02
P 8= 1.24385E-02
P 9= 1.23446E-02
P 10= 1.23230E-02
P 11= 1.23221E-02
P 12= 1.23221E-02
P 13= 1.23218E-02
P 14= 1.23215E-02
P 15= 1.23212E-02
P 16= 1.23212E-02
P 17= 1.23212E-02
P 18= 1.23212E-02
P 19= 1.23212E-02
P 20= 1.23212E-02
P 21= 1.23212E-02
P 22= 1.23212E-02
P 23= 1.23212E-02
P 24= 1.23212E-02
P 25= 1.23212E-02
P 26= 1.23212E-02
P 27= 1.23212E-02
P 28= 1.23212E-02
P 29= 1.23212E-02
P 30= 1.23212E-02
P 31= 1.23212E-02
P 32= 1.23212E-02
P 33= 1.23212E-02
P 34= 1.23212E-02
P 35= 1.23212E-02
P 36= 1.23212E-02
P 37= 1.23212E-02
P 38= 1.23212E-02
P 39= 1.23212E-02
P 40= 1.23212E-02
P 41= 1.23212E-02
P 42= 1.23212E-02
P 43= 1.23212E-02
P 44= 1.23212E-02
P 45= 1.23212E-02
P 46= 1.23212E-02

P 47= 1.23212E-02
P 48= 1.23212E-02
P 49= 1.23212E-02
P 50= 1.23212E-02
P 51= 1.23212E-02
P 52= 1.23212E-02
P 53= 1.23212E-02
P 54= 1.23212E-02
P 55= 1.23212E-02
P 56= 1.23212E-02
P 57= 1.23211E-02
P 58= 1.23211E-02
P 59= 1.23211E-02
P 60= 1.23211E-02
P 61= 1.23211E-02
P 62= 1.23211E-02
P 63= 1.23211E-02
P 64= 1.23211E-02
P 65= 1.23211E-02
P 66= 1.23211E-02
P 67= 1.23211E-02
P 68= 1.23211E-02
P 69= 1.23211E-02
P 70= 1.23211E-02
P 71= 1.23211E-02
P 72= 1.23211E-02
P 73= 1.23211E-02
P 74= 1.23211E-02
P 75= 1.23211E-02
P 76= 1.23211E-02
P 77= 1.23211E-02
P 78= 1.23211E-02

FOR RANDOM NUMBER N = 53 P1...PN IS

P 1= 0.632882
P 2= 4.94151E-02
P 3= 3.81753E-02
P 4= 2.06192E-02
P 5= 1.79242E-02
P 6= 9.81953E-03
P 7= 5.16432E-03
P 8= 5.07719E-03
P 9= 5.01029E-03
P 10= 4.93975E-03
P 11= 4.92191E-03

P 12= 4.90784E-03
P 13= 4.90770E-03
P 14= 4.90687E-03
P 15= 4.90620E-03
P 16= 4.90590E-03
P 17= 4.90587E-03
P 18= 4.90586E-03
P 19= 4.90586E-03
P 20= 4.90586E-03
P 21= 4.90585E-03
P 22= 4.90585E-03
P 23= 4.90585E-03
P 24= 4.90585E-03
P 25= 4.90585E-03
P 26= 4.90585E-03
P 27= 4.90585E-03
P 28= 4.90585E-03
P 29= 4.90585E-03
P 30= 4.90585E-03
P 31= 4.90585E-03
P 32= 4.90585E-03
P 33= 4.90585E-03
P 34= 4.90585E-03
P 35= 4.90585E-03
P 36= 4.90585E-03
P 37= 4.90585E-03
P 38= 4.90585E-03
P 39= 4.90585E-03
P 40= 4.90585E-03
P 41= 4.90585E-03
P 42= 4.90585E-03
P 43= 4.90585E-03
P 44= 4.90585E-03
P 45= 4.90585E-03
P 46= 4.90585E-03
P 47= 4.90585E-03
P 48= 4.90585E-03
P 49= 4.90584E-03
P 50= 4.90585E-03
P 51= 4.90586E-03
P 52= 4.90586E-03
P 53= 4.90582E-03

FOR RANDOM NUMBER N = 66 P1...PN IS

P 1= 0.152714

P 2= 5.10603E-02
P 3= 1.34099E-02
P 4= 1.32213E-02
P 5= 1.31516E-02
P 6= 1.31494E-02
P 7= 1.31368E-02
P 8= 1.25269E-02
P 9= 1.23812E-02
P 10= 1.23798E-02
P 11= 1.23762E-02
P 12= 1.23755E-02
P 13= 1.23727E-02
P 14= 1.23727E-02
P 15= 1.23726E-02
P 16= 1.23726E-02
P 17= 1.23725E-02
P 18= 1.23725E-02
P 19= 1.23725E-02
P 20= 1.23725E-02
P 21= 1.23725E-02
P 22= 1.23725E-02
P 23= 1.23725E-02
P 24= 1.23725E-02
P 25= 1.23725E-02
P 26= 1.23725E-02
P 27= 1.23725E-02
P 28= 1.23725E-02
P 29= 1.23725E-02
P 30= 1.23725E-02
P 31= 1.23725E-02
P 32= 1.23725E-02
P 33= 1.23725E-02
P 34= 1.23725E-02
P 35= 1.23725E-02
P 36= 1.23725E-02
P 37= 1.23725E-02
P 38= 1.23725E-02
P 39= 1.23725E-02
P 40= 1.23725E-02
P 41= 1.23725E-02
P 42= 1.23725E-02
P 43= 1.23725E-02
P 44= 1.23725E-02
P 45= 1.23725E-02
P 46= 1.23725E-02
P 47= 1.23725E-02

P 48= 1.23725E-02
P 49= 1.23725E-02
P 50= 1.23725E-02
P 51= 1.23725E-02
P 52= 1.23725E-02
P 53= 1.23725E-02
P 54= 1.23725E-02
P 55= 1.23725E-02
P 56= 1.23725E-02
P 57= 1.23725E-02
P 58= 1.23725E-02
P 59= 1.23725E-02
P 60= 1.23725E-02
P 61= 1.23725E-02
P 62= 1.23725E-02
P 63= 1.23725E-02
P 64= 1.23725E-02
P 65= 1.23725E-02
P 66= 1.23725E-02

FOR RANDOM NUMBER N = 16 P1...PN IS

P 1= 0.281975
P 2= 7.35017E-02
P 3= 6.44357E-02
P 4= 5.13473E-02
P 5= 4.81585E-02
P 6= 4.81318E-02
P 7= 4.79674E-02
P 8= 4.78494E-02
P 9= 4.20870E-02
P 10= 4.20844E-02
P 11= 4.20791E-02
P 12= 4.20777E-02
P 13= 4.20764E-02
P 14= 4.20764E-02
P 15= 4.20763E-02
P 16= 4.20758E-02

FOR RANDOM NUMBER N = 19 P1...PN IS

P 1= 0.314583
P 2= 0.223709
P 3= 0.191845
P 4= 4.41510E-02
P 5= 2.03121E-02

P 6= 1.90026E-02
P 7= 1.84044E-02
P 8= 1.68237E-02
P 9= 1.62206E-02
P 10= 1.62027E-02
P 11= 1.41971E-02
P 12= 1.36525E-02
P 13= 1.32856E-02
P 14= 1.29510E-02
P 15= 1.29372E-02
P 16= 1.29365E-02
P 17= 1.29357E-02
P 18= 1.29353E-02
P 19= 1.29146E-02

FOR RANDOM NUMBER N = 44 P1...PN IS

P 1= 7.75298E-02
P 2= 4.82830E-02
P 3= 3.07304E-02
P 4= 3.01941E-02
P 5= 2.88415E-02
P 6= 2.14555E-02
P 7= 2.09169E-02
P 8= 2.03957E-02
P 9= 2.01698E-02
P 10= 2.00669E-02
P 11= 2.00487E-02
P 12= 2.00438E-02
P 13= 2.00428E-02
P 14= 2.00420E-02
P 15= 2.00414E-02
P 16= 2.00413E-02
P 17= 2.00413E-02
P 18= 2.00413E-02
P 19= 2.00413E-02
P 20= 2.00413E-02
P 21= 2.00413E-02
P 22= 2.00413E-02
P 23= 2.00413E-02
P 24= 2.00413E-02
P 25= 2.00413E-02
P 26= 2.00413E-02
P 27= 2.00413E-02
P 28= 2.00413E-02
P 29= 2.00413E-02

P 30= 2.00413E-02
P 31= 2.00413E-02
P 32= 2.00413E-02
P 33= 2.00413E-02
P 34= 2.00413E-02
P 35= 2.00413E-02
P 36= 2.00413E-02
P 37= 2.00413E-02
P 38= 2.00413E-02
P 39= 2.00413E-02
P 40= 2.00413E-02
P 41= 2.00413E-02
P 42= 2.00413E-02
P 43= 2.00413E-02
P 44= 2.00413E-02

FOR RANDOM NUMBER N = 56 P1...PN IS

P 1= 0.499734
P 2= 0.238736
P 3= 6.72054E-03
P 4= 5.73218E-03
P 5= 4.87431E-03
P 6= 4.83087E-03
P 7= 4.79491E-03
P 8= 4.79037E-03
P 9= 4.79009E-03
P 10= 4.78859E-03
P 11= 4.78850E-03
P 12= 4.78779E-03
P 13= 4.78768E-03
P 14= 4.78717E-03
P 15= 4.78709E-03
P 16= 4.78708E-03
P 17= 4.78708E-03
P 18= 4.78707E-03
P 19= 4.78707E-03
P 20= 4.78707E-03
P 21= 4.78707E-03
P 22= 4.78707E-03
P 23= 4.78707E-03
P 24= 4.78707E-03
P 25= 4.78707E-03
P 26= 4.78706E-03
P 27= 4.78706E-03
P 28= 4.78706E-03

P 29= 4.78706E-03
P 30= 4.78706E-03
P 31= 4.78706E-03
P 32= 4.78706E-03
P 33= 4.78706E-03
P 34= 4.78706E-03
P 35= 4.78706E-03
P 36= 4.78706E-03
P 37= 4.78706E-03
P 38= 4.78706E-03
P 39= 4.78706E-03
P 40= 4.78706E-03
P 41= 4.78706E-03
P 42= 4.78706E-03
P 43= 4.78706E-03
P 44= 4.78706E-03
P 45= 4.78706E-03
P 46= 4.78706E-03
P 47= 4.78706E-03
P 48= 4.78706E-03
P 49= 4.78706E-03
P 50= 4.78706E-03
P 51= 4.78706E-03
P 52= 4.78706E-03
P 53= 4.78706E-03
P 54= 4.78705E-03
P 55= 4.78706E-03
P 56= 4.78706E-03
